# *Octopus vulgaris* (Cuvier, 1797) in the Mediterranean Sea: Genetic Diversity and Population Structure

**DOI:** 10.1371/journal.pone.0149496

**Published:** 2016-02-16

**Authors:** Daniele De Luca, Gaetano Catanese, Gabriele Procaccini, Graziano Fiorito

**Affiliations:** Stazione Zoologica Anton Dohrn, Villa Comunale, 80121, Napoli, Italy; University of Padova, ITALY

## Abstract

The common octopus, *Octopus vulgaris* Cuvier 1797, is a largely exploited cephalopod species in the Mediterranean Sea and the Atlantic Ocean, as well as along the coasts of Africa, Brazil and Japan, where its taxonomic identity is still debated. The assessment of its genetic structure is a pressing need to correctly manage the resource and to avoid overfishing and collapsing of local stocks. Here we analysed genetic variation and population structure of *O*. *vulgaris* using thirteen microsatellite loci in seven sampling localities from the Mediterranean Sea and one from the Atlantic Ocean. We also used a DNA barcoding approach by COI gene fragment to understand the phylogenetic relationships among the specimens here investigated and the ones whose sequences are available in literature. Our results reveal high levels of allelic richness and moderate heterozygosity in all samples investigated, and a pronounced differentiation of the Atlantic and Sicilian specimens. This latter aspect seems to support the isolation of the biota within the Strait of Messina. A certain degree of differentiation was detected among the other geographic samples within the Mediterranean Sea, which is more compatible with an island model than isolation by distance. The occurrence of null alleles affected more genetic diversity indices than population structure estimations. This study provides new insights about the genetic diversity and structure of *O*. *vulgaris* in the area of interest, which can be used as guidelines for a fisheries management perspective.

## Introduction

Marine species show contrasting patterns of population structure due to the elevated number of intervening factors. The common view of marine populations as demographically open units has been questioned over the past decades [1] and evidence is accumulating for more complex scenarios [2]. Interactions among physical and biological factors, such as marine current patterns, sea bottom topology, and dispersal capability of the species at any biological stage (gametes, larvae, juveniles, adults) can account for different levels and patterns of gene flow, requiring *ad hoc* assessments for each specific case [3–6].

Among invertebrates, molluscs include sessile, slow walking or fast swimming species characterised by internal or external fertilization and direct or indirect development, living in a wide variety of habitats such as open sea, rocky or sandy bottoms. Several patterns of genetic structure have been identified, from genetic homogeneity [7,8] to isolation by distance [9,10], and island models of isolation [11]. Due to their short life-cycles, variable temperature- [12–14] and food availability-dependent growth rate, cephalopod stocks are highly unstable, being susceptible to overfishing and sensitive to environmental changes [15].

The concepts of differentiation, population structure and stock boundaries are strictly linked; a stock is commonly defined as “a group of organisms whose demographic/genetic trajectory is largely independent from other such groups” [1]. Since many mollusc species also represent important fishery resources, assessment of genetic structure can give important insights in stock management [16,17]. The insufficient knowledge on population boundaries does not allow to design adequate strategies for stock management and for improvement of aquaculture techniques (e.g. [18]), which would be essential to preserve the species from local collapse or extinction.

In the last few years, the decline of other fishery resources has led to an increase of cephalopod exploitation, especially in Europe [19], making a better knowledge of cephalopod ecology and life history traits a priority.

Studies dealing with genetic structure of cephalopods have increased over years [20–26], but the available information in many geographic regions is still scarce. In the Mediterranean Sea and the nearby Atlantic Ocean (the Eastern portion interesting European and African coasts), for example, few studies have investigated the genetic structure of cuttlefishes and squids (e.g. [27,28]), while more contributions exist on the common octopus (*Octopus vulgaris* Cuvier, 1797) ([29–34]; reviewed in [35]). However, the use of different molecular markers in not overlapping sampling sites led to several uncertainties. All these studies, together with others from different geographic regions, agree in finding genetic structure at different scales [33], generally not related to geographic distance among samples [29,36], although a latitudinal gradient along the Atlantic coasts of the Iberian Peninsula and Canary Islands was found [34]. If population structure is more influenced by passive movement of paralarvae through marine currents, or by active migration of adults, is still unclear.

*O*. *vulgaris* lifecycle spans from 9 to 15 months [37,38], the 10–15% of which represented by the planktonic phase at paralarval stage [39] and sexual maturity reached in a few months [32]. Since adult dispersal seems to be limited to forage habits (around a 15 m diameter, [40]) and short inshore and offshore migrations due to spawning behaviour [41], the major contributions to gene flow is supposed to depend on currents-driven paralarval movements. However, also climate change can directly affect gene flow and consequently population structure at both juvenile and adult level, determining critic fluctuations in stock abundance, or indirectly, through currents intensity and direction changes [42–44]. In this work we use microsatellite markers [45,46] to assess genetic diversity and structure of the common octopus, *O*. *vulgaris*, in the Mediterranean Sea and the nearby Atlantic Ocean. We also use assignment test to assess connectivity among geographic samples and we calculate the effective population size, for fishery and management purposes. Furthermore, we use COI sequences as DNA barcode to confirm the taxonomic assignment of the specimens here investigated and to explore their phylogenetic relationships.

## Materials and Methods

### Sampling

**Fig 1:**
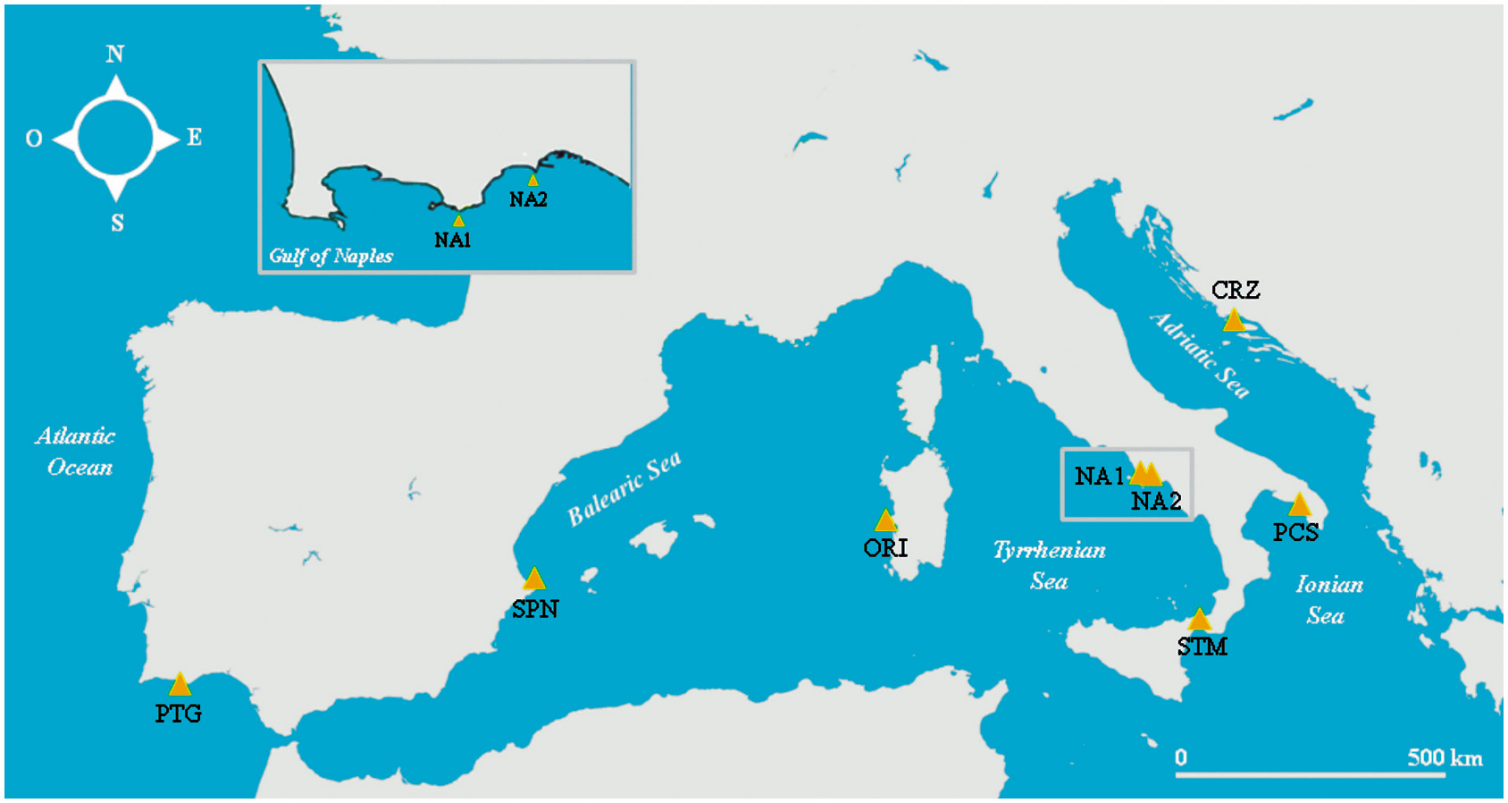
Map of sampling localities. Eight geographic samples were collected across the Mediterranean Sea and the Atlantic Ocean. See Table 1 for geographic coordinates and other information.

All the analyses have been carried out using preserved or freshly dead specimens collected from local fishermen. No use of live animals has been required for this study and no specific permissions were needed for the sampling activities in all of the investigated areas because our species of interest is commercially harvested (not endangered nor protected) and it was caught in areas where fishing is allowed. A total of 193 individuals were collected in seven localities across the Mediterranean Sea and in one locality in the nearby Atlantic Ocean (Fig 1; see Table 1 for details).

**Table 1:** Characteristics of sampling sites, including geographic information and genetic diversity indices per geographic sample. N: number of individuals sampled in each site; N_pa_: number of private alleles; A_r_: allelic richness per geographic sample; H_e_ and H_o_ indicate expected and observed heterozygosity respectively; *F*_IS_ is the inbreeding coefficient calculated in each population sample. In italics values after correction for null alleles.

Region	Geographicsample	Code	Coordinates	N	N_pa_	A_r_	H_e_	H_o_	*F*_IS_
Atlantic Ocean	Portugal,Ria Formosa	PTG	36°49’16.76”N7°53’7.73” O	25	23	8.115 ± 0.436*8*.*738 ±0*.*227*	0.667*0*.*693*	0.501*0*.*655*	0.271***0.055*
Mediterranean(Balearic Sea)	Spain,Balearic Sea	SPN	39°02’59.43” N0°10’27.54” E	26	4	7.846 ± 0.235*8*.*154 ± 0*.*235*	0.678*0*.*690*	0.498*0*.*654*	0.278***0.054*
Mediterranean(Sea of Sardinia)	Italy,Gulf of Oristano	ORI	39°48’46.72” N8°28’37.47” E	25	5	7.715 ± 0.185*8*.*008 ± 0*.*172*	0.683*0*.*699*	0.459*0*.*637*	0.341***0.090***
Mediterranean(Tyrrhenian Sea)	Italy,Gulf of Naples	NA1	40°47’8.31” N14°10’54.14” E	25	6	7.846 ± 0.140*8*.*123 ± 0*.*236*	0.642*0*.*665*	0.523*0*.*628*	0.204***0.058*
Mediterranean(Tyrrhenian Sea)	Italy,Gulf of Naples	NA2	40°49’22.10” N14°15’0.86” E	25	7	8.100 ± 0.178*8*.*492 ± 0*.*259*	0.649*0*.*682*	0.517*0*.*640*	0.223***0.062*
Mediterranean Sea	Italy,Strait of Messina	STM	38°14’51.81” N15°38’19.90” E	17	2	5.615 ± 0.000*6*.*462 ± 0*.*000*	0.600*0*.*633*	0.493*0*.*615*	0.207**0.*029*^*ns*^
Mediterranean(Ionian Sea)	Italy,Porto Cesareo	PCS	40°14’42.51” N17°52’20.49” E	25	5	7.092 ± 0.232*7*.*669 ± 0*.*274*	0.634*0*.*662*	0.492*0*.*615*	0.241***0.071**
Mediterranean(Adriatic Sea)	Croatia,Split	CRZ	43°23’58.25” N16°24’54.48” E	25	3	7.300 ± 0.319*7*.*715 ± 0*.*438*	0.660*0*.*681*	0.548*0*.*631*	0.190***0.075**

Asterisks indicate the p value of each pair-wise comparison:

* p < 0.05;

** p < 0.01;

*** p < 0.001; ns = not significant.

According to [47], we sampled whenever possible about 25 individuals per sampling site (assumed as geographic samples). Samples collected from the Gulf of Naples (central Tyrrhenian Sea, Italy) in two geographical locations distant less than 7 km, were included to test for fine-scale population structuring due to differences in the sea-bottom landscape [48,49]. Adult octopuses were caught by local fishermen not far from the coast. For each individual, a piece of arm (2–3 cm) was excised and stored in 95–100% ethanol until DNA extraction.

### DNA extraction

DNA was extracted using the NucleoSpin^®^ Tissue kit (Macherey-Nagel). Starting from the tissue stored in alcohol, about 25 mg were cut, freed of outer skin and put in distilled water for a while to account for rehydration and alcohol removal. Then, the tissue was chopped in small pieces, put in 1.5 mL tubes and processed following the manufacturer’s instructions. Resulting DNA was quantified by NanoDrop Spectrophotometer (ND-1000, Thermo Scientific) and diluted to the optimal concentration for each downstream application.

### DNA Barcoding with COI fragment

Despite *O*. *vulgaris* is easily recognisable from other octopus species of the Mediterranean Sea and the nearby Atlantic Ocean, a DNA barcode with a fragment of the COI gene was obtained for five individuals from each geographic sampling locality, using the primers developed by [50]. DNA amplification was carried out at the condition specified in [50]. PCR products were run on a 1.5% agarose gel, extracted in slices from the gel and purified using the GenElute^™^ Gel Extraction Kit (Sigma-Aldrich). DNA fragments were sequenced using LCO1490 primer [50] by Big-Dye Terminator Cycle sequencing kit (Applied Biosystems). The sequences obtained were compared with the ones available on public repositories such as GenBank and BOLD (Barcode Of Life Database) for taxonomic identification, and then aligned using the ClustalW algorithm [51] implemented in BioEdit v7.2.5 (http://www.mbio.ncsu.edu/bioedit/bioedit.html), together with other sequences of *O*. *vulgaris* with known geographical provenance [31, 52–60] (S1 Dataset). This dataset was used to build a Maximum Likelihood tree in MEGA6 [61] at the following parameters: GTR + G + I model with 4 gamma categories, 1000 bootstrap replications. The COI sequence of *O*. *insularis* was used as outgroup, due to its phylogenetic closeness to *O*. *vulgaris* [59].

### Microsatellite amplification

Microsatellite amplification was carried out combining 13 loci (previously tested in simplex, [45,46]) in two multiplex PCR reactions (Table 2), using the Type-it^®^ Microsatellite PCR kit (Qiagen) at the following conditions: 15 min at 95°C, 35 cycles at 94°C for 30 s, 59°C for 1 min and 30 s, 72°C for 1 min, and a final extension at 60°C for 30 min. Each reaction was conducted in a final volume of 12.5 μL. Forward primers were 5’ labelled with different fluorescent dyes (Life Technologies, see Table 2). PCR products were run on the Automated Capillary Electrophoresis Sequencer 3730 DNA Analyzer (Applied Biosystems) after a 1:300 dilution.

**Table 2:** Description of microsatellite loci utilised in this work. Abbreviations: N_a_ (number of alleles per locus), A_r_ (allelic richness), H_e_ (expected heterozygosity), H_o_ (observed heterozygosity). In italics values corrected for null alleles. The column Multiplex refers to the combinations of loci for PCR amplification.

Locus	Motif	Multiplex	Primer Tag	N_a_	Allele size (bp)	A_r_	H_e_	H_o_	Reference
Vulg06	(CA)_13_	I	VIC	12	183–205	7.991*7*.*868*	0.796*0*.*794*	0.725*0*.*814*	[46]
Vulg15	(GA)_17_	I	PET	10	205–227	5.938*5*.*860*	0.650*0*.*650*	0.544*0*.*617*	[46]
Vulg12	(CA)_18_	I	6FAM	7	242–262	4.166*4*.*087*	0.502*0*.*502*	0.321*0*.*409*	[46]
Vulg13	(TG)_11_	I	VIC	7	313–333	4.820*5*.*005*	0.646*0*.*661*	0.368*0*.*539*	[46]
Vulg04	(GT)_13_	II	NED	7	151–175	3.888*3*.*864*	0.251*0*.*247*	0.187*0*.*202*	[46]
Vulg07	(CA)_27_	II	6FAM	26	150–232	14.225*14*.*206*	0.913*0*.*915*	0.731*0*.*839*	[46]
Vulg10	(CA)_15_	II	VIC	6	207–219	3.343*3*.*344*	0.621*0*.*638*	0.332*0*.*492*	[46]
Vulg11	(CA)_18_	II	PET	16	225–271	6.651*6*.*705*	0.753*0*.*759*	0.643*0*.*762*	[46]
Vulg14	(CAT)_6_	II	NED	8	352–409	3.864*3*.*821*	0.309*0*.*310*	0.254*0*.*285*	[46]
oct03	(AT)_16_ (GT)_15_	I	PET	29	115–193	16.439*15*.*752*	0.939*0*.*939*	0.521*0*.*871*	[45]
oct08	(TG)_36_	I	6FAM	32	112–176	15.947*15*.*725*	0.935*0*.*934*	0.594*0*.*860*	[45]
Ov10	(GA)_14_	II	VIC	19	168–206	10.390*10*.*310*	0.816*0*.*818*	0.684*0*.*736*	[45]
Ov12	(GATA)_20_	II	PET	27	172–380	12.879*12*.*779*	0.905*0*.*905*	0.653*0*.*834*	[45]

### Microsatellite analysis and genetic diversity

Microsatellite peaks were analysed with Peak Scanner^™^ Software v1.0 (Life Technologies) and scored as PCR products; in case of ambiguity in assigning sizes, samples were run at different dilutions or re-amplified. Micro-Checker v2.2.3 [62] was used to detect typographic errors, whilst MicroDrop v1.01 [63] to estimate and correct for allelic dropout rate. The software MicroDrop first calculates the correlation between the amount of missing data and homozygotes among individuals and loci, then fits the results of this analysis into a model and finally creates a new dataset drawing genotypes according to the posterior probabilities of the model. The resulting dataset was used jointly with the original one to evaluate the impact of null alleles on genetic diversity and population structure estimations.

Putative loci under selection were detected with LOSITAN [64], a selection detection workbench based on the *F*_ST_-outlier method. The options “Neutral mean *F*_ST_” and “Force mean *F*_ST_” were used, to increase the reliability of the estimate of the initial mean *F*_ST_ computed from the empirical dataset. A total of 50,000 simulations were computed, plus 5,000 additional, as suggested by the default settings of the software. To test the power of our microsatellite markers on discriminating individuals using multilocus genotypes, we used the software GENCLONE v2.0 [65]. Number of alleles (N_a_) per locus and inbreeding coefficient (*F*_IS_) per population were calculated in Arlequin v3.5.1.3 [66], while *F*_IS_ per locus [67] was calculated in Genepop on the Web [68,69]. Private alleles were detected using GenAlEx v6.501 [70,71]. Expected (H_e_) and observed heterozygosity (H_o_) per locus and population were calculated using GENETIX v4.05.2 [72]. Linkage disequilibrium was tested for each pair of loci in each population using Genepop on the Web [68,69]; the statistical significance of the pair-wise comparisons was determined using the Bonferroni correction for multiple tests.

Hardy-Weinberg equilibrium (HWE) probabilities per locus and population were computed in Genepop on the Web [68,69] both on the original dataset and the one corrected for null alleles; parameters utilised: 10,000 dememorisations, 100 batches and 10,000 iterations per batch. Allelic richness per population, based on minimum sample size (17 individuals) was calculated using standArich v1.00 [73], a package running in R [74].

Relationships among individuals within each sampling locality were calculated using ML-Relate [75] both on the original dataset and the one corrected for null alleles. A two tailed t-test was applied to verify if the results obtained without correcting for null alleles were significantly different from those calculated using correction.

### Genetic differentiation and population structure

Genetic differentiation among geographic samples was calculated using Weir and Cockerham’s *F*_ST_ [67] as implemented in Arlequin v3.5.1.3 [66], and Jost’s D [76], using the package DEMEtics [77] implemented in R [74]. In general, *F*_ST_ measures deviation from a condition of panmixia and is calculated on allele frequencies, whilst D measures deviation from total differentiation and is more related to genetic distances among populations [78]. We set up the parameters for Jost’s D as follows: pair-wise comparisons and p-values as statistics to test against the null hypothesis of no genetic differentiation (based on 1,000 bootstrap resamplings). Bonferroni correction for multiple tests was performed for both *F*_ST_ and Jost’s D estimators. To test if the observed patterns of genetic structure conformed to the isolation by distance model, we correlated population pair-wise’s *F*_ST_ previously calculated with the geographic distance (km) between each pair of sampling locality, using the Mantel test function implemented in XLSTAT (Addinsoft, trial version; as add-ins running in Microsoft Excel). Geographical distances were calculated using Google Earth v7.0 (https://www.google.com/earth/), drawing a curve line moving along the coasts according to the circulation of water masses in the Mediterranean Sea [79,80]. We decided to follow this criterion to calculate geographic distances since seasonal adult octopus migrations, where studied, resulted to be very limited [38] and few data about paralarvae migrations are available [81].

After preliminary results, we decided to perform a discriminant analysis of principal components (DAPC) using the R working package ADEGENET v2.0.0 [82] to cluster our samples in groups, due to the peculiarity of multivariate methods of working in a context free of assumptions of Hardy-Weinberg equilibrium and linkage disequilibrium. We tested the reliability of our results using the α score function.

We also used GENECLASS2 [83] to assign individuals to sampling localities into a framework that does not require populations being in HWE, which best fits our data. We used the frequencies-based method [84], with the default frequency for missing alleles (0.01) as suggested by [85], and enabling computation probability under the simulation algorithm described in [85] (parameters applied: 1,000,000 simulated individuals, type I error set at 0.01).

Contemporary effective population size (Ne) from multilocus diploid genotypes was estimated using both the method based on linkage disequilibrium data [86] and the one based on molecular co-ancestry [87], as implemented in the software NeEstimator v2.01 [88]. Both methods calculate the number of effective breeders (N_eb_) of a cohort from which a sample is obtained that, in populations with non-overlapping generations (such as ours, with rare exceptions), is nearly equal to Ne. For the linkage disequilibrium method, a value of 0.020 was set as the lowest allele frequency used. We used preliminary results of population structure analyses to pool together homogeneous samples before calculating Ne, identifying as homogeneous only the geographic samples that were significantly different from all the others and pooling together the remaining.

## Results

### DNA Barcoding

All subsamples analysed were unambiguously attributed to *O*. *vulgaris*. The 482 bp DNA sequences of the COI region considered in the analysis were identical among all samples from Mediterranean Sea and nearby Atlantic Ocean collected in the present study. Three main clades are present in the ML phylogenetic tree obtained (S1 Fig). The first clade (93% bootstrap) harbours most of the Mediterranean samples and few samples from the nearby Atlantic Ocean (Portugal and Spain), Western and Southern Africa, the Southern Atlantic Ocean (Tristan da Cunha), and the Southern Indian Ocean (Amsterdam and Saint Paul Islands), a second clade (100% bootstrap) harbours samples from South-East Asia and a third one includes samples from Brasil. Samples from Turkey are present both in the Mediterranean and in the Asian clades.

### Genetic diversity

All microsatellite loci were successfully amplified in our specimens, except for the locus oct03 in two individuals from the Gulf of Oristano (Italy; Mediterranean Sea) and one from the Balearic Sea (Spain) which also did not result to be successful at locus oct08. We decided to treat these as missing data and included the samples in subsequent analyses. Genotypes for each individual are provided in S2 Dataset. Only locus Vulg10 was found to be a putative “borderline” candidate under positive selection (S2 Fig); after checking that the results of downstream analyses were not affected by the presence of this locus, it was maintained in the final dataset.

A total of 193 unique multilocus genotypes were found over 193 individuals analysed, even after removing loci with missing data.

No correlation (Pearson’s) between amount of homozygotes and amount of missing data was found neither across individuals (r = 0.024, p = 0.36) nor across loci (r = -0.072, p = 0.57), excluding the occurrence of allelic dropout in the dataset. On the contrary, null alleles were detected at all loci, the most at very low frequencies (< 1%), and few around 10–15% (S1 Table). The impact of null alleles is evident in the significant deviations from Hardy-Weinberg equilibrium that were mostly reduced after correction. Indeed, 35 pair-wise locus comparisons that were not in HWE were recovered (Table 3), with locus Vulg06 being in HWE in all of the investigated geographic samples (Table 3). However, null alleles seem unlikely to be the only factor responsible for such pattern of disequilibrium, that otherwise should have been restored after correction.

**Table 3:** P values of Hardy-Weinberg equilibrium. Values in italics refer to the dataset corrected for null alleles. See Table 1 for abbreviations.

	PTG	SPN	ORI	NA1	NA2	STM	PCS	CRZ
**Vulg06**	0.029*0*.*494*	0.171*0*.*226*	0.274*0*.*441*	0.864*0*.*865*	0.196*0*.*617*	0.010*0*.*075*	0.033*0*.*442*	0.630*0*.*839*
**Vulg15**	0.031*0*.*209*	0.020*0*.*190*	0.005*0*.*055*	0.109*0*.*108*	0.289*0*.*579*	0.003*0*.*013*	1.000*1*.*000*	0.957*0*.*959*
**Vulg12**	0.001*0*.*012*	0.001*0*.*260*	0.004*0*.*107*	0.035*0*.*041*	0.179*0*.*201*	0.000*0*.*000*	0.405*0*.*094*	1.000*0*.*958*
**Vulg13**	0.000*0*.*076*	0.001*0*.*022*	0.000*0*.*005*	0.434*0*.*414*	0.526*0*.*829*	0.005*0*.*097*	0.003*0*.*265*	0.000*0*.*027*
**Vulg04**	0.387*0*.*482*	0.000*0*.*002*	1.000*1*.*000*	1.000*1*.*000*	1.000*1*.*000*	1.000*1*.*000*	0.040*0*.*041*	1.000*1*.*000*
**Vulg07**	0.006*0*.*269*	0.027*0*.*039*	0.000*0*.*017*	0.016*0*.*059*	0.816*0*.*752*	0.001*0*.*001*	0.010*0*.*058*	0.730*0*.*625*
**Vulg10**	0.000*0*.*016*	0.000*0*.*112*	0.000*0*.*101*	0.002*0*.*335*	0.232*0*.*195*	0.015*0*.*014*	0.000*0*.*006*	0.000*0*.*001*
**Vulg11**	0.212*0*.*337*	0.188*0*.*016*	0.000*0*.*212*	0.889*1*.*000*	0.050*0*.*193*	0.000*0*.*001*	0.962*0*.*916*	0.142*0*.*145*
**Vulg14**	0.020*0*.*036*	0.013*0*.*187*	0.007*0*.*034*	0.753*0*.*825*	0.251*0*.*252*	-*1*.*000*	0.489*0*.*483*	0.284*0*.*284*
**oct03**	0.000*0*.*301*	0.000*0*.*060*	0.000*0*.*976*	0.000*0*.*005*	0.000*0*.*183*	0.000*0*.*052*	0.000*0*.*009*	0.000*0*.*000*
**oct08**	0.015*0*.*758*	0.001*0*.*652*	0.001*0*.*074*	0.000*0*.*180*	0.000*0*.*001*	0.000*0*.*112*	0.000*0*.*204*	0.000*0*.*000*
**Ov10**	0.142*0*.*551*	0.019*0*.*023*	0.437*0*.*691*	0.005*0*.*300*	0.321*0*.*574*	0.000*0*.*000*	0.142*0*.*316*	0.640*0*.*790*
**Ov12**	0.000*0*.*003*	0.160*0*.*930*	0.000*0*.*002*	0.025*0*.*102*	0.000*0*.*064*	0.000*0*.*000*	0.001*0*.*683*	0.040*0*.*157*

Linkage disequilibrium between loci was only found in two population samples after Bonferroni correction: Croatia, with two couples of loci in linkage (Vulg06 –Ov12; oct08 –Ov12), and Strait of Messina, where each locus was in linkage with at least another one (S2 Table). The high number of loci in linkage disequilibrium in Sicilian specimens is likely due to the occurrence of population structure and inbred individuals (see below) in this sample. Due to this “population” specific pattern, we decided to keep all loci for further analyses.

Microsatellite loci used in our dataset appeared to be moderately to highly polymorphic, with a number of alleles per locus ranging from 6 (Vulg10) to 32 (oct08) and observed heterozygosity values spanning from 0.187 to 0.731 (Table 2). However, it seems that H_o_ values per locus are affected by the occurrence of null alleles, as attested by the increasing of values after correction (from 0.202 to 0.871, Table 2).

Fifty-five private alleles were detected (Table 1), the most belonging to the Atlantic grouping (23), with a frequency ranging from 0.019 to 0.176 (S3 Table). Genetic diversity indices were quite homogeneous among all geographic samples (Table 1). Observed heterozygosity values per population were always slightly lower than expected, indicating excess of homozygosity, which correlate with the significant and comparable positive values of *F*_IS_ per locus (Table 4) and geographic samples (Table 1). Significant differences were found in H_o_ estimates per locus and sample comparing the dataset corrected and not corrected for null alleles (p < 0.01, two-tailed Wilcoxon test).

**Table 4:** *F*_IS_ estimations per locus with and without correction for null alleles.

Locus	Uncorrected	Corrected
**Vulg06**	0.091	-0.025
**Vulg15**	0.166	0.051
**Vulg12**	0.363	0.186
**Vulg13**	0.433	0.186
**Vulg04**	0.260	0.183
**Vulg07**	0.202	0.083
**Vulg10**	0.468	0.229
**Vulg11**	0.149	-0.004
**Vulg14**	0.182	0.081
**oct03**	0.447	0.073
**oct08**	0.367	0.080
**Ov10**	0.165	0.101
**Ov12**	0.281	0.078

Similarly to the case of HWE, inbreeding estimates corrected for null alleles were still positive (average 0.100 per locus and 0.062 per geographic sample), although lower than those calculated with the original dataset (average 0.275 per locus and 0.244 per geographic sample).

Allelic richness (A_r_) per population ranged from 5.615 to 8.115, with the lowest value observed in the samples from the Strait of Messina (Table 1).

Related individuals using ML-Relate were found in all samples (S4 Table). The highest frequencies of full-sibs (FS) pairs were present in the geographic sample from the Strait of Messina (0.22059), whilst comparable frequencies of half-sibs (HS) were detected in all the other samples (S4 Table). The high frequency of HS in the geographic sample of Ria Formosa was reduced after correction for null alleles (S4 Table). Relationship pairs calculated with and without accounting for null alleles were not significantly different (p > 0.50), indicating that these results are not affected by the non-amplification of alleles.

### Population differentiation and genetic structure

The estimation of genetic differentiation between geographic samples gave slightly different results according to the coefficient used, with Jost’s D being more conservative than Weir and Cockerham’s *F*_ST_. Most of the statistically significant pair-wise comparisons were shared between the two coefficients, with the exception of the comparison Porto Cesareo—Spain (Table 5). Samples showing the highest differentiation, with statistically significant values for both indices in all pair-wise comparison with all the others, were Ria Formosa (PTG) and Strait of Messina (STM). Conversely, not significant genetic differentiation was found with both methods in the pair-wise comparison of the two geographic samples from the Gulf of Naples (*F*_ST_ = -0.00390, p > 0.05; D_est_ = 0.00177, p > 0.05) and between the Adriatic and Ionian specimens, Porto Cesareo and Croatia (*F*_ST_ = 0.00540, p > 0.05; D_est_ = 0.03085, p > 0.05) (Table 5). Significant differentiation was also found between the Adriatic specimens from Croatia (eastern Mediterranean Sea) and the ones from the Balearic Sea (Spain, western Mediterranean Sea) after Bonferroni correction using both indices (Table 5). No significant differences were found among geographic samples from Spain (SPN), the Gulf of Naples (NA1 and NA2), the Gulf of Oristano and Croatia with both indices (Table 5).

**Table 5:** Matrix of pair-wise *F*_ST_ (below the diagonal) and pair-wise Jost’s D (above the diagonal).

	PTG	SPN	ORI	NA1	NA2	STM	PCS	CRZ
**PTG**	-	0.18821***	0.21011***	0.24603***	0.22197***	0.42873***	0.23907***	0.24924***
**SPN**	0.05318***	-	0.05436*	0.06396*	0.04896^ns^	0.24693***	0.08794***	0.09619***
**ORI**	0.05533***	0.02089**	-	0.03999^ns^	0.06408*	0.26234***	0.06780**	0.08201**
**NA1**	0.08018***	0.02486**	0.02754***	-	0.00177^ns^	0.31000***	0.04848^ns^	0.09086**
**NA2**	0.07965***	0.02322**	0.03456***	-0.00390^ns^	-	0.31306***	0.04883^ns^	0.08207***
**STM**	0.12788***	0.08462***	0.07830***	0.10510***	0.10831***	-	0.27674***	0.21789***
**PCS**	0.07838***	0.02537**	0.02078*	0.01637*	0.01825**	0.09075***	-	0.03085^ns^
**CRZ**	0.07437***	0.03312***	0.01874*	0.03350***	0.03544***	0.06586***	0.00540^ns^	-

Asterisks indicate the p value of each pair-wise comparison:

* p < 0.05;

** p < 0.01;

*** p < 0.001; ns = not significant; in bold significant values after Bonferroni correction (p < 0.0017672). For abbreviations utilised see Table 1.

Population differentiation estimations inferred using Weir and Cockerham’s *F*_ST_ did not change significantly using the dataset corrected for null alleles (S5 Table); on the contrary, only differentiation among Ria Formosa and Strait of Messina in respect to all the other sampling localities was retained using Jost’s D.

Despite several significant pair-wise comparisons, no isolation by distance was found using the Mantel test (R^2^ = 0.010, p = 0.623, see S3 Fig).

**Fig 2:**
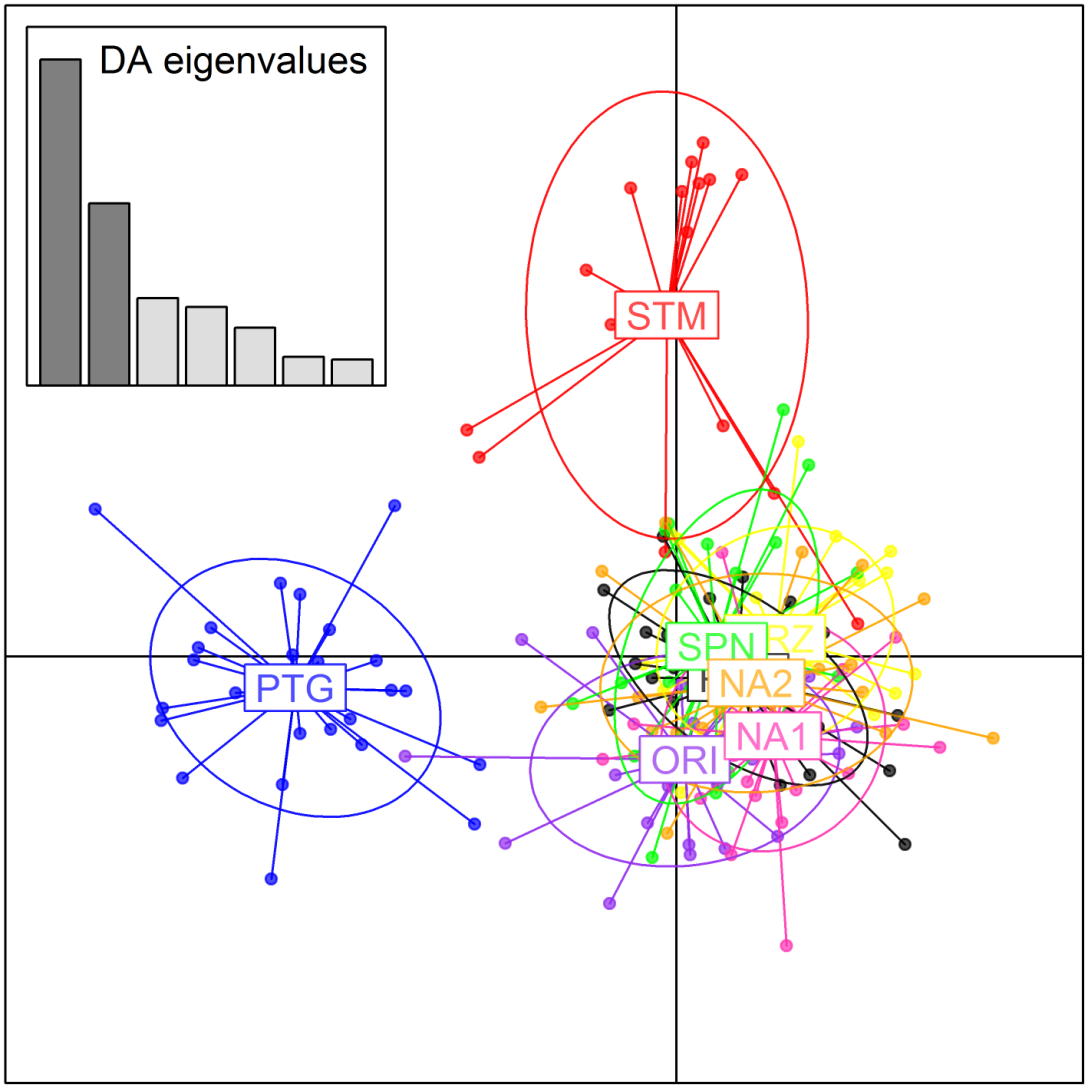
Discriminant analysis of principal components (DAPC). Geographic samples are encompassed by ellipses. See Table 1 for abbreviations.

The DAPC plot confirmed a strong distinction between samples from Ria Formosa and the Strait of Messina, and an overlapping pattern for the other geographic specimens from the Mediterranean Sea (Fig 2). Loci Vulg15, Ov10 and Ov12 were identified as the ones mostly contributing to the observed variation. No differences in the distribution of individuals within clusters were detected using the α-score function. A similar output was obtained with the dataset corrected for null alleles (S4 Fig).

**Fig 3:**
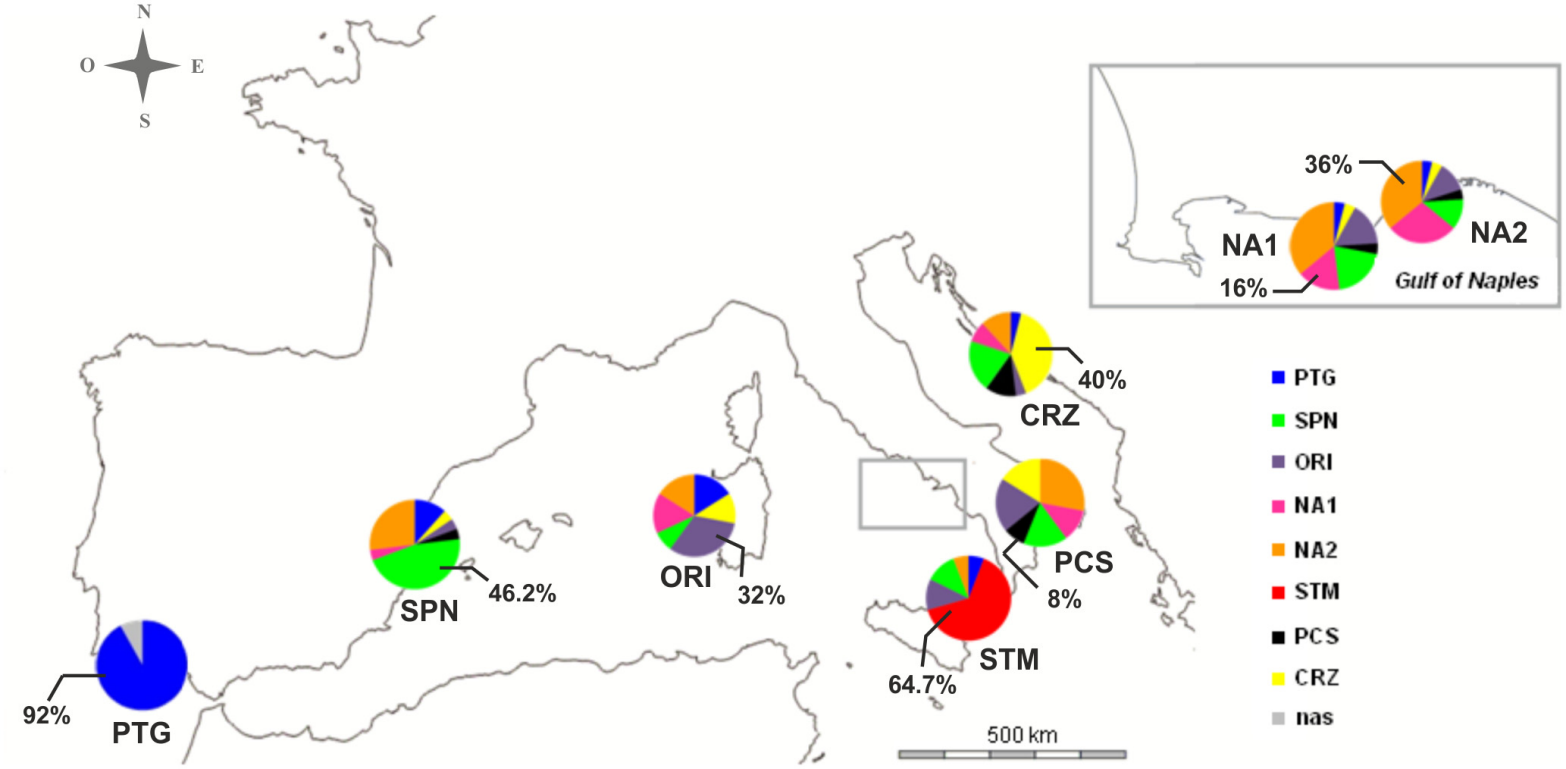
GENECLASS2 assignment test. In the pie graphs, each value indicates the percentage of individuals correctly assigned to the source sampling locality. nas indicates individuals that were not unambiguously assigned. See Table 1 for abbreviations.

The assignment test performed in GENECLASS2 (Fig 3) allowed to correctly assign 37.8% of individuals (73 out of 193) to their sampling locality, the most belonging to the Atlantic and Sicilian groupings. Mediterranean specimens (except for some from the Strait of Messina, in red) have admixed genotypes and can only be distinguished on the different proportions of membership to each sampling locality: the two population samples from the Gulf of Naples show only slight differences between each other.

The analysis conducted with NeEstimator showed differences in the estimation of contemporary genetic effective population size (Ne) with the two methods used. Estimations with the LD method varied greatly considering or not the correction for null alleles, with overall wide confidence intervals (S6 Table). The method based on molecular co-ancestry showed that the samples with the lowest number of breeders are Ria Formosa (8) and Strait of Messina (6–8): these are also the ones with the highest frequency of related individuals and showing strong genetic structure (S6 Table). Furthermore, the confidence intervals obtained with this approach are narrower than the ones with the LD method, and no large differences were detected with and without considering null alleles (S6 Table).

## Discussion

We used a population genetics approach by microsatellite markers to define population structure and genetic diversity of the common octopus *Octopus vulgaris* in the Mediterranean Sea and nearby Atlantic Ocean. Our results identify specimens from Ria Formosa (Atlantic Ocean) and the Strait of Messina (Mediterranean Sea) as distinct populations, as confirmed by the strong and significant differentiation detected with different methods of analysis. Conversely, the genetic differentiation among all the other specimens from the Mediterranean Sea is not straightforward, reflecting in the inability to correctly assign all the individuals to their locality of origin. Samples from the Adriatic and the Ionian Sea are homogeneous and no small-scale differentiation was detected in the Gulf of Naples. Our study also highlights higher levels of allelic richness but lower heterozygosity than other studies on the same species.

### Genetic diversity

Significant deviations from Hardy-Weinberg equilibrium (HWE) were detected in all samples analysed. Correcting the dataset for null alleles, 35 locus by locus evaluations that were not in HWE were recovered, with a general reduction of the inbreeding coefficient *F*_IS_ and two loci (Vulg06 and Vulg11) showing homozygotes deficit. Homozygosity excess due to null alleles is generally locus-specific [89], while in our analysis it was present across most of the loci (11 out of 13, considering null alleles correction), suggesting other factors being responsible for such pattern. The lowering of homozygosis after correction for null alleles is a clear signal of the impact of non-amplifying alleles, which hamper the estimates of genetic diversity.

Our *F*_IS_ values per population corrected for null alleles agree with expectations, according to a review on 124 invertebrate species, where reproductive traits were related with *F*_IS_ value [90]. Authors found that values of *F*_IS_ < 0.1 are observed in marine invertebrates with internal copulation or sperm transfer such as cephalopods, whilst higher values (*F*_IS_ > 0.3) in species with planktonic sperm (e.g. anthozoans, bivalves, gastropods, polychaetes and tunicates). In general, our geographic populations showed higher allelic richness but lower heterozygosity than other studies on the same species [33,34,36], while low values of heterozygosity were observed in other cephalopod species [27,91,92]. The moderate heterozygosity and positive *F*_IS_ values found in our sampling sites could be related to non-random mating within geographic populations, as shown by kinship analysis. On the contrary, an excess of heterozygosis was previously reported for Brazilian populations of *O*. *vulgaris* [36], indicating that reproductive behaviour can be different in different geographic areas, although authors suggest a bottleneck event as possible cause of the heterozygosity excess among most of loci [36].

High allelic diversity and low to moderate heterozygosity are contrasting patterns and are difficult to explain. It is possible that the fortuitous migration of individuals among populations (especially within the Mediterranean Sea) is responsible for high allelic richness and low number of private alleles, while the biology of the species accounts for the degree of homozygosity recorded. Small and intense bottleneck events cause substantial loss of allelic diversity, while diffuse bottlenecks spread over generations provoke the same loss of heterozygosity but a smaller reduction of allelic diversity [89]. On the other hand, overfishing causes reduction in effective population size and consequent decrease of allelic diversity and heterozygosity [93,94]. In our study, effective population size (Ne) was particularly low in Atlantic and Sicilian localities, followed by the group encompassing octopuses from Sardinia, Spain and the Gulf of Naples.

### Population structure

Our analysis of population structure in the Mediterranean Sea based on microsatellite markers agrees with the analysis conducted by Maltagliati and co-workers [29] using allozymes, in detecting sub-structuring among Mediterranean populations. The authors found populations from Sicily and Crete genetically distant from the ones in the western basin, with no clear structure among the other Mediterranean populations included in the analysis, that were all from the North-Western Mediterranean basin [29]. A further analysis conducted on a single microsatellite locus, which included the populations analysed by Maltagliati et al. [29], found significant structure within the whole Mediterranean basin [30].

Here we detected, in the Mediterranean, a strong isolation of the specimens from Strait of Messina, where the low values of H_o_, N_a_ and A_r_ are indicative of the occurrence of genetic drift. Furthermore, the high frequency of half-sibs detected in this sample strengthen its genetic structure. We are so confident in considering it as a distinct population. Moreover, we suggest the presence of a genetic subgrouping in the basin, supported by both differentiation indices used. Octopuses from the Adriatic and Ionian Sea are not genetically differentiated as well as the ones within the Gulf of Naples. We also found a partial isolation of the central and western basin, with the specimens from Croatia that are significantly different from the ones from Spain and the Gulf of Naples (NA2).

East and West Mediterranean populations appear separated in several marine species, as results of the glacial/post-glacial evolutionary history of the basin and present-day circulation patterns (e.g. [95] for seagrasses; [96] for fishes). We are not able to confirm this assumption; furthermore, according to our results, we can suppose that an island model of migration seems to be more likely to occur in the Mediterranean Sea for *O*. *vulgaris*, in respect to isolation by distance, as also suggested by [29].

As common for the most of marine species (e.g. [80]), we also found that the Atlantic samples (Ria Formosa) are well differentiated from the Mediterranean ones. In particular, in the Atlantic population, we found 23 private alleles, against the 2–7 private alleles found among the Mediterranean populations. It is likely that this skewed distribution of different alleles contributes to the observed differentiation. Assignment test was particularly effective in distinguishing Atlantic from Mediterranean specimens, but also highlighted the occurrence, within Mediterranean samples, of genotypes that were likely to belong to the Atlantic ones. Whether this pattern is the result of a recent migration mediated by ocean currents from the Atlantic Ocean to the Mediterranean Sea, or it is related to a past colonization from the Atlantic due to changes in sea level associated with cyclic ice ages during the Quaternary, is still to be assessed. The adult motility seems to be quite limited [32,38] and the dispersal of *O*. *vulgaris* paralarvae through marine currents is the most probable vehicle of long distance dispersal, although further investigations of the biology of the common octopus will help in discriminating between these scenarios. Mitochondrial data show differentiation between specimens from central and eastern Mediterranean [32], and within the Eastern basin [31], supporting the hypothesis that the roots of such genetic structure are historical. Our analysis showed that the COI gene is not a good mitochondrial marker to detect genetic structure, at least in the investigated area, contrasting with the findings of [31] along the coasts of Turkey. However, the ambiguous and multiple position of Turkish specimens along our phylogenetic tree highlighted the needing of further studies in that area.

The occurrence of genotypes that can only be assigned to the population from the Strait of Messina can be probably explained with the peculiar characteristics of the site. This is an area of strong currents generated by the opposition of tidal phases between the Tyrrhenian and Ionian basins [97]. These currents and vortices might isolate the biota of the Strait of Messina from the one of the Western basin or connect it with the Eastern basin through a unidirectional water mass flow. However, few studies are available in literature supporting this scenario [95]. It is now important to understand if this pattern is shared by other populations within the Strait and downstream, which would mean that Sicilian octopuses constitute a unique genetic unit in the whole basin, or if they are genetically closer to other specimens in areas of the Eastern Mediterranean. The genetic distinctiveness of the South Sicilian population of Porto Palo in [29], seems to support the first hypothesis.

Population genetic structure has been found in the common octopus also in other geographic areas at different spatial scales [31–34,36]. In some coastal areas, environmental parameters might give rise to barriers to gene flow, promoting differentiation. Along the coasts of Africa, strong seasonal upwelling phenomena are probably responsible for the differentiation of the Northwest African octopus fisheries [33,44]. On the contrary, along the southern coasts of Brazil, the structuring of 11 fishery sites into four genetic populations in absence of apparent oceanographic and geographic barriers is explained by bottleneck events [36]. In our study, we do not find significant differentiation at small scale (about 7 km; Gulf of Naples, Italy). The circulation of the water masses in the Gulf of Naples and relative migration patterns of the common octopus, may contribute to the observed homogeneity at small scale but not allowing connectivity at larger scales.

In the context of fishery management, particular attention should be paid on the octopus population of the Strait of Messina. The high number of loci in linkage disequilibrium, the small effective population size and the relatively low allelic richness are signs of genetic erosion in this population. Extensive fishing may have caused a reduction in effective population size through genetic drift and linkage disequilibrium among loci. Further analyses in this area using more individuals and extending the spatial scale could be of help to investigate the extent of such phenomenon.

The other homogenous groups from the Mediterranean Sea and the Atlantic Ocean derived by our analyses, show contrasting values of Ne according to the method used for estimation, making difficult to draw inferences.

The increasing trend in octopus catches in Europe but also in the world should focus the attention to this issue. According to FAO [98], European production of octopuses for 2010 was 42945 t, the most constituted by *Octopus vulgaris*, with some *Eledone cirrhosa* and *E*. *moschata*, from the western and central Mediterranean Sea and the Atlantic coasts of Iberian Peninsula. In particular, the five largest producers are Spain (16470 t), Portugal (10934 t), Italy (9884 t), Greece (2676 t), and France (1744 t). In Italy, 3295 t of *O*. *vulgaris* have been caught in 2012 (FAO FishStatJ, http://www.fao.org/fishery/statistics/software/fishstatj/en), showing a decline if compared with the previous years. In this scenario, understanding the genetic population structure of *O*. *vulgaris* as well as of other exploited species over time become fundamental in order to avoid a reduction of effective population size and consequently erosion of genetic diversity. We hope that the findings of our study will be the starting point for further investigations of genetic structure of the common octopus along its area of distribution.

## Supporting Information

S1 Dataset List of aligned COI sequences. Files collected from public repositories are identified with the relative code in parenthesis.
(DOCX)

S2 Dataset Microsatellite multilocus genotypes. (XLSX)

S1 Fig Maximum Likelihood tree based on COI sequences illustrating the evolutionary relationships among *O*. *vulgaris* specimens. Values at the basis of each branch (node) indicate the percentage of occurrence of such specimens together with the other ones in the bootstrap test (1000 replicates).
(PDF)

S2 Fig Outlier loci analysis conducted in LOSITAN. (TIF)

S3 Fig Mantel test. Correlation between geographic distance (km) and pairwise genetic differentiation (*F*_ST_).
(TIF)

S4 Fig Discriminant analysis of principal components (DAPC) using the dataset corrected for null alleles. Geographic samples are encompassed by ellipses. See Table 1 for abbreviations.
(TIF)

S1 Table Null alleles rate per locus. (DOCX)

S2 Table Significant pair-wise linkage disequilibrium p-values. Significance determined after Bonferroni adjustment at p < 0.000641.
(DOCX)

S3 Table Private alleles and their frequency in each geographic sample. (DOCX)

S4 Table Relationships among individuals. Abbreviations are as follows: U = unrelated, HS = half- sibs, FS = full-sibs, PO = parent/offspring. In italics are values corrected for null alleles.
(DOCX)

S5 Table Matrix of pair-wise *F*_ST_ (below the diagonal) and Jost’s D (above the diagonal). Asterisks indicate the p value of each pair-wise comparison: * p < 0.05; ** p < 0.01; *** p < 0.001; ns = not significant. In bold values significant after Bonferroni correction at p < 0.0017672. See Table 1 for abbreviations.
(DOCX)

S6 Table Contemporary effective population size (Ne) based on linkage disequilibrium and molecular co-ancestry models, with relative confidence intervals (CIs). Values in italics refer to the dataset corrected for null alleles.
(DOCX)
