# Supplementary material for: Octopus vulgaris (Cuvier, 1797) in the Mediterranean Sea: Genetic Diversity and Population Structure
Source: PLoS One. 2016 Feb 16;11(2):e0149496. doi: 10.1371/journal.pone.0149496 (PMC4755602; doi:10.1371/journal.pone.0149496)
Supplement: S1 Dataset — Files collected from public repositories are identified with the relative code in parenthesis. (DOCX) [file pone.0149496.s001.docx]

**S1 Dataset. List of aligned COI sequences.** Files collected from public repositories are identified with the relative code in parenthesis.

>Napoli_1_(NA1)_Italy_(present_study)

GTAATTGTTACAGCTCACGCATTTGTTATAATTTTTTTTCTTGTTATACCAGTTATAATTGGAGGATTTGGAAACTGATTAGTTCCTTTAATACTAGGAGCACCAGATATAGCATTCCCACGAATAAATAACATAAGCTTCTGACTCTTACCTCCTTCTCTCACTCTTCTCCTTTCATCTGCAGCTGTTGAAAGTGGTGCAGGTACCGGATGAACCGTTTACCCACCTCTTTCAAGAAATTTAGCTCATATAGGACCCTCTGTTGATCTAGCAATTTTCTCACTTCACTTAGCAGGTATTTCATCAATTCTTGGAGCCATCAATTTTATTACAACTATTATTAATATACGATGAGAAGGTATATTAATAGAACGACTTCCACTATTTGTGTGATCTGTTTTTATTACCGCAATTTTACTATTACTATCATTACCAGTACTCGCTGGAGCAATTACTATACTTTTAACTGACCGAAATTTTAA

>Napoli_2_(NA1)_Italy_(present_study)

GTAATTGTTACAGCTCACGCATTTGTTATAATTTTTTTTCTTGTTATACCAGTTATAATTGGAGGATTTGGAAACTGATTAGTTCCTTTAATACTAGGAGCACCAGATATAGCATTCCCACGAATAAATAACATAAGCTTCTGACTCTTACCTCCTTCTCTCACTCTTCTCCTTTCATCTGCAGCTGTTGAAAGTGGTGCAGGTACCGGATGAACCGTTTACCCACCTCTTTCAAGAAATTTAGCTCATATAGGACCCTCTGTTGATCTAGCAATTTTCTCACTTCACTTAGCAGGTATTTCATCAATTCTTGGAGCCATCAATTTTATTACAACTATTATTAATATACGATGAGAAGGTATATTAATAGAACGACTTCCACTATTTGTGTGATCTGTTTTTATTACCGCAATTTTACTATTACTATCATTACCAGTACTCGCTGGAGCAATTACTATACTTTTAACTGACCGAAATTTTAA

>Napoli_3_(NA1)_Italy_(present_study)

GTAATTGTTACAGCTCACGCATTTGTTATAATTTTTTTTCTTGTTATACCAGTTATAATTGGAGGATTTGGAAACTGATTAGTTCCTTTAATACTAGGAACACCAGATATAGCATTCCCACGAATAAATAACATAAGCTTCTGACTCTTACCTCCTTCTCTCACTCTTCTCCTTTCATCTGCAGCTGTTGAAAGAGGTGCAGGTACCGGATGAACCGTTTACCCACCTCTTTCAAGAAATTTAGCTCATATAGGACCCTCTGTTGATCTAGCAATTTTCTCACTTCACTTAGCAGGTATTTCATCAATTCTTGGAGCCATCAACTTTATTACAACTATTATTAATATACGATGAGAAGGTATATTAATAGAACGACTTCCACTATTTGTGTGATCTGTTTTTATTACCGCAATTTTACTATTATTATCATTACCAGTACTCGCTGGAGCAATTACTATACTTTTAACTGACCGAAATTTTAA

>Napoli_4_(NA1)_Italy_(present_study)

GTAATTGTTACAGCTCACGCATTTGTTATAATTTTTTTTCTTGTTATACCAGTTATAATTGGAGGATTTGGAAACTGATTAGTTCCTTTAATACTAGGAGCACCAGATATAGCATTCCCACGAATAAATAACATAAGCTTCTGACTCTTACCTCCTTCTCTCACTCTTCTCCTTTCATCTGCAGCTGTTGAAAGTGGTGCAGGTACCGGATGAACCGTTTACCCACCTCTTTCAAGAAATTTAGCTCATATAGGACCCTCTGTTGATCTAGCAATTTTCTCACTTCACTTAGCAGGTATTTCATCAATTCTTGGAGCCATCAATTTTATTACAACTATTATTAATATACGATGAGAAGGTATATTAATAGAACGACTTCCACTATTTGTGTGATCTGTTTTTATTACCGCAATTTTACTATTACTATCATTACCAGTACTCGCTGGAGCAATTACTATACTTTTAACTGACCGAAATTTTAA

>Napoli_5_(NA1)_Italy_(present_study)

GTAATTGTTACAGCTCACGCATTTGTTATAATTTTTTTTCTTGTTATACCAGTTATAATTGGAGGATTTGGAAACTGATTAGTTCCTTTAATACTAGGAGCACCAGATATAGCATTCCCACGAATAAATAACATAAGCTTCTGACTCTTACCTCCTTCTCTCACTCTTCTCCTTTCATCTGCAGCTGTTGAAAGAGGTGCAGGTACCGGATGAACCGTTTACCCACCTCTTTCAAGAAATTTAGCTCATATAGGACCCTCTGTTGATCTAGCAATTTTCTCACTTCACTTAGCAGGTATTTCATCAATTCTTGGAGCCATCAACTTTATTACAACTATTATTAATATACGATGAGAAGGTATATTAATAGAACGACTTCCACTATTTGTGTGATCTGTTTTTATTACCGCAATTTTACTATTATTATCATTACCAGTACTCGCTGGAGCAATTACTATACTTTTAACTGACCGAAATTTTAA

>Napoli_1(NA2)_Italy_(present_study)

GTAATTGTTACAGCTCACGCATTTGTTATAATTTTTTTTCTTGTTATACCAGTTATAATTGGAGGATTTGGAAACTGATTAGTTCCTTTAATACTAGGAGCACCAGATATAGCATTCCCACGAATAAATAACATAAGCTTCTGACTCTTACCTCCTTCTCTCACTCTTCTCCTTTCATCTGCAGCTGTTGAAAGTGGTGCAGGTACCGGATGAACCGTTTACCCACCTCTTTCAAGAAATTTAGCTCATATAGGACCCTCTGTTGATCTAGCAATTTTCTCACTTCACTTAGCAGGTATTTCATCAATTCTTGGAGCCATCAATTTTATTACAACTATTATTAATATACGATGAGAAGGTATATTAATAGAACGACTTCCACTATTTGTGTGATCTGTTTTTATTACCGCAATTTTACTATTACTATCATTACCAGTACTCGCTGGAGCAATTACTATACTTTTAACTGACCGAAATTTTAA

>Napoli_2_(NA2)_Italy_(present_study)

GTAATTGTTACAGCTCACGCATTTGTTATAATTTTTTTTCTTGTTATACCAGTTATAATTGGAGGATTTGGAAACTGATTAGTTCCTTTAATACTAGGAGCACCAGATATAGCATTCCCACGAATAAATAACATAAGCTTCTGACTCTTACCTCCTTCTCTCACTCTTCTCCTTTCATCTGCAGCTGTTGAAAGTGGTGCAGGTACCGGATGAACCGTTTACCCACCACTTTCAAGAAATTTAGCTCATATAGGACCCTCTGTTGATCTAGCAATTTTCTCACTTCACTTAGCAGGTATTTCATCAATTCTTGGAGCCATCAATTTTATTACAACTATTATTAATATACGATGAGAAGGTATATTAATAGAACGACTTCCACTATTTGTGTGATCTGTTTTTATTACCGCAATTTTACTATTACTATCATTACCAGTACTCGCTGGAGCAATTACTATACTTTTAACTGACCGAAATTTTAA

>Napoli_3_(NA2)_Italy_(present_study)

GTAATTGTTACAGCTCACGCATTTGTTATAATTTTTTTTCTTGTTATACCAGTTATAATTGGAGGATTTGGAAACTGATTAGTTCCTTTAATACTAGGAGCACCAGATATAGCATTCCCACGAATAAATAACATAAGCTTCTGACTCTTACCTCCTTCTCTCACTCTTCTCCTTTCATCTGCAGCTGTTGAAAGAGGTGCAGGTACCGGATGAACCGTTTACCCACCTCTTTCAAGAAATTTAGCTCATATAGGACCCTCTGTTGATCTAGCAATTTTCTCACTTCACTTAGCAGGTATTTCATCAATTCTTGGAGCCATCAACTTTATTACAACTATTATTAATATACGATGAGAAGGTATATTAATAGAACGACTTCCACTATTTGTGTGATCTGTTTTTATTACCGCAATTTTACTATTATTATCATTACCAGTACTCGCTGGAGCAATTACTATACTTTTAACTGACCGAAATTTTAA

>Napoli_4_(NA2)_Italy_(present_study)

GTAATTGTTACAGCTCACGCATTTGTTATAATTTTTTTTCTTGTTATACCAGTTATAATTGGAGGATTTGGAAACTGATTAGTTCCTTTAATACTAGGAGCACCAGATATAGCATTCCCACGAATAAATAACATAAGCTTCTGACTCTTACCTCCTTCTCTCACTCTTCTCCTTTCATCTGCAGCTGTTGAAAGAGGTGCAGGTACCGGATGAACCGTTTACCCACCTCTTTCAAGAAATTTAGCTCATATAGGACCCTCTGTTGATCTAGCAATTTTCTCACTTCACTTAGCAGGTATTTCATCAATTCTTGGAGCCATCAACTTTATTACAACTATTATTAATATACGATGAGAAGGTATATTAATAGAACGACTTCCACTATTTGTGTGATCTGTTTTTATTACCGCAATTTTACTATTATTATCATTACCAGTACTCGCTGGAGCAATTACTATACTTTTAACTGACCGAAATTTTAA

>Napoli_5_(NA2)_Italy_(present_study)

GTAATTGTTACAGCTCACGCATTTGTTATAATTTTTTTTCTTGTTATACCAGTTATAATTGGAGGATTTGGAAACTGATTAGTTCCTTTAATACTAGGAGCACCAGATATAGCATTCCCACGAATAAATAACATAAGCTTCTGACTCTTACCTCCTTCTCTCACTCTTCTCCTTTCATCTGCAGCTGTTGAAAGTGGTGCAGGTACCGGATGAACCGTTTACCCACCTCTTTCAAGAAATTTAGCTCATATAGGACCCTCTGTTGATCTAGCAATTTTCTCACTTCACTTAGCAGGTATTTCATCAATTCTTGGAGCCATCAATTTTATTACAACTATTATTAATATACGATGAGAAGGTATATTAATAGAACGACTTCCACTATTTGTGTGATCTGTTTTTATTACCGCAATTTTACTATTACTATCATTACCAGTACTCGCTGGAGCAATTACTATACTTTTAACTGACCGAAATTTTAA

>Porto_Cesareo_1_Italy_(present_study)

GTAATTGTTACAGCTCACGCATTTGTTATAATTTTTTTTCTTGTTATACCAGTTATAATTGGAGGATTTGGAAACTGATTAGTTCCTTTAATACTAGGAGCACCAGATATAGCATTCCCACGAATAAATAACATAAGCTTCTGACTCTTACCTCCTTCTCTCACTCTTCTCCTTTCATCTGCAGCTGTTGAAAGAGGTGCAGGTACCGGATGAACCGTTTACCCACCTCTTTCAAGAAATTTAGCTCATATAGGACCCTCTGTTGATCTAGCAATTTTCTCACTTCACTTAGCAGGTATTTCATCAATTCTTGGAGCCATCAACTTTATTACAACTATTATTAATATACGATGAGAAGGTATATTAATAGAACGACTTCCACTATTTGTGTGATCTGTTTTTATTACCGCAATTTTACTATTATTATCATTACCAGTACTCGCTGGAGCAATTACTATACTTTTAACTGACCGAAATTTTAA

>Porto_Cesareo_2_Italy_(present_study)

GTAATTGTTACAGCTCACGCATTTGTTATAATTTTTTTTCTTGTTATACCAGTTATAATTGGAGGATTTGGAAACTGATTAGTTCCTTTAATACTAGGAGCACCAGATATAGCATTCCCACGAATAAATAACATAAGCTTCTGACTCTTACCTCCTTCTCTCACTCTTCTCCTTTCATCTGCAGCTGTTGAAAGAGGTGCAGGTACCGGATGAACCGTTTACCCACCTCTTTCAAGAAATTTAGCTCATATAGGACCCTCTGTTGATCTAGCAATTTTCTCACTTCACTTAGCAGGTATTTCATCAATTCTTGGAGCCATCAACTTTATTACAACTATTATTAATATACGATGAGAAGGTATATTAATAGAACGACTTCCACTATTTGTGTGATCTGTTTTTATTACCGCAATTTTACTATTATTATCATTACCAGTACTCGCTGGAGCAATTACTATACTTTTAACTGACCGAAATTTTAA

>Porto_Cesareo_3_Italy_(present_study)

GTAATTGTTACAGCTCACGCATTTGTTATAATTTTTTTTCTTGTTATACCAGTTATAATTGGAGGATTTGGAAACTGATTAGTTCCTTTAATACTAGGAGCACCAGATATAGCATTCCCACGAATAAATAACATAAGCTTCTGACTCTTACCTCCTTCTCTCACTCTTCTCCTTTCATCTGCAGCTGTTGAAAGAGGTGCAGGTACCGGATGAACCGTTTACCCACCTCTTTCAAGAAATTTAGCTCATATAGGACCCTCTGTTGATCTAGCAATTTTCTCACTTCACTTAGCAGGTATTTCATCAATTCTTGGAGCCATCAACTTTATTACAACTATTATTAATATACGATGAGAAGGTATATTAATAGAACGACTTCCACTATTTGTGTGATCTGTTTTTATTACCGCAATTTTACTATTATTATCATTACCAGTACTCGCTGGAGCAATTACTATACTTTTAACTGACCGAAATTTTAA

>Porto_Cesareo_4_Italy_(present_study)

GTAATTGTTACAGCTCACGCATTTGTTATAATTTTTTTTCTTGTTATACCAGTTATAATTGGAGGATTTGGAAACTGATTAGTTCCTTTAATACTAGGAGCACCAGATATAGCATTCCCACGAATAAATAACATAAGCTTCTGACTCTTACCTCCTTCTCTCACTCTTCTCCTTTCATCTGCAGCTGTTGAAAGTGGTGCAGGTACCGGATGAACCGTTTACCCACCTCTTTCAAGAAATTTAGCTCATATAGGACCCTCTGTTGATCTAGCAATTTTCTCACTTCACTTAGCAGGTATTTCATCAATTCTTGGAGCCATCAATTTTATTACAACTATTATTAATATACGATGAGAAGGTATATTAATAGAACGACTTCCACTATTTGTGTGATCTGTTTTTATTACCGCAATTTTACTATTACTATCATTACCAGTACTCGCTGGAGCAATTACTATACTTTTAACTGACCGAAATTTTAA

>Porto_Cesareo_5_Italy_(present_study)

GTAATTGTTACAGCTCACGCATTTGTTATAATTTTTTTTCTTGTTATACCAGTTATAATTGGAGGATTTGGAAACTGATTAGTTCCTTTAATACTAGGAGCACCAGATATAGCATTCCCACGAATAAATAACATAAGCTTCTGACTCTTACCTCCTTCTCTCACTCTTCTCCTTTCATCTGCAGCTGTTGAAAGTGGTGCAGGTACCGGATGAACCGTTTACCCACCTCTTTCAAGAAATTTAGCTCATATAGGACCCTCTGTTGATCTAGCAATTTTCTCACTTCACTTAGCAGGTATTTCATCAATTCTTGGAGCCATCAATTTTATTACAACTATTATTAATATACGATGAGAAGGTATATTAATAGAACGACTTCCACTATTTGTGTGATCTGTTTTTATTACCGCAATTTTACTATTACTATCATTACCAGTACTCGCTGGAGCAATTACTATACTTTTAACTGACCGAAATTTTAA

>Croatia_1_(present_study)

GTAATTGTTACAGCTCACGCATTTGTTATAATTTTTTTTCTTGTTATACCAGTTATAATTGGAGGATTTGGAAACTGATTAGTTCCTTTAATACTAGGAGCACCAGATATAGCATTCCCACGAATAAATAACATAAGCTTCTGACTCTTACCTCCTTCTCTCACTCTTCTCCTTTCATCTGCAGCTGTTGAAAGAGGTGCAGGTACCGGATGAACCGTTTACCCACCTCTTTCAAGAAATTTAGCTCATATAGGACCCTCTGTTGATCTAGCAATTTTCTCACTTCACTTAGCAGGTATTTCATCAATTCTTGGAGCCATCAACTTTATTACAACTATTATTAATATACGATGAGAAGGTATATTAATAGAACGACTTCCACTATTTGTGTGATCTGTTTTTATTACCGCAATTTTACTATTATTATCATTACCAGTACTCGCTGGAGCAATTACTATACTTTTAACTGACCGAAATTTTAA

>Croatia_2_(present_study)

GTAATTGTTACAGCTCACGCATTTGTTATAATTTTTTTTCTTGTTATACCAGTTATAATTGGAGGATTTGGAAACTGATTAGTTCCTTTAATACTAGGAGCACCAGATATAGCATTCCCACGAATAAATAACATAAGCTTCTGACTCTTACCTCCTTCTCTCACTCTTCTCCTTTCATCTGCAGCTGTTGAAAGAGGTGCAGGTACCGGATGAACCGTTTACCCACCTCTTTCAAGAAATTTAGCTCATATAGGACCCTCTGTTGATCTAGCAATTTTCTCACTTCACTTAGCAGGTATTTCATCAATTCTTGGAGCCATCAACTTTATTACAACTATTATTAATATACGATGAGAAGGTATATTAATAGAACGACTTCCACTATTTGTGTGATCTGTTTTTATTACCGCAATTTTACTATTATTATCATTACCAGTACTCGCTGGAGCAATTACTATACTTTTAACTGACCGAAATTTTAA

>Croatia_3_(present_study)

GTAATTGTTACAGCTCACGCATTTGTTATAATTTTTTTTCTTGTTATACCAGTTATAATTGGAGGATTTGGAAACTGATTAGTTCCTTTAATACTAGGAGCACCAGATATAGCATTCCCACGAATAAATAACATAAGCTTCTGACTCTTACCTCCTTCTCTCACTCTTCTCCTTTCATCTGCAGCTGTTGAAAGAGGTGCAGGTACCGGATGAACCGTTTACCCACCTCTTTCAAGAAATTTAGCTCATATAGGACCCTCTGTTGATCTAGCAATTTTCTCACTTCACTTAGCAGGTATTTCATCAATTCTTGGAGCCATCAACTTTATTACAACTATTATTAATATACGATGAGAAGGTATATTAATAGAACGACTTCCACTATTTGTGTGATCTGTTTTTATTACCGCAATTTTACTATTATTATCATTACCAGTACTCGCTGGAGCAATTACTATACTTTTAACTGACCGAAATTTTAA

>Croatia_4_(present_study)

GTAATTGTTACAGCTCACGCATTTGTTATAATTTTTTTTCTTGTTATACCAGTTATAATTGGAGGATTTGGAAACTGATTAGTTCCTTTAATACTAGGAGCACCAGATATAGCATTCCCACGAATAAATAACATAAGCTTCTGACTCTTACCTCCTTCTCTCACTCTTCTCCTTTCATCTGCAGCTGTTGAAAGAGGTGCAGGTACCGGATGAACCGTTTACCCACCTCTTTCAAGAAATTTAGCTCATATAGGACCCTCTGTTGATCTAGCAATTTTCTCACTTCACTTAGCAGGTATTTCATCAATTCTTGGAGCCATCAACTTTATTACAACTATTATTAATATACGATGAGAAGGTATATTAATAGAACGACTTCCACTATTTGTGTGATCTGTTTTTATTACCGCAATTTTACTATTATTATCATTACCAGTACTCGCTGGAGCAATTACTATACTTTTAACTGACCGAAATTTTAA

>Croatia_5_(present_study)

GTAATTGTTACAGCTCACGCATTTGTTATAATTTTTTTTCTTGTTATACCAGTTATAATTGGAGGATTTGGAAACTGATTAGTTCCTTTAATACTAGGAGCACCAGATATAGCATTCCCACGAATAAATAACATAAGCTTCTGACTCTTACCTCCTTCTCTCACTCTTCTCCTTTCATCTGCAGCTGTTGAAAGAGGTGCAGGTACCGGATGAACCGTTTACCCACCTCTTTCAAGAAATTTAGCTCATATAGGACCCTCTGTTGATCTAGCAATTTTCTCACTTCACTTAGCAGGTATTTCATCAATTCTTGGAGCCATCAACTTTATTACAACTATTATTAATATACGATGAGAAGGTATATTAATAGAACGACTTCCACTATTTGTGTGATCTGTTTTTATTACCGCAATTTTACTATTATTATCATTACCAGTACTCGCTGGAGCAATTACTATACTTTTAACTGACCGAAATTTTAA

>Spain_1_(present_study)

GTAATTGTTACAGCTCACGCATTTGTTATAATTTTTTTTCTTGTTATACCAGTTATAATTGGAGGATTTGGAAACTGATTAGTTCCTTTAATACTAGGAGCACCAGATATAGCATTCCCACGAATAAATAACATAAGCTTCTGACTCTTACCTCCTTCTCTCACTCTTCTCCTTTCATCTGCAGCTGTTGAAAGAGGTGCAGGTACCGGATGAACCGTTTACCCACCTCTTTCAAGAAATTTAGCTCATATAGGACCCTCTGTTGATCTAGCAATTTTCTCACTTCACTTAGCAGGTATTTCATCAATTCTTGGAGCCATCAACTTTATTACAACTATTATTAATATACGATGAGAAGGTATATTAATAGAACGACTTCCACTATTTGTGTGATCTGTTTTTATTACCGCAATTTTACTATTATTATCATTACCAGTACTCGCTGGAGCAATTACTATACTTTTAACTGACCGAAATTTTAA

>Spain_2_(present_study)

GTAATTGTTACAGCTCACGCATTTGTTATAATTTTTTTTCTTGTTATACCAGTTATAATTGGAGGATTTGGAAACTGATTAGTTCCTTTAATACTAGGAGCACCAGATATAGCATTCCCACGAATAAATAACATAAGCTTCTGACTCTTACCTCCTTCTCTCACTCTTCTCCTTTCATCTGCAGCTGTTGAAAGTGGTGCAGGTACCGGATGAACCGTTTACCCACCTCTTTCAAGAAATTTAGCTCATATAGGACCCTCTGTTGATCTAGCAATTTTCTCACTTCACTTAGCAGGTATTTCATCAATTCTTGGACCCATCAATTTTATTACAACTATTATTAATATACGATGAGAAGGTATATTAATAGAACGACTTCCACTATTTGTGTGATCTGTTTTTATTACCGCAATTTTACTATTACTATCATTACCAGTACTCGCTGGAGCAATTACTATACTTTTAACTGACCGAAATTTTAA

>Spain_3_(present_study)

GTAATTGTTACAGCTCACGCATTTGTTATAATTTTTTTTCTTGTTATACCAGTTATAATTGGAGGATTTGGAAACTGATTAGTTCCTTTAATACTAGGAGCACCAGATATAGCATTCCCACGAATAAATAACATAAGCTTCTGACTCTTACCTCCTTCTCTCACTCTTCTCCTTTCATCTGCAGCTGTTGAAAGTGGTGCAGGTACCGGATGAACCGTTTACCCACCTCTTTCAAGAAATTTAGCTCATATAGGACCCTCTGTTGATCTAGCAATTTTCTCACTTCACTTAGCAGGTATTTCATCAATTCTTGGAGCCATCAATTTTATTACAACTATTATTAATATACGATGAGAAGGTATATTAATAGAACGACTTCCACTATTTGTGTGATCTGTTTTTATTACCGCAATTTTACTATTACTATCATTACCAGTACTCGCTGGAGCAATTACTATACTTTTAACTGACCGAAATTTTAA

>Spain_4_(present_study)

GTAATTGTTACAGCTCACGCATTTGTTATAATTTTTTTTCTTGTTATACCAGTTATAATTGGAGGATTTGGAAACTGATTAGTTCCTTTAATACTAGGAGCACCAGATATAGCATTCCCACGAATAAATAACATAAGCTTCTGACTCTTACCTCCTTCTCTCACTCTTCTCCTTTCATCTGCAGCTGTTGAAAGAGGTGCAGGTACCGGATGAACCGTTTACCCACCTCTTTCAAGAAATTTAGCTCATATAGGACCCTCTGTTGATCTAGCAATTTTCTCACTTCACTTAGCAGGTATTTCATCAATTCTTGGAGCCATCAACTTTATTACAACTATTATTAATATACGATGAGAAGGTATATTAATAGAACGACTTCCACTATTTGTGTGATCTGTTTTTATTACCGCAATTTTACTATTATTATCATTACCAGTACTCGCTGGAGCAATTACTATACTTTTAACTGACCGAAATTTTAA

>Spain_5_(present_study)

GTAATTGTTACAGCTCACGCATTTGTTATAATTTTTTTTCTTGTTATACCAGTTATAATTGGAGGATTTGGAAACTGATTAGTTCCTTTAATACTAGGAGCACCAGATATAGCATTCCCACGAATAAATAACATAAGCTTCTGACTCTTACCTCCTTCTCTCACTCTTCTCCTTTCATCTGCAGCTGTTGAAAGTGGTGCAGGTACCGGATGAACCGTTTACCCACCTCTTTC?AGAAATTTAGCTCATATAGGACCCTCTGTTGATCTAGCAATTTTCTCACTTCACTTAGCAGGTATTTCATCAATTCTTGGAGCCATCAATTTTATTACAACTATTATTAATATACGATGAGAAGGTATATTAATAGAACGACTTCCACTATTTGTGTGATCTGTTTTTATTACCGCAATTTTACTATTACTATCATTACCAGTACTCGCTGGAGCAATTACTATACTTTTAACTGACCGAAATTTTAA

>Oristano_1_Italy_(present_study)

GTAATTGTTACAGCTCACGCATTTGTTATAATTTTTTTTCTTGTTATACCAGTTATAATTGGAGGATTTGGAAACTGATTAGTTCCTTTAATACTAGGAGCACCAGATATAGCATTCCCACGAATAAATAACATAAGCTTCTGACTCTTACCTCCTTCTCTCACTCTTCTCCTTTCATCTGCAGCTGTTGAAAGAGGTGCAGGTACCGGATGAACCGTTTACCCACCTCTTTCAAGAAATTTAGCTCATATAGGACCCTCTGTTGATCTAGCAATTTTCTCACTTCACTTAGCAGGTATTTCATCAATTCTTGGAGCCATCAACTTTATTACAACTATTATTAATATACGATGAGAAGGTATATTAATAGAACGACTTCCACTATTTGTGTGATCTGTTTTTATTACCGCAATTTTACTATTATTATCATTACCAGTACTCGCTGGAGCAATTACTATACTTTTAACTGACCGAAATTTTAA

>Oristano_2_Italy_(present_study)

GTAATTGTTACAGCTCACGCATTTGTTATAATTTTTTTTCTTGTTATACCAGTTATAATTGGAGGATTTGGAAACTGATTAGTTCCTTTAATACTAGGAGCACCAGATATAGCATTCCCACGAATAAATAACATAAGCTTCTGACTCTTACCTCCTTCTCTCACTCTTCTCCTTTCATCTGCAGCTGTTGAAAGAGGTGCAGGTACCGGATGAACCGTTTACCCACCTCTTTCAAGAAATTTAGCTCATATAGGACCCTCTGTTGATCTAGCAATTTTCTCACTTCACTTAGCAGGTATTTCATCAATTCTTGGAGCCATCAACTTTATTACAACTATTATTAATATACGATGAGAAGGTATATTAATAGAACGACTTCCACTATTTGTGTGATCTGTTTTTATTACCGCAATTTTACTATTATTATCATTACCAGTACTCGCTGGAGCAATTACTATACTTTTAACTGACCGAAATTTTAA

>Oristano_3_Italy_(present_study)

GTAATTGTTACAGCTCACGCATTTGTTATAATTTTTTTTCTTGTTATACCAGTTATAATTGGAGGATTTGGAAACTGATTAGTTCCTTTAATACTAGGAGCACCAGATATAGCATTCCCACGAATAAATAACATAAGCTTCTGACTCTTACCTCCTTCTCTCACTCTTCTCCTTTCATCTGCAGCTGTTGAAAGAGGTGCAGGTACCGGATGAACCGTTTACCCACCTCTTTCAAGAAATTTAGCTCATATAGGACCCTCTGTTGATCTAGCAATTTTCTCACTTCACTTAGCAGGTATTTCATCAATTCTTGGAGCCATCAACTTTATTACAACTATTATTAATATACGATGAGAAGGTATATTAATAGAACGACTTCCACTATTTGTGTGATCTGTTTTTATTACCGCAATTTTACTATTATTATCATTACCAGTACTCGCTGGAGCAATTACTATACTTTTAACTGACCGAAATTTTAA

>Oristano_4_Italy_(present_study)

GTAATTGTTACAGCTCACGCATTTGTTATAATTTTTTTTCTTGTTATACCAGTTATAATTGGAGGATTTGGAAACTGATTAGTTCCTTTAATACTAGGAGCACCAGATATAGCATTCCCACGAATAAATAACATAAGCTTCTGACTCTTACCTCCTTCTCTCACTCTTCTCCTTTCATCTGCAGCTGTTGAAAGTGGTGCAGGTACCGGATGAACCGTTTACCCACCTCTTTCAAGAAATTTAGCTCATATAGGACCCTCTGTTGATCTAGCAATTTTCTCACTTCACTTAGCAGGTATTTCATCAATTCTTGGAGCCATCAATTTTATTACAACTATTATTAATATACGATGAGAAGGTATATTAATAGAACGACTTCCACTATTTGTGTGATCTGTTTTTATTACCGCAATTTTACTATTACTATCATTACCAGTACTCGCTGGAGCAATTACTATACTTTTAACTGACCGAAATTTTAA

>Oristano_5_(present_study)

GTAATTGTTACAGCTCACGCATTTGTTATAATTTTTTTTCTTGTTATACCAGTTATAATTGGAGGATTTGGAAACTGATTAGTTCCTTTAATACTAGGAGCACCAGATATAGCATTCCCACGAATAAATAACATAAGCTTCTGACTCTTACCTCCTTCTCTCACTCTTCTCCTTTCATCTGCAGCTGTTGAAAGAGGTGCAGGTACCGGATGAACCGTTTACCCACCTCTTTCAAGAAATTTAGCTCATATAGGACCCTCTGTTGATCTAGCAATTTTCTCACTTCACTTAGCAGGTATTTCATCAATTCTTGGAGCCATCAACTTTATTACAACTATTATTAATATACGATGAGAAGGTATATTAATAGAACGACTTCCACTATTTGTGTGATCTGTTTTTATTACCGCAATTTTACTATTATTATCATTACCAGTACTCGCTGGAGCAATTACTATACTTTTAACTGACCGAAATTTTAA

>Ria_Formosa_1_Portugal_(present_study)

GTAATTGTTACAGCTCACGCATTTGTTATAATTTTTTTTCTTGTTATACCAGTTATAATTGGAGGATTTGGAAACTGATTAGTTCCTTTAATACTAGGAGCACCAGATATAGCATTCCCACGAATAAATAACATAAGCTTCTGACTCTTACCTCCTTCTCTCACTCTTCTCCTTTCATCTGCAGCTGTTGAAAGTGGTGCAGGTACCGGATGAACCGTTTACCCACCTCTTTCAAGAAATTTAGCTCATATAGGACCCTCTGTTGATCTAGCAATTTTCTCACTTCACTTAGCAGGTATTTCATCAATTCTTGGAGCCATCAATTTTATTACAACTATTATTAATATACGATGAGAAGGTATATTAATAGAACGACTTCCACTATTTGTGTGATCTGTTTTTATTACCGCAATTTTACTATTACTATCATTACCAGTACTCGCTGGAGCAATTACTATACTTTTAACTGACCGAAATTTTAA

>Ria_Formosa_2_Portugal_(present_study)

GTAATTGTTACAGCTCACGCATTTGTTATAATTTTTTTTCTTGTTATACCAGTTATAATTGGAGGATTTGGAAACTGATTAGTTCCTTTAATACTAGGAGCACCAGATATAGCATTCCCACGAATAAATAACATAAGCTTCTGACTCTTACCTCCTTCTCTCACTCTTCTCCTTTCATCTGCAGCTGTTGAAAGTGGTGCAGGTACCGGATGAACCGTTTACCCACCTCTTTCAAGAAATTTAGCTCATATAGGACCCTCTGTTGATCTAGCAATTTTCTCACTTCACTTAGCAGGTATTTCATCAATTCTTGGAGCCATCAATTTTATTACAACTATTATTAATATACGATGAGAAGGTATATTAATAGAACGACTTCCACTATTTGTGTGATCTGTTTTTATTACCGCAATTTTACTATTACTATCATTACCAGTACTCGCTGGAGCAATTACTATACTTTTAACTGACCGAAATTTTAA

>Ria_Formosa_3_Portugal_(present_study)

GTAATTGTTACAGCTCACGCATTTGTTATAATTTTTTTTCTTGTTATACCAGTTATAATTGGAGGATTTGGAAACTGATTAGTTCCTTTAATACTAGGAGCACCAGATATAGCATTCCCACGAATAAATAACATAAGCTTCTGACTCTTACCTCCTTCTCTCACTCTTCTCCTTTCATCTGCAGCTGTTGAAAGTGGTGCAGGTACCGGATGAACCGTTTACCCACCTCTTTCAAGAAATTTAGCTCATATAGGACCCTCTGTTGATCTAGCAATTTTCTCACTTCACTTAGCAGGTATTTCATCAATTCTTGGAGCCATCAATTTTATTACAACTATTATTAATATACGATGAGAAGGTATATTAATAGAACGACTTCCACTATTTGTGTGATCTGTTTTTATTACCGCAATTTTACTATTACTATCATTACCAGTACTCGCTGGAGCAATTACTATACTTTTAACTGACCGAAATTTTAA

>Strait_of_Messina_1_Italy_(present_study)

GTAATTGTTACAGCTCACGCATTTGTTATAATTTTTTTTCTTGTTATACCAGTTATAATTGGAGGATTTGGAAACTGATTAGTTCCTTTAATACTAGGAGCACCAGATATAGCATTCCCACGAATAAATAACATAAGCTTCTGACTCTTACCTCCTTCTCTCACTCTTCTCCTTTCATCTGCAGCTGTTGAAAGAGGTGCAGGTACCGGATGAACCGTTTACCCACCTCTTTCAAGAAATTTAGCTCATATAGGACCCTCTGTTGATCTAGCAATTTTCTCACTTCACTTAGCAGGTATTTCATCAATTCTTGGAGCCATCAACTTTATTACAACTATTATTAATATACGATGAGAAGGTATATTAATAGAACGACTTCCACTATTTGTGTGATCTGTTTTTATTACCGCAATTTTACTATTATTATCATTACCAGTACTCGCTGGAGCAATTACTATACTTTTAACTGACCGAAATTTTAA

>Strait_of_Messina_2_Italy_(present_study)

GTAATTGTTACAGCTCACGCATTTGTTATAATTTTTTTTCTTGTTATACCAGTTATAATTGGAGGATTTGGAAACTGATTAGTTCCTTTAATACTAGGAGCACCAGATATAGCATTCCCACGAATAAATAACATAAGCTTCTGACTCTTACCTCCTTCTCTCACTCTTCTCCTTTCATCTGCAGCTGTTGAAAGAGGTGCAGGTACCGGATGAACCGTTTACCCACCTCTTTCAAGAAATTTAGCTCATATAGGACCCTCTGTTGATCTAGCAATTTTCTCACTTCACTTAGCAGGTATTTCATCAATTCTTGGAGCCATCAACTTTATTACAACTATTATTAATATACGATGAGAAGGTATATTAATAGAACGACTTCCACTATTTGTGTGATCTGTTTTTATTACCGCAATTTTACTATTATTATCATTACCAGTACTCGCTGGAGCAATTACTATACTTTTAACTGACCGAAATTTTAA

>Strait_of_Messina_3_Italy_(present_study)

GTAATTGTTACAGCTCACGCATTTGTTATAATTTTTTTTCTTGTTATACCAGTTATAATTGGAGGATTTGGAAACTGATTAGTTCCTTTAATACTAGGAGCACCAGATATAGCATTCCCACGAATAAATAACATAAGCTTCTGACTCTTACCTCCTTCTCTCACTCTTCTCCTTTCATCTGCAGCTGTTGAAAGAGGTGCAGGTACCGGATGAACCGTTTACCCACCTCTTTCAAGAAATTTAGCTCATATAGGACCCTCTGTTGATCTAGCAATTTTCTCACTTCACTTAGCAGGTATTTCATCAATTCTTGGAGCCATCAACTTTATTACAACTATTATTAATATACGATGAGAAGGTATATTAATAGAACGACTTCCACTATTTGTGTGATCTGTTTTTATTACCGCAATTTTACTATTATTATCATTACCAGTACTCGCTGGAGCAATTACTATACTTTTAACTGACCGAAATTTTAA

>Strait_of_Messina_4_Italy_(present_study)

GTAATTGTTACAGCTCACGCATTTGTTATAATTTTTTTTCTTGTTATACCAGTTATAATTGGAGGATTTGGAAACTGATTAGTTCCTTTAATACTAGGAGCACCAGATATAGCATTCCCACGAATAAATAACATAAGCTTCTGACTCTTACCTCCTTCTCTCACTCTTCTCCTTTCATCTGCAGCTGTTGAAAGAGGTGCAGGTACCGGATGAACCGTTTACCCACCTCTTTCAAGAAATTTAGCTCATATAGGACCCTCTGTTGATCTAGCAATTTTCTCACTTCACTTAGCAGGTATTTCATCAATTCTTGGAGCCATCAACTTTATTACAACTATTATTAATATACGATGAGAAGGTATATTAATAGAACGACTTCCACTATTTGTGTGATCTGTTTTTATTACCGCAATTTTACTATTATTATCATTACCAGTACTCGCTGGAGCAATTACTATACTTTTAACTGACCGAAATTTTAA

>Strait_of_Messina_5_Italy_(present_study)

GTAATTGTTACAGCTCACGCATTTGTTATAATTTTTTTTCTTGTTATACCAGTTATAATTGGAGGATTTGGAAACTGATTAGTTCCTTTAATACTAGGAGCACCAGATATAGCATTCCCACGAATAAATAACATAAGCTTCTGACTCTTACCTCCTTCTCTCACTCTTCTCCTTTCATCTGCAGCTGTTGAAAGAGGTGCAGGTACCGGATGAACCGTTTACCCACCTCTTTCAAGAAATTTAGCTCATATAGGACCCTCTGTTGATCTAGCAATTTTCTCACTTCACTTAGCAGGTATTTCATCAATTCTTGGAGCCATCAACTTTATTACAACTATTATTAATATACGATGAGAAGGTATATTAATAGAACGACTTCCACTATTTGTGTGATCTGTTTTTATTACCGCAATTTTACTATTATTATCATTACCAGTACTCGCTGGAGCAATTACTATACTTTTAACTGACCGAAATTTTAA

>Japan_(AB052253)

GTAATTGTTACAGCTCACGCATTTGTTATAATTTTTTTCCTTGTTATACCAGTTATAATCGGAGGATTTGGAAACTGATTAGTTCCTTTAATACTAGGAGCACCAGATATAGCATTCCCACGAATAAATAATATAAGCTTCTGACTCTTACCTCCTTCTCTTACTCTTCTCCTTTCATCTGCAGCAGTTGAAAGAGGCGCAGGTACCGGATGAACCGTTTACCCGCCTCTTTCAAGAAATTTAGCTCATATAGGACCTTCTGTTGATCTAGCCATTTTCTCACTTCACTTAGCAGGTATCTCATCAATCCTTGGAGCCATCAACTTTATTACAACTATTATTAATATACGATGAGAAGGTATATTAATAGAACGACTTCCACTATTTGTATGATCTGTATTTATTACCGCAATTTTACTATTACTATCATTACCAGTACTCGCTGGAGCAATTACTATACTTTTAACTGACCGAAATTTTAA

>Tristan_da_Cunha_Southern_Atlantic_Ocean_(DQ683205)

GTAATTGTTACAGCTCACGCATTTGTTATAATTTTTTTTCTTGTTATACCAGTTATAATTGGAGGATTTGGAAACTGATTAGTTCCTTTAATACTAGGAGCACCAGATATAGCATTCCCACGAATAAATAACATAAGCTTCTGACTCTTACCTCCTTCTCTCACTCTTCTCCTTTCATCTGCAGCTGTTGAAAGAGGTGCAGGTACCGGATGAACCGTTTACCCACCTCTTTCAAGAAATTTAGCTCATATAGGACCCTCTGTTGATCTAGCAATTTTCTCACTTCACTTAGCAGGTATTTCATCAATTCTTGGAGCCATCAACTTTATTACAACTATTATTAATATACGATGAGAAGGTATATTAATAGAACGACTTCCACTATTTGTGTGATCTGTTTTTATTACCGCAATTTTACTATTATTATCATTACCAGTACTCGCTGGAGCAATTACTATACTTTTAACTGACCGAAATTTTAA

>Tristan_da_Cunha_Southern_Atlantic_Ocean_(DQ683206)

GTAATTGTTACAGCTCACGCATTTGTTATAATTTTTTTTCTTGTTATACCAGTTATAATTGGAGGATTTGGAAACTGATTAGTTCCTTTAATACTAGGAGCACCAGATATAGCATTCCCACGAATAAATAACATAAGCTTCTGACTCTTACCTCCTTCTCTCACTCTTCTCCTTTCATCTGCAGCTGTTGAAAGAGGTGCAGGTACCGGATGAACCGTTTACCCACCTCTTTCAAGAAATTTAGCTCATATAGGACCCTCTGTTGATCTAGCAATTTTCTCACTTCACTTAGCAGGTATTTCATCAATTCTTGGAGCCATCAACTTTATTACAACTATTATTAATATACGATGAGAAGGTATATTAATAGAACGACTTCCACTATTTGTGTGATCTGTTTTTATTACCGCAATTTTACTATTATTATCATTACCAGTACTCGCTGGAGCAATTACTATACTTTTAACTGACCGAAATTTTAA

>Tristan_da_Cunha_Southern_Atlantic_Ocean_(DQ683207)

GTAATTGTTACAGCTCACGCATTTGTTATAATTTTTTTTCTTGTTATACCAGTTATAATTGGAGGATTTGGAAACTGATTAGTTCCTTTAATACTAGGAGCACCAGATATAGCATTCCCACGAATAAATAACATAAGCTTCTGACTCTTACCTCCTTCTCTCACTCTTCTCCTTTCATCTGCAGCTGTTGAAAGAGGTGCAGGTACCGGATGAACCGTTTACCCACCTCTTTCAAGAAATTTAGCTCATATAGGACCCTCTGTTGATCTAGCAATTTTCTCACTTCACTTAGCAGGTATTTCATCAATTCTTGGAGCCATCAACTTTATTACAACTATTATTAATATACGATGAGAAGGTATATTAATAGAACGACTTCCACTATTTGTGTGATCTGTTTTTATTACCGCAATTTTACTATTATTATCATTACCAGTACTCGCTGGAGCAATTACTATACTTTTAACTGACCGAAATTTTAA

>Hout_Bay_South_Africa_(DQ683208)

GTAATTGTTACAGCTCACGCATTTGTTATAATTTTTTTTCTTGTTATACCAGTTATAATTGGAGGATTTGGAAACTGATTAGTTCCTTTAATACTAGGAGCACCAGATATAGCATTCCCACGAATAAATAACATAAGCTTCTGACTCTTACCTCCTTCTCTCACTCTTCTCCTTTCATCTGCAGCTGTTGAAAGAGGTGCAGGTACCGGATGAACCGTTTACCCACCTCTTTCAAGAAATTTAGCTCATATAGGACCCTCTGTTGATCTAGCAATTTTCTCACTTCACTTAGCAGGTATTTCATCAATTCTTGGAGCCATCAACTTTATTACAACTATTATTAATATACGATGAGAAGGTATATTAATAGAACGACTTCCACTATTTGTGTGATCTGTTTTTATTACCGCAATTTTACTATTATTATCATTACCAGTACTCGCTGGAGCAATTACTATACTTTTAACTGACCGAAATTTTAA

>Hout_Bay_South_Africa_(DQ683209)

GTAATTGTTACAGCTCACGCATTTGTTATAATTTTTTTTCTTGTTATACCAGTTATAATTGGAGGATTTGGAAACTGATTAGTTCCTTTAATACTAGGAGCACCAGATATAGCATTCCCACGAATAAATAACATAAGCTTCTGACTCTTACCTCCTTCTCTCACTCTTCTCCTTTCATCTGCAGCTGTTGAAAGAGGTGCAGGTACCGGATGAACCGTTTACCCACCTCTTTCAAGAAATTTAGCTCATATAGGACCCTCTGTTGATCTAGCAATTTTCTCACTTCACTTAGCAGGTATTTCATCAATTCTTGGAGCCATCAACTTTATTACAACTATTATTAATATACGATGAGAAGGTATATTAATAGAACGACTTCCACTATTTGTATGATCTGTTTTTATTACCGCAATTTTACTATTATTATCATTACCAGTACTCGCTGGAGCAATTACTATACTTTTAACTGACCGAAATTTTAA

>Struisbaai_South_Africa_(DQ683210)

GTAATTGTTACAGCTCACGCATTTGTTATAATTTTTTTTCTTGTTATACCAGTTATAATTGGAGGATTTGGAAACTGATTAGTTCCTTTAATACTAGGAGCACCAGATATAGCATTCCCACGAATAAATAACATAAGCTTCTGACTCTTACCTCCTTCTCTCACTCTTCTCCTTTCATCTGCAGCTGTTGAAAGAGGTGCAGGTACCGGATGAACCGTTTACCCACCTCTTTCAAGAAATTTAGCTCATATAGGACCCTCTGTTGATCTAGCAATTTTCTCACTTCACTTAGCAGGTATTTCATCAATTCTTGGAGCCATCAACTTTATTACAACTATTATTAATATACGATGAGAAGGTATATTAATAGAACGACTTCCACTATTTGTGTGATCTGTTTTTATTACCGCAATTTTACTATTATTATCATTACCAGTACTCGCTGGAGCAATTACTATACTTTTAACTGACCGAAATTTTAA

>Struisbaai_South_Africa_(DQ683211)

GTAATTGTTACAGCTCACGCATTTGTTATAATTTTTTTTCTTGTTATACCAGTTATAATTGGAGGATTTGGAAACTGATTAGTTCCTTTAATACTAAGAGCACCAGATATAGCATTCCCACGAATAAATAACATAAGCTTCTGACTCTTACCTCCTTCTCTCACTCTTCTCCTTTCATCTGCAGCTGTTGAAAGAGGTGCAGGTACCGGATGAACCGTTTACCCACCTCTTTCAAGAAATTTAGCTCATATAGGACCCTCTGTTGATCTAGCAATTTTCTCACTTCACTTAGCAGGTATTTCATCAATTCTTGGAGCCATCAACTTTATTACAACTATTATTAATATACGATGAGAAGGTATATTAATAGAACGACTTCCACTATTTGTGTGATCTGTTTTTATTACCGCAATTTTACTATTATTATCATTACCAGTACTCGCTGGAGCAATTACTATACTTTTAACTGACCGAAATTTTAA

>Port_Elizabeth_South_Africa_(DQ683212)

GTAATTGTTACAGCTCACGCATTTGTTATAATTTTTTTTCTTGTTATACCAGTTATAATTGGAGGATTTGGAAACTGATTAGTTCCTTTAATACTAGGAGCACCAGATATAGCATTCCCACGAATAAATAACATAAGCTTCTGACTCTTACCTCCTTCTCTCACTCTTCTCCTTTCATCTGCAGCTGTTGAAAGAGGTGCAGGTACCGGATGAACCGTTTACCCACCTCTTTCAAGAAATTTAGCTCATATAGGACCCTCTGTTGATCTAGCAATTTTCTCACTTCACTTAGCAGGTATTTCATCAATTCTTGGAGCCATCAACTTTATTACAACTATTATTAATATACGATGAGAAGGTATATTAATAGAACGACTTCCACTATTTGTGTGATCTGTTTTTATTACCGCAATTTTACTATTATTATCATTACCAGTACTCGCTGGAGCAATTACTATACTTTTAACTGACCGAAATTTTAA

>Port_Elizabeth_South_Africa_(DQ683213)

GTAATTGTTACAGCTCACGCATTTGTTATAATTTTTTTTCTTGTTATACCAGTTATAATTGGAGGATTTGGAAACTGATTAGTTCCTTTAATACTAGGAGCACCAGATATAGCATTCCCACGAATAAATAACATAAGCTTCTGACTCTTACCTCCTTCTCTCACTCTTCTCCTTTCATCTGCAGCTGTTGAAAGAGGTGCAGGTACCGGATGAACCGTTTACCCACCTCTTTCAAGAAATTTAGCTCATATAGGACCCTCTGTTGATCTAGCAATTTTCTCACTTCACTTAGCAGGTATTTCATCAATTCTTGGAGCCATCAACTTTATTACAACTATTATTAATATACGATGAGAAGGTATATTAATAGAACGACTTCCACTATTTGTGTGATCTGTTTTTATTACCGCAATTTTACTATTATTATCATTACCAGTACTCGCTGGAGCAATTACTATACTTTTAACTGACCGAAATTTTAA

>Durban_South_Africa_(DQ683214)

GTAATTGTTACAGCTCACGCATTTGTTATAATTTTTTTTCTTGTTATACCAGTTATAATTGGAGGATTTGGAAACTGATTAGTTCCTTTAATACTAGGAGCACCAGATATAGCATTCCCACGAATAAATAACATAAGCTTCTGACTATTACCTCCTTCTCTCACTCTTCTCCTTTCATCTGCAGCTGTTGAAAGAGGTGCAGGTACCGGATGAACCGTTTACCCACCTCTTTCAAGAAATTTAGCTCATATAGGACCCTCTGTTGATCTAGCAATTTTCTCACTTCACTTAGCAGGTATTTCATCAATTCTTGGAGCCATCAACTTTATTACAACTATTATTAATATACGATGAGAAGGTATATTAATAGAACGACTCCCACTATTTGTATGATCTGTTTTTATTACCGCAATTTTACTATTACTATCATTACCAGTACTCGCTGGGGCAATTACTATACTTTTAACTGACCGAAATTTTAA

>Durban_South_Africa_(DQ683215)

GTAATTGTTACAGCTCACGCATTTGTTATAATTTTTTTTCTTGTTATACCAGTTATAATTGGAGGATTTGGAAACTGATTAGTTCCTTTAATACTAGGAGCACCAGATATAGCATTCCCACGAATAAATAACATAAGCTTCTGACTCTTACCTCCTTCTCTCACTCTTCTCCTTTCATCTGCAGCTGTTGAAAGAGGTGCAGGTACCGGATGAACCGTTTACCCACCTCTTTCAAGAAATTTAGCTCATATAGGACCCTCTGTTGATCTAGCAATTTTCTCACTTCACTTAGCAGGTATTTCATCAATTCTTGGAGCCATCAACTTTATTACAACTATTATTAATATACGATGAGAAGGTATATTAATAGAACGACTTCCACTATTTGTGTGATCTGTTTTTATTACCGCAATTTTACTATTATTATCATTACCAGTACTCGCTGGAGCAATTACTATACTTTTAACTGACCGAAATTTTAA

>Durban_South_Africa_(DQ683216)

GTAATTGTTACAGCTCACGCATTTGTTATAATTTTTTTTCTTGTTATACCAGTTATAATTGGAGGATTTGGAAACTGATTAGTTCCTTTAATACTAGGAGCACCAGATATAGCATTCCCACGAATAAATAACATAAGCTTCTGACTATTACCTCCTTCTCTCACTCTTCTCCTTTCATCTGCAGCTGTTGAAAGAGGTGCAGGTACCGGATGAACCGTTTACCCACCTCTTTCAAGAAATTTAGCTCATATAGGACCCTCTGTTGATTTAGCAATTTTCTCACTTCACTTAGCAGGTATTTCATCAATTCTTGGAGCCATCAACTTTATTACAACTATTATTAATATACGATGAGAAGGTATATTAATAGAACGACTCCCACTATTTGTATGATCTGTTTTTATTACCGCAATTTTACTATTACTATCATTACCAGTACTCGCTGGGGCAATTACTATACTTTTAACTGACCGAAATTTTAA

>Durban_South_Africa_(DQ683217)

GTAATTGTTACAGCTCACGCATTTGTTATAATTTTTTTTCTTGTTATACCAGTTATAATTGGAGGATTTGGAAACTGATTAGTTCCTTTAATACTAGGAGCACCAGATATAGCATTCCCACGAATAAATAACATAAGCTTCTGACTCTTACCTCCTTCTCTCACTCTTCTCCTTTCATCTGCAGCTGTTGAAAGAGGTGCAGGTACCGGATGAACCGTTTACCCACCTCTTTCAAGAAATTTAGCTCATATAGGACCCTCTGTTGATCTAGCAATTTTCTCACTTCACTTAGCAGGTATTTCATCAATTCTTGGAGCCATCAACTTTATTACAACTATTATTAATATACGATGAGAAGGTATATTAATAGAACGACTTCCACTATTTGTGTGATCTGTTTTTATTACCGCAATTTTACTATTATTATCATTACCAGTACTCGCTGGAGCAATTACTATACTTTTAACTGACCGAAATTTTAA

>Durban_South_Africa_(DQ683218)

GTAATTGTTACAGCTCACGCATTTGTTATAATTTTTTTTCTTGTTATACCAGTTATAATTGGAGGATTTGGAAACTGATTAGTTCCTTTAATACTAGGAGCACCAGATATAGCATTCCCACGAATAAATAACATAAGCTTCTGACTCTTACCTCCTTCTCTCACTCTTCTCCTTTCATCTGCAGCTGTTGAAAGAGGTGCAGGTACCGGATGAACCGTTTACCCACCTCTTTCAAGAAATTTAGCTCATATAGGACCCTCTGTTGATCTAGCAATTTTCTCACTTCACTTAGCAGGTATTTCATCAATTCTTGGAGCCATCAACTTTATTACAACTATTATTAATATACGATGAGAAGGTATATTAATAGAACGACTTCCACTATTTGTGTGATCTGTTTTTATTACCGCAATTTTACTATTATTATCATTACCAGTACTCGCTGGAGCAATTACTATACTTTTAACTGACCGAAATTTTAA

>Durban_South_Africa_(DQ683219)

GTAATTGTTACAGCTCACGCATTTGTTATAATTTTTTTTCTTGTTATACCAGTTATAATTGGAGGATTTGGAAACTGATTAGTTCCTTTAATACTAGGAGCACCAGATATAGCATTCCCACGAATAAATAACATAAGCTTCTGACTCTTACCTCCTTCTCTCACTCTTCTCCTTTCATCTGCAGCTGTTGAAAGAGGTGCAGGTACCGGATGAACCGTTTACCCACCTCTTTCAAGAAATTTAGCTCATATAGGACCCTCTGTTGATCTAGCAATTTTCTCACTTCACTTAGCAGGTATTTCATCAATTCTTGGAGCCATCAACTTTATTACAACTATTATTAATATACGATGAGAAGGTATATTAATAGAACGACTTCCACTATTTGTGTGATCTGTTTTTATTACCGCAATTTTACTATTATTATCATTACCAGTACTCGCTGGAGCAATTACTATACTTTTAACTGACCGAAATTTTAA

>Umhlanga_South_Africa_(DQ683220)

GTAATTGTTACAGCTCACGCATTTGTTATAATTTTTTTTCTTGTTATACCAGTTATAATTGGAGGATTTGGAAACTGATTAGTTCCTTTAATACTAGGAGCACCAGATATAGCATTCCCACGAATAAATAACATAAGCTTCTGACTCTTACCTCCTTCTCTCACTCTTCTCCTTTCATCTGCAGCTGTTGAAAGAGGTGCAGGTACCGGATGAACCGTTTACCCACCTCTTTCAAGAAATTTAGCTCATATAGGACCCTCTGTTGATCTAGCAATTTTCTCACTTCACTTAGCAGGTATTTCATCAATTCTTGGAGCCATCAACTTTATTACAACTATTATTAATATACGATGAGAAGGTATATTAATAGAACGACTTCCACTATTTGTGTGATCTGTTTTTATTACCGCAATTTTACTATTATTATCATTACCAGTACTCGCTGGAGCAATTACTATACTTTTAACTGACCGAAATTTTAA

>Galicia_Spain_(DQ683221)

GTAATTGTTACAGCTCACGCATTTGTTATAATTTTTTTTCTTGTTATACCAGTTATAATTGGAGGATTTGGAAACTGATTAGTTCCTTTAATACTAGGAGCACCAGATATAGCATTCCCACGAATAAATAACATAAGCTTCTGACTCTTACCTCCTTCTCTCACTCTTCTCCTTTCATCTGCAGCTGTTGAAAGTGGTGCAGGTACCGGATGAACCGTTTACCCACCTCTTTCAAGAAATTTAGCTCATATAGGACCCTCTGTTGATCTAGCAATTTTCTCACTTCACTTAGCAGGTATTTCATCAATTCTTGGAGCCATCAATTTTATTACAACTATTATTAATATACGATGAGAAGGTATATTAATAGAACGACTTCCACTATTTGTGTGATCTGTTTTTATTACCGCAATTTTACTATTACTATCATTACCAGTACTCGCTGGAGCAATTACTATACTTTTAACTGACCGAAATTTTAA

>Galicia_Spain_(DQ683222)

GTAATTGTTACAGCTCACGCATTTGTTATAATTTTTTTTCTTGTTATACCAGTTATAATTGGAGGATTTGGAAACTGATTAGTTCCTTTAATACTAGGAGCACCAGATATAGCATTCCCACGAATAAATAACATAAGCTTCTGACTCTTACCTCCTTCTCTCACTCTTCTCCTTTCATCTGCAGCTGTTGAAAGTGGTGCAGGTACCGGATGAACCGTTTACCCACCTCTTTCAAGAAATTTAGCTCATATAGGACCCTCTGTTGATCTAGCAATTTTCTCACTTCACTTAGCAGGTATTTCATCAATTCTTGGAGCCATCAATTTTATTACAACTATTATTAATATACGATGAGAAGGTATATTAATAGAACGACTTCCACTATTTGTGTGATCTGTTTTTATTACCGCAATTTTACTATTACTATCATTACCAGTACTCGCTGGAGCAATTACTATACTTTTAACTGACCGAAATTTTAA

>Galicia_Spain_(DQ683223)

GTAATTGTTACAGCTCACGCATTTGTTATAATTTTTTTTCTTGTTATACCAGTTATAATTGGAGGATTTGGAAACTGATTAGTTCCTTTAATACTAGGAGCACCAGATATAGCATTCCCACGAATAAATAACATAAGCTTCTGACTCTTACCTCCTTCTCTCACTCTTCTCCTTTCATCTGCAGCTGTTGAAAGTGGTGCAGGTACCGGATGAACCGTTTACCCACCTCTTTCAAGAAATTTAGCTCATATAGGACCCTCTGTTGATCTAGCAATTTTCTCACTTCACTTAGCAGGTATTTCATCAATTCTTGGAGCCATCAATTTTATTACAACTATTATTAATATACGATGAGAAGGTATATTAATAGAACGACTTCCACTATTTGTGTGATCTGTTTTTATTACCGCAATTTTACTATTACTATCATTACCAGTACTCGCTGGAGCAATTACTATACTTTTAACTGACCGAAATTTTAA

>Senegal_Western_Africa_(DQ683224)

GTAATTGTTACAGCTCACGCATTTGTTATAATTTTTTTTCTTGTTATACCAGTTATAATTGGAGGATTTGGAAACTGATTAGTTCCTTTAATACTAGGAGCACCAGATATAGCATTCCCACGAATAAATAACATAAGCTTCTGACTCTTACCTCCTTCTCTCACTCTTCTCCTTTCATCTGCAGCTGTTGAAAGAGGTGCAGGTACCGGATGAACCGTTTACCCACCTCTTTCAAGAAATTTAGCTCATATAGGACCCTCTGTTGATCTAGCAATTTTCTCACTTCACTTAGCAGGTATTTCATCAATTCTTGGAGCCATCAACTTTATTACAACTATTATTAATATACGATGAGAAGGTATATTAATAGAACGACTTCCACTATTTGTGTGATCTGTTTTTATTACCGCAATTTTACTATTATTATCATTACCAGTACTCGCTGGAGCAATTACTATACTTTTAACTGACCGAAATTTTAA

>Senegal_Western_Africa_(DQ683225)

GTAATTGTTACAGCTCACGCATTTGTTATAATTTTTTTTCTTGTTATACCAGTTATAATTGGAGGATTTGGAAACTGATTAGTTCCTTTAATACTAGGAGCACCAGATATAGCATTCCCACGAATAAATAACATAAGCTTCTGACTCTTACCTCCTTCTCTCACTCTTCTCCTTTCATCTGCAGCTGTTGAAAGAGGTGCAGGTACCGGATGAACCGTTTACCCACCTCTTTCAAGAAATTTAGCTCATATAGGACCCTCTGTTGATCTAGCAATTTTCTCACTTCACTTAGCAGGTATTTCATCAATTCTTGGAGCCATCAACTTTATTACAACTATTATTAATATACGATGAGAAGGTATATTAATAGAACGACTTCCACTATTTGTGTGATCTGTTTTTATTACCGCAATTTTACTATTATTATCATTACCAGTACTCGCTGGAGCAATTACTATACTTTTAACTGACCGAAATTTTAA

>Senegal_Western_Africa_(DQ683226)

GTAATTGTTACAGCTCACGCATTTGTTATAATTTTTTTTCTTGTTATACCAGTTATAATTGGAGGATTTGGAAACTGATTAGTTCCTTTAATACTAGGAGCACCAGATATAGCATTCCCACGAATAAATAACATAAGCTTCTGACTCTTACCTCCTTCTCTCACTCTTCTCCTTTCATCTGCAGCTGTTGAAAGAGGTGCAGGTACCGGATGAACCGTTTACCCACCTCTTTCAAGAAATTTAGCTCATATAGGACCCTCTGTTGATCTAGCAATTTTCTCACTTCACTTAGCAGGTATTTCATCAATTCTTGGAGCCATCAACTTTATTACAACTATTATTAATATACGATGAGAAGGTATATTAATAGAACGACTTCCACTATTTGTGTGATCTGTTTTTATTACCGCAATTTTACTATTATTATCATTACCAGTACTCGCTGGAGCAATTACTATACTTTTAACTGACCGAAATTTTAA

>Spain_Mediterranean_Sea_(DQ683227)

GTAATTGTTACAGCTCACGCATTTGTTATAATTTTTTTTCTTGTTATACCAGTTATAATTGGAGGATTTGGAAACTGATTAGTTCCTTTAATACTAGGAGCACCAGATATAGCATTCCCACGAATAAATAACATAAGCTTCTGACTCTTACCTCCTTCTCTCACTCTTCTCCTTTCATCTGCAGCTGTTGAAAGAGGTGCAGGTACCGGATGAACCGTTTACCCACCTCTTTCAAGAAATTTAGCTCATATAGGACCCTCTGTTGATCTAGCAATTTTCTCACTTCACTTAGCAGGTATTTCATCAATTCTTGGAGCCATCAACTTTATTACAACTATTATTAATATACGATGAGAAGGTATATTAATAGAACGACTTCCACTATTTGTGTGATCTGTTTTTATTACCGCAATTTTACTATTATTATCATTACCAGTACTCGCTGGAGCAATTACTATACTTTTAACTGACCGAAATTTTAA

>Amsterdam_and_Saint_Paul_Islands_Southern_Indian_Ocean_(FN424379)

GTAATTGTTACAGCTCACGCATTTGTTATAATTTTTTTTCTTGTTATACCAGTTATAATTGGAGGATTTGGAAACTGATTAGTTCCTTTAATACTAGGAGCACCAGATATAGCATTCCCACGAATAAATAACATAAGCTTCTGACTCTTACCTCCTTCTCTCACTCTTCTCCTTTCATCTGCAGCTGTTGAAAGAGGTGCAGGTACCGGATGAACCGTTTACCCACCTCTTTCAAGAAATTTAGCTCATATAGGACCCTCTGTTGATCTAGCAATTTTCTCACTTCACTTAGCAGGTATTTCATCAATTCTTGGAGCCATCAACTTTATTACAACTATTATTAATATACGATGAGAAGGTATATTAATAGAACGACTTCCACTATTTGTGTGATCTGTTTTTATTACCGCAATTTTACTATTATTATCATTACCAGTACTCGCTGGAGCAATTACTATACTTTTAACTGACCGAAATTTTAA

>Amsterdam_and_Saint_Paul_Islands_Southern_Indian_Ocean_(FN424380)

GTAATTGTTACAGCTCACGCATTTGTTATAATTTTTTTTCTTGTTATACCAGTTATAATTGGAGGATTTGGAAACTGATTAGTTCCTTTAATACTAGGAGCACCAGATATAGCATTCCCACGAATAAATAACATAAGCTTCTGACTCTTACCTCCTTCTCTCACTCTTCTCCTTTCATCTGCAGCTGTTGAAAGAGGTGCAGGTACCGGATGAACCGTTTACCCACCTCTTTCAAGAAATTTAGCTCATATAGGACCCTCTGTTGATCTAGCAATTTTCTCACTTCACTTAGCAGGTATTTCATCAATTCTTGGAGCCATCAACTTTATTACAACTATTATTAATATACGATGAGAAGGTATATTAATAGAACGACTTCCACTATTTGTGTGATCTGTTTTTATTACCGCAATTTTACTATTATTATCATTACCAGTACTCGCTGGAGCAATTACTATACTTTTAACTGACCGAAATTTTAA

>Amsterdam_and_Saint_Paul_Islands_Southern_Indian_Ocean_(FN424381)

GTAATTGTTACAGCTCACGCATTTGTTATAATTTTTTTTCTTGTTATACCAGTTATAATTGGAGGATTTGGAAACTGATTAGTTCCTTTAATACTAGGAGCACCAGATATAGCATTCCCACGAATAAATAACATAAGCTTCTGACTCTTACCTCCTTCTCTCACTCTTCTCCTTTCATCTGCAGCTGTTGAAAGAGGTGCAGGTACCGGATGAACCGTTTACCCACCTCTTTCAAGAAATTTAGCTCATATAGGACCCTCTGTTGATCTAGCAATTTTCTCACTTCACTTAGCAGGTATTTCATCAATTCTTGGAGCCATCAACTTTATTACAACTATTATTAATATACGATGAGAAGGTATATTAATAGAACGACTTCCACTATTTGTGTGATCTGTTTTTATTACCGCAATTTTACTATTATTATCATTACCAGTACTCGCTGGAGCAATTACTATACTTTTAACTGACCGAAATTTTAA

>Port_Elizabeth_South_Africa_(HM104262)

GTAATTGTTACAGCTCACGCATTTGTTATAATTTTTTTTCTTGTTATACCAGTTATAATTGGAGGATTTGGAAACTGATTAGTTCCTTTAATACTAGGAGCACCAGATATAGCATTCCCACGAATAAATAACATAAGCTTCTGACTCTTACCTCCTTCTCTCACTCTTCTCCTTTCATCTGCAGCTGTTGAAAGAGGTGCAGGTACCGGATGAACCGTTTACCCACCTCTTTCAAGAAATTTAGCTCATATAGGACCCTCTGTTGATCTAGCAATTTTCTCACTTCACTTAGCAGGTATTTCATCAATTCTTGGAGCCATCAACTTTATTACAACTATTATTAATATACGATGAGAAGGTATATTAATAGAACGACTTCCACTATTTGTGTGATCTGTTTTTATTACCGCAATTTTACTATTATTATCATTACCAGTACTCGCTGGAGCAATTACTATACTTTTAACTGACCGAAATTTTAA

>Wenzhou_China_(HQ846110)

GTAATTGTTACAGCTCACGCATTTGTTATAATTTTTTTCCTTGTTATACCAGTTATAATCGGAGGATTTGGAAACTGATTAGTTCCTTTAATACTAGGAGCACCAGATATAGCATTCCCACGAATAAATAATATAAGCTTCTGACTCTTACCTCCTTCTCTTACTCTTCTCCTTTCATCTGCAGCAGTTGAAAGAGGCGCAGGTACCGGATGAACCGTTTACCCGCCTCTTTCAAGAAATTTAGCTCATATAGGACCTTCTGTTGATCTAGCCATTTTCTCACTTCACTTAGCAGGTATTTCATCAATCCTTGGAGCCATCAACTTTATTACAACTATTATTAATATACGATGAGAAGGTATATTAATAGAACGACTTCCACTATTTGTATGATCTGTATTTATTACCGCAATTTTACTATTACTATCATTACCAGTACTCGCTGGAGCAATTACTATACTTTTAACTGACCGAAATTTTAA

>Xiamen_China_(HQ846154)

GTAATTGTTACAGCTCACGCATTTGTTATAATTTTTTTCCTTGTTATACCAGTTATAATCGGAGGATTTGGAAACTGATTAGTTCCTTTAATACTAGGAGCACCAGATATAGCATTCCCACGAATAAATAATATAAGCTTCTGACTCTTACCTCCTTCTCTTACTCTTCTCCTTTCATCTGCAGCAGTTGAAAGAGGTGCAGGTACCGGATGAACCGTTTACCCGCCTCTTTCAAGAAATTTAGCTCATATAGGACCTTCTGTTGATCTAGCCATTTTCTCACTTCACTTAGCAGGTATTTCATCAATCCTTGGAGCCATCAACTTTATTACAACTATTATTAATATACGATGAGAAGGTATATTAATAGAACGACTTCCACTATTTGTATGATCTGTATTTATTACCGCAATTTTACTATTACTATCATTACCAGTACTCGCTGGAGCAATTACCATACTTTTAACTGACCGAAATTTTAA

>Samandag_Turkey_(HQ908426)

GTAATTGTTACAGCTCACGCATTTGTTATAATTTTTTTTCTTGTTATACCAGTTATAATTGGAGGATTTGGAAACTGATTAGTTCCTTTAATACTAGGAGCACCAGATATAGCATTCCCACGAATAAATAACATAAGCTTCTGACTCTTACCTCCTTCTCTCACTCTTCTCCTTTCATCTGCAGCTGTTGAAAGAGGTGCAGGTACCGGATGAACCGTTTACCCACCTCTTTCTAGAAATTTAGCTCATATAGGACCCTCTGTTGATCTAGCAATTTTCTCACTTCACTTAGCAGGTATTTCATCAATTCTTGGAGCCATCAACTTTATTACAACTATTATTAATATACGATGAGAAGGTATATTAATAGAACGACTTCCACTATTTGTGTGATCTGTTTTTATTACCGCAATTTTACTATTATTATCATTACCTGTACTCGCTGGAGCAATTACTATACTTTTAACTGACCGAAATTTTAA

>Yumurtalik_Turkey_(HQ908427)

GTAATTGTTACAGCTCACGCATTTGTTATAATTTTTTTTCTTGTTATACCAGTTATAATTGGAGGATTTGGAAACTGATTAGTTCCTTTAATACTAAGAGCACCAGATATAGCATTCCCACGAATAAATAACATAAGCTTCTGACTCTTACCTCCTTCTCTCACTCTTCTCCTTTCATCTGCAGCTGTTGAAAGAGGTGCAGGTACCGGATGAACCGTTTACCCACCTCTTTCTAGAAATTTAGCTCATATAGGACCCTCTGTTGATCTAGCAATTTTCTCACTTCACTTAGCAGGTATTTCATCAATTCTTGGAGCCATCAACTTTATTACAACTATTATTAATATACGATGAGAAGGTATATTAATAGAACGACTTCCACTATTTGTGTGATCTGTTTTTATTACCGCAATTTTACTATTATTATCATTACCTGTACTCGCTGGAGCAATTACTATACTTTTAACTGACCGAAATTTTAA

>Erdemli_Turkey_(HQ908428)

GTAATTGTTACAGCTCACGCATTTGTTATAATTTTTTTTCTTGTTATACCAGTTATAATTGGAGGATTTGGAAACTGATTAGTTCCTTTAATACTAGGAGCACCAGATATAGCATTCCCACGAATAAATAACATAAGCTTCTGACTCTTACCTCCTTCTCTCACTCTTCTCCTTTCATCTGCAGCTGTTGAAAGAGGTGCAGGTACCGGATGAACCGTTTACCCACCTCTTTCTAGAAATTTAGCTCATATAGGACCCTCTGTTGATCTAGCAATTTTCTCACTTCACTTAGCAGGTATTTCATCAATTCTTGGAGCCATCAACTTTATTACAACTATTATTAATATACGATGAGAAGGTATATTAATAGAACGACTTCCACTATTTGTATGATCTGTTTTTATTACCGCAATTTTACTATTATTATCATTACCTGTACTCGCTGGAGCAATTACTATACTTTTAACTGACCGAAATTTTAA

>Aydincik_Turkey_(HQ908429)

GTAATTGTTACAGCTCACGCATTTGTTATAATTTTTTTTCTTGTTATACCAGTTATAATTGGAGGATTTGGAAACTGATTAGTTCCTTTAATACTAGGAGCACCAGATATAGCATTCCCACGAATAAATAACATAAGCTTCTGACTCTTACCTCCTTCTCTCACTCTTCTCCTTTCATCTGCAGCTGTTGAAAGAGGTGCAGGTACCGGATGAACCGTTTACCCACCTCTTTCTAGAAATTTAGCTCATATAGGACCCTCTGTTGATCTAGCAATTTTCTCACTTCACTTAGCAGGTATTTCATCAATTCTTGGAGCCATCAACTTTATTACAACTATTATTAATATACGATGAGAAGGTATATTAATAGAACGACTTCCACTATTTGTGTGATCTGTTTTTATTACCGCAATTTTACTATTATTATCATTACCTGTACTCGCTGGAGCAATTACTATACTTTTAACTGACCGAAATTTTAA

>Alanya_Turkey_(HQ908430)

GTAATTGTTACAGCTCACGCATTTGTTATAATTTTTTTTCTTGTTATACCAGTTATAATTGGAGGATTTGGAAACTGATTAGTTCCTTTAATACTAGGAGCACCAGATATAGCATTCCCACGAATAAATAACATAAGCTTCTGACTCTTACCTCCTTCTCTCACTCTTCTCCTTTCATCTGCAGCTGTTGAAAGTGGTGCAGGTACCGGATGAACCGTTTACCCACCTCTTTCTAGAAATTTAGCTCATATAGGACCCTCTGTTGATCTAGCAATTTTCTCACTTCACTTAGCAGGTATTTCATCAATTCTTGGAGCCATCAATTTTATTACAACTATTATTAATATACGATGAGAAGGTATATTAATAGAACGACTTCCACTATTTGTGTGATCTGTTTTTATTACCGCAATTTTACTATTACTATCATTACCTGTACTCGCTGGAGCAATTACTATACTTTTAACTGACCGAAATTTTAA

>Kemer_Turkey_(HQ908431)

GTAATTGTTACAGCTCACGCATTTGTTATAATTTTTTTTCTTGTTATACCAGTTATAATTGGAGGATTTGGAAACTGATTAGTTCCTTTAATACTAGGAGCACCAGATATAGCATTCCCACGAATAAATAACATAAGCTTCTGACTATTACCTCCTTCTCTCACTCTTCTCCTTTCATCTGCAGCTGTTGAAAGAGGTGCAGGTACCGGATGAACCGTTTACCCACCTCTTTCTAGAAATTTAGCTCATATAGGACCCTCTGTTGATCTAGCAATTTTCTCACTTCACTTAGCAGGTATTTCATCAATTCTTGGAGCCATCAACTTTATTACAACTATTATTAATATACGATGAGAAGGTATATTAATAGAACGACTCCCACTATTTGTATGATCTGTTTTTATTACCGCAATTTTACTATTACTATCATTACCTGTACTCGCTGGGGCAATTACTATACTTTTAACTGACCGAAATTTTAA

>Fathiye_Turkey_(HQ908432)

GTAATTGTTACAGCTCACGCATTTGTTATAATTTTTTTTCTTGTTATACCAGTTATAATTGGAGGATTTGGAAACTGATTAGTTCCTTTAATACTAGGAGCACCAGATATAGCATTCCCACGAATAAATAACATAAGCTTCTGACTATTACCTCCTTCTCTCACTCTTCTCCTTTCATCTGCAGCTGTTGAAAGAGGTGCAGGTACCGGATGAACCGTTTACCCACCTCTTTCTAGAAATTTAGCTCATATAGGACCCTCTGTTGATTTAGCAATTTTCTCACTTCACTTAGCAGGTATTTCATCAATTCTTGGAGCCATCAACTTTATTACAACTATTATTAATATACGATGAGAAGGTATATTAATAGAACGACTCCCACTATTTGTATGATCTGTTTTTATTACCGCAATTTTACTATTACTATCATTACCTGTACTCGCTGGGGCAATTACTATACTTTTAACTGACCGAAATTTTAA

>Marmaris_Turkey_(HQ908433)

GTAATTGTTACAGCTCACGCATTTGTTATAATTTTTTTCCTTGTTATACCAGTTATAATCGGAGGATTTGGAAACTGATTAGTTCCTTTAATACTAGGAGCACCAGATATAGCATTCCCACGAATAAATAATATAAGCTTCTGACTCTTACCTCCTTCTCTTACTCTTCTCCTTTCATCTGCAGCAGTTGAAAGAGGCGCAGGTACCGGATGAACCGTTTACCCGCCTCTTTCTAGAAATTTAGCTCATATAGGACCTTCTGTTGATCTAGCCATTTTCTCACTTCACTTAGCAGGTATTTCATCAATCCTTGGAGCCATCAACTTTATTACAACTATTATTAATATACGATGAGAAGGTATATTAATAGAACGACTTCCACTATTTGTATGATCTGTATTTATTACCGCAATTTTACTATTACTATCATTACCTGTACTCGCTGGAGCAATTACTATACTTTTAACTGACCGAAATTTTAA

>Datca_Turkey_(HQ908434)

GTAATTGTTACAGCTCACGCATTTGTTATAATTTTTTTCCTTGTTATACCAGTTATAATCGGAGGATTTGGAAACTGATTAGTTCCTTTAATACTAGGAGCACCAGATATAGCATTCCCACGAATAAATAATATAAGCTTCTGACTCTTACCTCCTTCTCTTACTCTTCTCCTTTCATCTGCAGCAGTTGAAAGAGGCGCAGGTACCGGATGAACCGTTTACCCGCCTCTTTCTAGAAATTTAGCTCATATAGGACCTTCTGTTGATCTAGCCATTTTCTCACTTCACTTAGCAGGTATCTCATCAATCCTTGGAGCCATCAACTTTATTACAACTATTATTAATATACGATGAGAAGGTATATTAATAGAACGACTTCCACTATTTGTATGATCTGTATTTATTACCGCAATTTTACTATTACTATCATTACCTGTACTCGCTGGAGCAATTACTATACTTTTAACTGACCGAAATTTTAA

>Turgutreis_Turkey_(HQ908435)

GTAATTGTTACAGCTCACGCATTTGTTATAATTTTTTTCCTTGTTATACCAGTTATAATCGGAGGATTTGGAAACTGATTGGTTCCTTTAATACTAGGAGCACCAGATATAGCATTCCCACGAATAAATAATATAAGCTTCTGACTCTTACCTCCTTCTCTTACTCTTCTCCTTTCATCTGCAGCAGTTGAAAGAGGCGCAGGTACCGGATGAACCGTTTACCCGCCTCTTTCTAGAAATTTAGCTCATATAGGACCTTCTGTTGATCTAGCCATTTTCTCACTTCACTTAGCAGGTATTTCATCAATCCTTGGAGCCATCAACTTTATTACAACTATTATTAATATACGATGAGAAGGTATATTAATAGAACGACTTCCACTATTTGTATGATCTGTATTTATTACCGCAATTTTACTATTACTATCATTACCTGTACTCGCTGGAGCAATTACTATACTTTTAACTGACCGAAATTTTAA

>Didim_Turkey_(HQ908436)

GTAATTGTTACAGCTCACGCATTTGTTATAATTTTTTTCCTTGTTATACCAGTTATAATCGGAGGATTTGGAAACTGATTAGTTCCTTTAATACTAGGAGCACCAGATATAGCATTCCCACGAATAAATAATATAAGCTTCTGACTCTTACCTCCTTCTCTTACTCTTCTCCTTTCATCTGCAGCAGTTGAAAGAGGTGCAGGTACCGGATGAACCGTTTACCCGCTTCTTTCTAGAAATTTAGCTCATATTGGACCTTCTGTTGATCTTGCCATTTTCTCACTTCACTTTGCAGGTATTTCATCAATCCTTGGAGCCATCAACTTTATTACAACTATTATTAATATACGATGAGAAGGTATATTTATAGAACGACTTCCACTATTTGTATGATCTGTATTTATTACCGCAATTTTACTATTACTATCATTACCTGTACTCGCTGGAGCAATTACTATACTTTTTACTGACCGAAATTTTAA

>Fujian_East_China_(JX456270)

GTAATTGTTACAGCTCACGCATTTGTTATAATTTTTTTCCTTGTTATACCAGTTATAATCGGAGGATTTGGAAACTGATTAGTTCCTTTAATACTAGGAGCACCAGATATAGCATTCCCACGAATAAATAATATAAGCTTCTGACTCTTACCTCCTTCTCTTACTCTTCTCCTTTCATCTGCAGCAGTTGAAAGAGGCGCAGGTACCGGATGAACCGTTTACCCGCCTCTTTCAAGAAATTTAGCTCATATAGGACCTTCTGTTGATCTAGCCATTTTCTCACTTCACTTAGCAGGTATTTCATCAATCCTTGGAGCCATCAACTTTATTACAACTATTATTAATATACGATGAGAAGGTATATTAATAGAACGACTTCCACTATTTGTATGATCTGTATTTATTACCGCAATTTTACTATTACTATCATTACCAGTACTCGCTGGAGCAATTACTATACTTTTAACTGACCGAAATTTTAA

>Yumurtalik_Turkey_(KC311408)

GTAATTGTTACAGCTCACGCATTTGTTATAATTTTTTTTCTTGTTATACCAGTTATAATTGGAGGATTTGGAAACTGATTAGTTCCTTTAATACTAGGAGCACCAGATATAGCATTCCCACGAATAAATAACATAAGCTTCTGACTCTTACCTCCTTCTCTCACTCTTCTCCTTTCATCTGCAGCTGTTGAAAGTGGTGCAGGTACCGGATGAACCGTTTACCCACCTCTTTCTAGTAATTTAGCTCATATAGGACCCTCTGTTGATCTAGCAATTTTCTCACTTCACTTAGCAGGTATTTCATCAATTCTTGGAGCCATCAACTTTATTACAACTATTATTAATATACGATGAGAAGGTATATTAATAGAACGACTTCCACTATTTGTGTGATCTGTTTTTATTACCGCAATTTTACTATTATTATCATTACCTGTACTCGCTGGAGCAATTACTATACTTTTAACTGACCGAAATTTTAA

>Yumurtalik_Turkey_(KC311409)

GTAATTGTTACAGCTCACGCATTTGTTATAATTTTTTTTCTTGTTATACCAGTTATAATTGGAGGATTTGGAAACTGATTAGTTCCTTTAATACTAGGAGCACCAGATATAGCATTCCCACGAATAAATAACATAAGCTTCTGACTCTTACCTCCTTCTCTCACTCTTCTCCTTTCATCTGCAGCTGTTGAAAGAGGTGCAGGTACCGGATGAACCGTTTACCCACCTCTTTCTAGAAATTTAGCTCATATAGGACCCTCTGTTGATCTAGCAATTTTCTCACTTCACTTAGCAGGTATTTCATCAATTCTTGGAGCCATCAACTTTATTACAACTATTATTAATATACGATGAGAAGGTATATTAATAGAACGACTTCCACTATTTGTGTGATCTGTTTTTATTACCGCAATTTTACTATTATTATCATTACCTGTACTCGCTGGAGCAATTACTATACTTTTAACTGACCGAAATTTTAA

>Yumurtalik_Turkey_(KC311410)

GTAATTGTTACAGCTCACGCATTTGTTATAATTTTTTTTCTTGTTATACCAGTTATAATTGGAGGATTTGGAAACTGATTAGTTCCTTTAATACTAGGAGCACCAGATATAGCATTCCCACGAATAAATAACATAAGCTTCTGACTATTACCTCCTTCTCTCACTCTTCTCCTTTCATCTGCAGCTGTTGAAAGAGGTGCAGGTACCGGATGAACCGTTTACCCACCTCTTTCTAGAAATTTAGCTCATATAGGACCCTCTGTTGATTTAGCAATTTTCTCACTTCACTTAGCAGGTATTTCATCAATTCTTGGAGCCATCAACTTTATTACAACTATTATTAATATACGATGAGAAGGTATATTAATAGAACGACTCCCACTATTTGTATGATCTGTTTTTATTACCGCAATTTTACTATTACTATCATTACCTGTACTCGCTGGGGCAATTACTATACTTTTAACTGACCGAAATTTTAA

>Yumurtalik_Turkey_(KC311411)

GTAATTGTTACAGCTCACGCATTTGTTATAATTTTTTTCCTTGTTATACCAGTTATAATCGGAGGATTTGGAAACTGATTAGTTCCTTTAATACTAGGAGCACCAGATATAGCATTCCCACGAATAAATAATATAAGCTTCTGACTCTTACCTCCTTCTCTTACTCTTCTCCTTTCATCTGCAGCAGTTGAAAGAGGTGCAGGTACCGGATGAACCGTTTACCCGCTTCTTTCTAGAAATTTAGCTCATATTGGACCTTCTGTTGATCTTGCCATTTTCTCACTTCACTTTGCAGGTATTTCATCAATCCTTGGAGCCATCAACTTTATTACAACTATTATTAATATACGATGAGAAGGTATATTTATAGAACGACTTCCACTATTTGTATGATCTGTATTTATTACCGCAATTTTACTATTACTATCATTACCTGTACTCGCTGGAGCAATTACTATACTTTTTACTGACCGAAATTTTAA

>Yumurtalik_Turkey_(KC311412)

GTAATTGTTACAGCTCACGCATTTGTTATAATTTTTTTTCTTGTTATACCAGTTATAATTGGAGGATTTGGAAACTGATTAGTTCCTTTAATACTAGGAGCACCAGATATAGCATTCCCACGAATAAATAACATAAGCTTCTGACTCTTACCTCCTTCTCTCACTCTTCTCCTTTCATCTGCAGCTGTTGAAAGAGGTGCAGGTACCGGATGAACCGTTTACCCACCTCTTTCAAGAAATTTAGCTCATATAGGACCCTCTGTTGATCTAGCAATTTTCTCACTTCACTTAGCAGGTATTTCATCAATTCTTGGAGCCATCAACTTTATTACAACTATTATTAATATACGATGAGAAGGTATATTAATAGAACGACTTCCACTATTTGTGTGATCTGTTTTTATTACCGCAATTTTACTATTATTATCATTACCAGTACTCGCTGGAGCAATTACTATACTTTTAACTGACCGAAATTTTAA

>Turkey_(KC789314)

GTAATTGTTACAGCTCACGCATTTGTTATAATTTTTTTCCTTGTTATACCAGTTATAATCGGAGGATTTGGAAACTGATTAGTTCCTTTAATACTAGGAGCACCAGATATAGCATTCCCACGAATAAATAATATAAGCTTCTGACTCTTACCTCCTTCTCTTACTCTTCTCCTTTCATCTGCAGCAGTTGAAAGAGGTGCAGGTACCGGATGAACCGTTTACCCGCTTCTTTCTAGAAATTTAGCTCATATTGGACCTTCTGTTGATCTTGCCATTTTCTCACTTCACTTTGCAGGTATTTCATCAATCCTTGGAGCCATCAACTTTATTACAACTATTATTAATATACGATGAGAAGGTATATTTATAGAACGACTTCCACTATTTGTATGATCTGTATTTATTACCGCAATTTTACTATTACTATCATTACCTGTACTCGCTGGAGCAATTACTATACTTTTTACTGACCGAAATTTTAA

>Turkey_(KC789315)

GTAATTGTTACAGCTCACGCATTTGTTATAATTTTTTTTCTTGTTATACCAGTTATAATTGGAGGATTTGGAAACTGATTAGTTCCTTTAATACTAGGAGCACCAGATATAGCATTCCCACGAATAAATAACATAAGCTTCTGACTCTTACCTCCTTCTCTCACTCTTCTCCTTTCATCTGCAGCTGTTGAAAGAGGTGCAGGTACCGGATGAACCGTTTACCCACCTCTTTCAAGAAATTTAGCTCATATAGGACCCTCTGTTGATCTAGCAATTTTCTCACTTCACTTAGCAGGTATTTCATCAATTCTTGGAGCCATCAACTTTATTACAACTATTATTAATATACGATGAGAAGGTATATTAATAGAACGACTTCCACTATTTGTGTGATCTGTTTTTATTACCGCAATTTTACTATTATTATCATTACCAGTACTCGCTGGAGCAATTACTATACTTTTAACTGACCGAAATTTTAA

>Turkey_(KC789316)

GTAATTGTTACAGCTCACGCATTTGTTATAATTTTTTTTCTTGTTATACCAGTTATAATTGGAGGATTTGGAAACTGATTAGTTCCTTTAATACTAGGAGCACCAGATATAGCATTCCCACGAATAAATAACATAAGCTTCTGACTCTTACCTCCTTCTCTCACTCTTCTCCTTTCATCTGCAGCTGTTGAAAGTGGTGCAGGTACCGGATGAACCGTTTACCCACCTCTTTCTAGTAATTTAGCTCATATAGGACCCTCTGTTGATCTAGCAATTTTCTCACTTCACTTAGCAGGTATTTCATCAATTCTTGGAGCCATCAACTTTATTACAACTATTATTAATATACGATGAGAAGGTATATTAATAGAACGACTTCCACTATTTGTGTGATCTGTTTTTATTACCGCAATTTTACTATTATTATCATTACCTGTACTCGCTGGAGCAATTACTATACTTTTAACTGACCGAAATTTTAA

>Turkey_(KC789317)

GTAATTGTTACAGCTCACGCATTTGTTATAATTTTTTTTCTTGTTATACCAGTTATAATTGGAGGATTTGGAAACTGATTAGTTCCTTTAATACTAGGAGCACCAGATATAGCATTCCCACGAATAAATAACATAAGCTTCTGACTCTTACCTCCTTCTCTCACTCTTCTCCTTTCATCTGCAGCTGTTGAAAGTGGTGCAGGTACCGGATGAACCGTTTACCCACCTCTTTCTAGTAATTTAGCTCATATAGGACCCTCTGTTGATCTAGCAATTTTCTCACTTCACTTAGCAGGTATTTCATCAATTCTTGGAGCCATCAACTTTATTACAACTATTATTAATATACGATGAGAAGGTATATTAATAGAACGACTTCCACTATTTGTGTGATCTGTTTTTATTACCGCAATTTTACTATTATTATCATTACCTGTACTCGCTGGAGCAATTACTATACTTTTAACTGACCGAAATTTTAA

>Turkey_(KC789318)

GTAATTGTTACAGCTCACGCATTTGTTATAATTTTTTTTCTTGTTATACCAGTTATAATTGGAGGATTTGGAAACTGATTAGTTCCTTTAATACTAGGAGCACCAGATATAGCATTCCCACGAATAAATAACATAAGCTTCTGACTCTTACCTCCTTCTCTCACTCTTCTCCTTTCATCTGCAGCTGTTGAAAGTGGTGCAGGTACCGGATGAACCGTTTACCCACCTCTTTCTAGTAATTTAGCTCATATAGGACCCTCTGTTGATCTAGCAATTTTCTCACTTCACTTAGCAGGTATTTCATCAATTCTTGGAGCCATCAACTTTATTACAACTATTATTAATATACGATGAGAAGGTATATTAATAGAACGACTTCCACTATTTGTGTGATCTGTTTTTATTACCGCAATTTTACTATTATTATCATTACCTGTACTCGCTGGAGCAATTACTATACTTTTAACTGACCGAAATTTTAA

>Turkey_(KC789319)

GTAATTGTTACAGCTCACGCATTTGTTATAATTTTTTTTCTTGTTATACCAGTTATAATTGGAGGATTTGGAAACTGATTAGTTCCTTTAATACTAGGAGCACCAGATATAGCATTCCCACGAATAAATAACATAAGCTTCTGACTCTTACCTCCTTCTCTCACTCTTCTCCTTTCATCTGCAGCTGTTGAAAGTGGTGCAGGTACCGGATGAACCGTTTACCCACCTCTTTCTAGTAATTTAGCTCATATAGGACCCTCTGTTGATCTAGCAATTTTCTCACTTCACTTAGCAGGTATTTCATCAATTCTTGGAGCCATCAACTTTATTACAACTATTATTAATATACGATGAGAAGGTATATTAATAGAACGACTTCCACTATTTGTGTGATCTGTTTTTATTACCGCAATTTTACTATTATTATCATTACCTGTACTCGCTGGAGCAATTACTATACTTTTAACTGACCGAAATTTTAA

>Turkey_(KC789320)

GTAATTGTTACAGCTCACGCATTTGTTATAATTTTTTTTCTTGTTATACCAGTTATAATTGGAGGATTTGGAAACTGATTAGTTCCTTTAATACTAGGAGCACCAGATATAGCATTCCCACGAATAAATAACATAAGCTTCTGACTCTTACCTCCTTCTCTCACTCTTCTCCTTTCATCTGCAGCTGTTGAAAGTGGTGCAGGTACCGGATGAACCGTTTACCCACCTCTTTCTAGTAATTTAGCTCATATAGGACCCTCTGTTGATCTAGCAATTTTCTCACTTCACTTAGCAGGTATTTCATCAATTCTTGGAGCCATCAACTTTATTACAACTATTATTAATATACGATGAGAAGGTATATTAATAGAACGACTTCCACTATTTGTGTGATCTGTTTTTATTACCGCAATTTTACTATTATTATCATTACCTGTACTCGCTGGAGCAATTACTATACTTTTAACTGACCGAAATTTTAA

>Turkey_(KC789321)

GTAATTGTTACAGCTCACGCATTTGTTATAATTTTTTTTCTTGTTATACCAGTTATAATTGGAGGATTTGGAAACTGATTAGTTCCTTTAATACTAGGAGCACCAGATATAGCATTCCCACGAATAAATAACATAAGCTTCTGACTCTTACCTCCTTCTCTCACTCTTCTCCTTTCATCTGCAGCTGTTGAAAGTGGTGCAGGTACCGGATGAACCGTTTACCCACCTCTTTCTAGTAATTTAGCTCATATAGGACCCTCTGTTGATCTAGCAATTTTCTCACTTCACTTAGCAGGTATTTCATCAATTCTTGGAGCCATCAACTTTATTACAACTATTATTAATATACGATGAGAAGGTATATTAATAGAACGACTTCCACTATTTGTGTGATCTGTTTTTATTACCGCAATTTTACTATTATTATCATTACCTGTACTCGCTGGAGCAATTACTATACTTTTAACTGACCGAAATTTTAA

>Turkey_(KC789322)

GTAATTGTTACAGCTCACGCATTTGTTATAATTTTTTTTCTTGTTATACCAGTTATAATTGGAGGATTTGGAAACTGATTAGTTCCTTTAATACTAGGAGCACCAGATATAGCATTCCCACGAATAAATAACATAAGCTTCTGACTCTTACCTCCTTCTCTCACTCTTCTCCTTTCATCTGCAGCTGTTGAAAGTGGTGCAGGTACCGGATGAACCGTTTACCCACCTCTTTCTAGTAATTTAGCTCATATAGGACCCTCTGTTGATCTAGCAATTTTCTCACTTCACTTAGCAGGTATTTCATCAATTCTTGGAGCCATCAACTTTATTACAACTATTATTAATATACGATGAGAAGGTATATTAATAGAACGACTTCCACTATTTGTGTGATCTGTTTTTATTACCGCAATTTTACTATTATTATCATTACCTGTACTCGCTGGAGCAATTACTATACTTTTAACTGACCGAAATTTTAA

>Turkey_(KC789323)

GTAATTGTTACAGCTCACGCATTTGTTATAATTTTTTTTCTTGTTATACCAGTTATAATTGGAGGATTTGGAAACTGATTAGTTCCTTTAATACTAGGAGCACCAGATATAGCATTCCCACGAATAAATAACATAAGCTTCTGACTCTTACCTCCTTCTCTCACTCTTCTCCTTTCATCTGCAGCTGTTGAAAGTGGTGCAGGTACCGGATGAACCGTTTACCCACCTCTTTCTAGTAATTTAGCTCATATAGGACCCTCTGTTGATCTAGCAATTTTCTCACTTCACTTAGCAGGTATTTCATCAATTCTTGGAGCCATCAACTTTATTACAACTATTATTAATATACGATGAGAAGGTATATTAATAGAACGACTTCCACTATTTGTGTGATCTGTTTTTATTACCGCAATTTTACTATTATTATCATTACCTGTACTCGCTGGAGCAATTACTATACTTTTAACTGACCGAAATTTTAA

>Turkey_(KC789324)

GTAATTGTTACAGCTCACGCATTTGTTATAATTTTTTTTCTTGTTATACCAGTTATAATTGGAGGATTTGGAAACTGATTAGTTCCTTTAATACTAGGAGCACCAGATATAGCATTCCCACGAATAAATAACATAAGCTTCTGACTCTTACCTCCTTCTCTCACTCTTCTCCTTTCATCTGCAGCTGTTGAAAGAGGTGCAGGTACCGGATGAACCGTTTACCCACCTCTTTCTAGAAATTTAGCTCATATAGGACCCTCTGTTGATCTAGCAATTTTCTCACTTCACTTAGCAGGTATTTCATCAATTCTTGGAGCCATCAACTTTATTACAACTATTATTAATATACGATGAGAAGGTATATTAATAGAACGACTTCCACTATTTGTGTGATCTGTTTTTATTACCGCAATTTTACTATTATTATCATTACCTGTACTCGCTGGAGCAATTACTATACTTTTAACTGACCGAAATTTTAA

>Turkey_KC789325)

GTAATTGTTACAGCTCACGCATTTGTTATAATTTTTTTTCTTGTTATACCAGTTATAATTGGAGGATTTGGAAACTGATTAGTTCCTTTAATACTAGGAGCACCAGATATAGCATTCCCACGAATAAATAACATAAGCTTCTGACTCTTACCTCCTTCTCTCACTCTTCTCCTTTCATCTGCAGCTGTTGAAAGAGGTGCAGGTACCGGATGAACCGTTTACCCACCTCTTTCTAGAAATTTAGCTCATATAGGACCCTCTGTTGATCTAGCAATTTTCTCACTTCACTTAGCAGGTATTTCATCAATTCTTGGAGCCATCAACTTTATTACAACTATTATTAATATACGATGAGAAGGTATATTAATAGAACGACTTCCACTATTTGTGTGATCTGTTTTTATTACCGCAATTTTACTATTATTATCATTACCTGTACTCGCTGGAGCAATTACTATACTTTTAACTGACCGAAATTTTAA

>Turkey_(KC789326)

GTAATTGTTACAGCTCACGCATTTGTTATAATTTTTTTTCTTGTTATACCAGTTATAATTGGAGGATTTGGAAACTGATTAGTTCCTTTAATACTAGGAGCACCAGATATAGCATTCCCACGAATAAATAACATAAGCTTCTGACTCTTACCTCCTTCTCTCACTCTTCTCCTTTCATCTGCAGCTGTTGAAAGAGGTGCAGGTACCGGATGAACCGTTTACCCACCTCTTTCTAGAAATTTAGCTCATATAGGACCCTCTGTTGATCTAGCAATTTTCTCACTTCACTTAGCAGGTATTTCATCAATTCTTGGAGCCATCAACTTTATTACAACTATTATTAATATACGATGAGAAGGTATATTAATAGAACGACTTCCACTATTTGTGTGATCTGTTTTTATTACCGCAATTTTACTATTATTATCATTACCTGTACTCGCTGGAGCAATTACTATACTTTTAACTGACCGAAATTTTAA

>Turkey_(KC789327)

GTAATTGTTACAGCTCACGCATTTGTTATAATTTTTTTTCTTGTTATACCAGTTATAATTGGAGGATTTGGAAACTGATTAGTTCCTTTAATACTAGGAGCACCAGATATAGCATTCCCACGAATAAATAACATAAGCTTCTGACTCTTACCTCCTTCTCTCACTCTTCTCCTTTCATCTGCAGCTGTTGAAAGAGGTGCAGGTACCGGATGAACCGTTTACCCACCTCTTTCTAGAAATTTAGCTCATATAGGACCCTCTGTTGATCTAGCAATTTTCTCACTTCACTTAGCAGGTATTTCATCAATTCTTGGAGCCATCAACTTTATTACAACTATTATTAATATACGATGAGAAGGTATATTAATAGAACGACTTCCACTATTTGTGTGATCTGTTTTTATTACCGCAATTTTACTATTATTATCATTACCTGTACTCGCTGGAGCAATTACTATACTTTTAACTGACCGAAATTTTAA

>Turkey_(KC789328)

GTAATTGTTACAGCTCACGCATTTGTTATAATTTTTTTTCTTGTTATACCAGTTATAATTGGAGGATTTGGAAACTGATTAGTTCCTTTAATACTAGGAGCACCAGATATAGCATTCCCACGAATAAATAACATAAGCTTCTGACTCTTACCTCCTTCTCTCACTCTTCTCCTTTCATCTGCAGCTGTTGAAAGTGGTGCAGGTACCGGATGAACCGTTTACCCACCTCTTTCTAGTAATTTAGCTCATATAGGACCCTCTGTTGATCTAGCAATTTTCTCACTTCACTTAGCAGGTATTTCATCAATTCTTGGAGCCATCAACTTTATTACAACTATTATTAATATACGATGAGAAGGTATATTAATAGAACGACTTCCACTATTTGTGTGATCTGTTTTTATTACCGCAATTTTACTATTATTATCATTACCTGTACTCGCTGGAGCAATTACTATACTTTTAACTGACCGAAATTTTAA

>Turkey_(KC789329)

GTAATTGTTACAGCTCACGCATTTGTTATAATTTTTTTTCTTGTTATACCAGTTATAATTGGAGGATTTGGAAACTGATTAGTTCCTTTAATACTAGGAGCACCAGATATAGCATTCCCACGAATAAATAACATAAGCTTCTGACTCTTACCTCCTTCTCTCACTCTTCTCCTTTCATCTGCAGCTGTTGAAAGAGGTGCAGGTACCGGATGAACCGTTTACCCACCTCTTTCTAGAAATTTAGCTCATATAGGACCCTCTGTTGATCTAGCAATTTTCTCACTTCACTTAGCAGGTATTTCATCAATTCTTGGAGCCATCAACTTTATTACAACTATTATTAATATACGATGAGAAGGTATATTAATAGAACGACTTCCACTATTTGTGTGATCTGTTTTTATTACCGCAATTTTACTATTATTATCATTACCTGTACTCGCTGGAGCAATTACTATACTTTTAACTGACCGAAATTTTAA

>Turkey_(KC789330)

GTAATTGTTACAGCTCACGCATTTGTTATAATTTTTTTTCTTGTTATACCAGTTATAATTGGAGGATTTGGAAACTGATTAGTTCCTTTAATACTAGGAGCACCAGATATAGCATTCCCACGAATAAATAACATAAGCTTCTGACTATTACCTCCTTCTCTCACTCTTCTCCTTTCATCTGCAGCTGTTGAAAGAGGTGCAGGTACCGGATGAACCGTTTACCCACCTCTTTCTAGAAATTTAGCTCATATAGGACCCTCTGTTGATTTAGCAATTTTCTCACTTCACTTAGCAGGTATTTCATCAATTCTTGGAGCCATCAACTTTATTACAACTATTATTAATATACGATGAGAAGGTATATTAATAGAACGACTCCCACTATTTGTATGATCTGTTTTTATTACCGCAATTTTACTATTACTATCATTACCTGTACTCGCTGGGGCAATTACTATACTTTTAACTGACCGAAATTTTAA

>Turkey_(KC789331)

GTAATTGTTACAGCTCACGCATTTGTTATAATTTTTTTTCTTGTTATACCAGTTATAATTGGAGGATTTGGAAACTGATTAGTTCCTTTAATACTAGGAGCACCAGATATAGCATTCCCACGAATAAATAACATAAGCTTCTGACTATTACCTCCTTCTCTCACTCTTCTCCTTTCATCTGCAGCTGTTGAAAGAGGTGCAGGTACCGGATGAACCGTTTACCCACCTCTTTCTAGAAATTTAGCTCATATAGGACCCTCTGTTGATTTAGCAATTTTCTCACTTCACTTAGCAGGTATTTCATCAATTCTTGGAGCCATCAACTTTATTACAACTATTATTAATATACGATGAGAAGGTATATTAATAGAACGACTCCCACTATTTGTATGATCTGTTTTTATTACCGCAATTTTACTATTACTATCATTACCTGTACTCGCTGGGGCAATTACTATACTTTTAACTGACCGAAATTTTAA

>Turkey_(KC789332)

GTAATTGTTACAGCTCACGCATTTGTTATAATTTTTTTTCTTGTTATACCAGTTATAATTGGAGGATTTGGAAACTGATTAGTTCCTTTAATACTAGGAGCACCAGATATAGCATTCCCACGAATAAATAACATAAGCTTCTGACTATTACCTCCTTCTCTCACTCTTCTCCTTTCATCTGCAGCTGTTGAAAGAGGTGCAGGTACCGGATGAACCGTTTACCCACCTCTTTCTAGAAATTTAGCTCATATAGGACCCTCTGTTGATTTAGCAATTTTCTCACTTCACTTAGCAGGTATTTCATCAATTCTTGGAGCCATCAACTTTATTACAACTATTATTAATATACGATGAGAAGGTATATTAATAGAACGACTCCCACTATTTGTATGATCTGTTTTTATTACCGCAATTTTACTATTACTATCATTACCTGTACTCGCTGGGGCAATTACTATACTTTTAACTGACCGAAATTTTAA

>Turkey_(KC789333)

GTAATTGTTACAGCTCACGCATTTGTTATAATTTTTTTTCTTGTTATACCAGTTATAATTGGAGGATTTGGAAACTGATTAGTTCCTTTAATACTAGGAGCACCAGATATAGCATTCCCACGAATAAATAACATAAGCTTCTGACTATTACCTCCTTCTCTCACTCTTCTCCTTTCATCTGCAGCTGTTGAAAGAGGTGCAGGTACCGGATGAACCGTTTACCCACCTCTTTCTAGAAATTTAGCTCATATAGGACCCTCTGTTGATTTAGCAATTTTCTCACTTCACTTAGCAGGTATTTCATCAATTCTTGGAGCCATCAACTTTATTACAACTATTATTAATATACGATGAGAAGGTATATTAATAGAACGACTCCCACTATTTGTATGATCTGTTTTTATTACCGCAATTTTACTATTACTATCATTACCTGTACTCGCTGGGGCAATTACTATACTTTTAACTGACCGAAATTTTAA

>Vigo_Spain_(KC851954)

GTAATTGTTACAGCTCACGCATTTGTTATAATTTTTTTTCTTGTTATACCAGTTATAATTGGAGGATTTGGAAACTGATTAGTTCCTTTAATACTAGGAGCACCAGATATAGCATTCCCACGAATAAATAACATAAGCTTCTGACTCTTACCTCCTTCTCTCACTCTTCTCCTTTCATCTGCAGCTGTTGAAAGTGGTGCAGGTACCGGATGAACCGTTTACCCACCTCTTTCAAGAAATTTAGCTCATATAGGACCCTCTGTTGATCTAGCAATTTTCTCACTTCACTTAGCAGGTATTTCATCAATTCTTGGAGCCATCAATTTTATTACAACTATTATTAATATACGATGAGAAGGTATATTAATAGAACGACTTCCACTATTTGTGTGATCTGTTTTTATTACCGCAATTTTACTATTACTATCATTACCAGTACTCGCTGGAGCAATTACTATACTTTTAACTGACCGAAATTTTAA

>Vigo_Spain_(KC851955)

GTAATTGTTACAGCTCACGCATTTGTTATAATTTTTTTTCTTGTTATACCAGTTATAATTGGAGGATTTGGAAACTGATTAGTTCCTTTAATACTAGGAGCACCAGATATAGCATTCCCACGAATAAATAACATAAGCTTCTGACTCTTACCTCCTTCTCTCACTCTTCTCCTTTCATCTGCAGCTGTTGAAAGTGGTGCAGGTACCGGATGAACCGTTTACCCACCTCTTTCAAGAAATTTAGCTCATATAGGACCCTCTGTTGATCTAGCAATTTTCTCACTTCACTTAGCAGGTATTTCATCAATTCTTGGAGCCATCAATTTTATTACAACTATTATTAATATACGATGAGAAGGTATATTAATAGAACGACTTCCACTATTTGTGTGATCTGTTTTTATTACCGCAATTTTACTATTACTATCATTACCAGTACTCGCTGGAGCAATTACTATACTTTTAACTGACCGAAATTTTAA

>Vigo_Spain_(KC851956)

GTAATTGTTACAGCTCACGCATTTGTTATAATTTTTTTTCTTGTTATACCAGTTATAATTGGAGGATTTGGAAACTGATTAGTTCCTTTAATACTAGGAGCACCAGATATAGCATTCCCACAAATAAATAACATAAGCTTCTGACTCTTACCTCCTTCTCTCACTCTTCTCCTTTCATCTGCAGCTGTTGAAAGTGGTGCAGGTACCGGATGAACCGTTTACCCACCTCTTTCAAGAAATTTAGCTCATATAGGACCCTCTGTTGATCTAGCAATTTTCTCACTTCACTTAGCAGGTATTTCATCAATTCTTGGAGCCATCAATTTTATTACAACTATTATTAATATACGATGAGAAGGTATATTAATAGAACGACTTCCACTATTTGTGTGATCTGTTTTTATTACCGCAATTTTACTATTACTATCATTACCAGTACTCGCTGGAGCAATTACTATACTTTTAACTGACCGAAATTTTAA

>Vigo_Spain_(KC851957)

GTAATTGTTACAGCTCACGCATTTGTTATAATTTTTTTTCTTGTTATACCAGTTATAATTGGAGGATTTGGAAACTGATTAGTTCCTTTAATACTAGGAGCACCAGATATAGCATTCCCACAAATAAATAACATAAGCTTCTGACTCTTACCTCCTTCTCTCACTCTTCTCCTTTCATCTGCAGCTGTTGAAAGAGGTGCAGGTACCGGATGAACCGTTTACCCACCTCTTTCAAGAAATTTAGCTCATATAGGACCCTCTGTTGATCTAGCAATTTTCTCACTTCACTTAGCAGGTATTTCATCAATTCTTGGAGCCATCAATTTTATTACAACTATTATTAATATACGATGAGAAGGTATATTAATAGAACGACTTCCACTATTTGTGTGATCTGTTTTTATTACCGCAATTTTACTATTACTATCATTACCAGTACTCGCTGGAGCAATTACTATACTTTTAACTGACCGAAATTTTAA

>Vigo_Spain_(KC851958)

GTAATTGTTACAGCTCACGCATTTGTTATAATTTTTTTTCTTGTTATACCAGTTATAATTGGAGGATTTGGAAACTGATTAGTTCCTTTAATACTAGGAGCACCAGATATAGCATTCCCACAAATAAATAACATAAGCTTCTGACTCTTACCTCCTTCTCTCACTCTTCTCCTTTCATCTGCAGCTGTTGAAAGTGGTGCAGGTACCGGATGAACCGTTTACCCACCTCTTTCAAGAAATTTAGCTCATATAGGACCCTCTGTTGATCTAGCAATTTTCTCACTTCACTTAGCAGGTATTTCATCAATTCTTGGAGCCATCAATTTTATTACAACTATTATTAATATACGATGAGAAGGTATATTAATAGAACGACTTCCACTATTTGTGTGATCTGTTTTTATTACCGCAATTTTACTATTACTATCATTACCAGTACTCGCTGGAGCAATTACTATACTTTTAACTGACCGAAATTTTAA

>Vigo_Spain_(KC851959)

GTAATTGTTACAGCTCACGCATTTGTTATAATTTTTTTTCTTGTTATACCAGTTATAATTGGAGGATTTGGAAACTGATTAGTTCCTTTAATACTAGGAGCACCAGATATAGCATTCCCACAAATAAATAACATAAGCTTCTGACTCTTACCTCCTTCTCTCACTCTTCTCCTTTCATCTGCAGCTGTTGAAAGTGGTGCAGGTACCGGATGAACCGTTTACCCACCTCTTTCAAGAAATTTAGCTCATATAGGACCCTCTGTTGATCTAGCAATTTTCTCACTTCACTTAGCAGGTATTTCATCAATTCTTGGAGCCATCAATTTTATTACAACTATTATTAATATACGATGAGAAGGTATATTAATAGAACGACTTCCACTATTTGTGTGATCTGTTTTTATTACCGCAATTTTACTATTACTATCATTACCAGTACTCGCTGGAGCAATTACGATACTTTTAACTGACCGAAATTTTAA

>Vigo_Spain_(KC851960)

GTAATTGTTACAGCTCACGCATTTGTTATAATTTTTTTTCTTGTTATACCAGTTATAATTGGAGGATTTGGAAACTGATTAGTTCCTTTAATACTAGGAGCACCAGATATAGCATTCCCACGAATAAATAACATAAGCTTCTGACTCTTACCTCCTTCTCTCACTCTTCTCCTTTCATCTGCAGCTGTTGAAAGAGGTGCAGGTACCGGATGAACCGTTTACCCACCTCTTTCAAGAAATTTAGCTCATATAGGACCCTCTGTTGATCTAGCAATTTTCTCACTTCACTTAGCAGGTATTTCATCAATTCTTGGAGCCATCAACTTTATTACAACTATTATTAATATACGATGAGAAGGTATATTAATAGAACGACTTCCACTATTTGTGTGATCTGTTTTTATTACCGCAATTTTACTATTATTATCATTACCAGTACTCGCTGGAGCAATTACTATACTTTTAACTGACCGAAATTTTAA

>Vigo_Spain_(KC851961)

GTAATTGTTACAGCTCACGCATTTGTTATAATTTTTTTTCTTGTTATACCAGTTATAATTGGAGGATTTGGAAACTGATTAGTTCCTTTAATACTAGGAGCACCAGATATAGCATTCCCACGAATAAATAACATAAGCTTCTGACTCTTACCTCCTTCTCTCACTCTTCTCCTTTCATCTGCAGCTGTTGAAAGAGGTGCAGGTACCGGATGAACCGTTTACCCACCTCTTTCAAGAAATTTAGCTCATATAGGACCCTCTGTTGATCTAGCAATTTTCTCACTTCACTTAGCAGGTATTTCATCAATTCTTGGAGCCATCAACTTTATTACAACTATTATTAATATACGATGAGAAGGTATATTAATAGAACGACTTCCACTATTTGTGTGATCTGTTTTTATTACCGCAATTTTACTATTATTATCATTACCAGTACTCGCTGGAGCAATTACTATACTTTTAACTGACCGAAATTTTAA

>Vigo_Spain_(KC851962)

GTAATTGTTACAGCTCACGCATTTGTTATAATTTTTTTTCTTGTTATACCAGTTATAATTGGAGGATTTGGAAACTGATTAGTTCCTTTAATACTAGGAGCACCAGATATAGCATTCCCACGAATAAATAACATAAGCTTCTGACTCTTACCTCCTTCTCTCACTCTTCTCCTTTCATCTGCAGCTGTTGAAAGTGGTGCAGGTACCGGATGAACCGTTTACCCCCCTCTTTCAAGAAATTTAGCTCATATAGGACCCTCTGTTGATCTAGCAATTTTCTCACTTCACTTAGCAGGTATTTCATCAATTCTTGGAGCCATCAATTTTATTACAACTATTATTAATATACGATGAGAAGGTATATTAATAGAACGACTTCCACTATTTGTGTGATCTGTTTTTATTACCGCAATTTTACTATTACTATCATTACCAGTACTCGCTGGAGCAATTACGATACTTTTAACTGACCGAAATTTTAA

>Vigo_Spain_(KC851963)

GTAATTGTTACAGCTCACGCATTTGTTATAATTTTTTTTCTTGTTATACCAGTTATAATTGGAGGATTTGGAAACTGATTAGTTCCTTTAATACTAGGAGCACCAGATATAGCATTCCCACAAATAAATAACATAAGCTTCTGACTCTTACCTCCTTCTCTCACTCTTCTCCTTTCATCTGCAGCTGTTGAAAGAGGTGCAGGTACCGGATGAACCGTTTACCCACCTCTTTCAAGAAATTTAGCTCATATAGGACCCTCTGTTGATCTAGCAATTTTCTCACTTCACTTAGCAGGTATTTCATCAATTCTTGGAGCCATCAATTTTATTACAACTATTATTAATATACGATGAGAAGGTATATTAATAGAACGACTTCCACTATTTGTGTGATCTGTTTTTATTACCGCAATTTTACTATTACTATCATTACCAGTACTCGCTGGAGCAATTACTATACTTTTAACTGACCGAAATTTTAA

>Vigo_Spain_(KC851964)

GTAATTGTTACAGCTCACGCATTTGTTATAATTTTTTTTCTTGTTATACCAGTTATAATTGGAGGATTTGGAAACTGATTAGTTCCTTTAATACTAGGAGCACCAGATATAGCATTCCCACGAATAAATAACATAAGCTTCTGACTCTTACCTCCTTCTCTCACTCTTCTCCTTTCATCTGCAGCTGTTGAAAGTGGTGCAGGTACCGGATGAACCGTTTACCCACCTCTTTCAAGAAATTTAGCTCATATAGGACCCTCTGTTGATCTAGCAATTTTCTCACTTCACTTAGCAGGTATTTCATCAATTCTTGGAGCCATCAATTTTATTACAACTATTATTAATATACGATGAGAAGGTATATTAATAGAACGACTTCCACTATTTGTGTGATCTGTTTTTATTACCGCAATTTTACTATTACTATCATTACCAGTACTCGCTGGAGCAATTACTATACTTTTAACTGACCGAAATTTTAA

>Barcelona_Spain_(KC851965)

GTAATTGTTACAGCTCACGCATTTGTTATAATTTTTTTTCTTGTTATACCAGTTATAATTGGAGGATTTGGAAACTGATTAGTTCCTTTAATACTAGGAGCACCAGATATAGCATTCCCACGAATAAATAACATAAGCTTCTGACTCTTACCTCCTTCTCTCACTCTTCTCCTTTCATCTGCAGCTGTTGAAAGAGGTGCAGGTACCGGATGAACCGTTTACCCACCTCTTTCAAGAAATTTAGCTCATATAGGACCCTCTGTTGATCTAGCAATTTTCTCACTTCACTTAGCAGGTATTTCATCAATTCTTGGAGCCATCAACTTTATTACAACTATTATTAATATACGATGAGAAGGTATATTAATAGAACGACTTCCACTATTTGTGTGATCTGTTTTTATTACCGCAATTTTACTATTATTATCATTACCAGTACTCGCTGGAGCAATTACTATACTTTTAACTGACCGAAATTTTAA

>Barcelona_Spain_(KC851966)

GTAATTGTTACAGCTCACGCATTTGTTATAATTTTTTTTCTTGTTATACCAGTTATAATTGGAGGATTTGGAAACTGATTAGTTCCTTTAATACTAGGAGCACCAGATATAGCATTCCCACGAATAAATAATATAAGCTTCTGACTCTTACCTCCTTCTCTCACTCTTCTCCTTTCATCTGCAGCTGTTGAAAGTGGTGCAGGTACCGGATGAACCGTTTACCCACCTCTTTCAAGAAATTTAGCTCATATAGGACCCTCTGTTGATCTAGCAATTTTCTCACTTCACTTAGCAGGTATTTCATCAATTCTTGGAGCCATCAATTTTATTACAACTATTATTAATATACGATGAGAAGGTATATTAATAGAACGACTTCCACTATTTGTGTGATCTGTTTTTATTACCGCAATTTTACTATTACTATCATTACCAGTACTCGCTGGAGCAATTACTATACTTTTAACTGACCGAAATTTTAA

>Barcelona_Spain_(KC851967)

GTAATTGTTACAGCTCACGCATTTGTTATAATTTTTTTTCTTGTTATACCAGTTATAATTGGAGGATTTGGAAACTGATTAGTTCCTTTAATACTAGGAGCACCAGATATAGCATTCCCACGAATAAATAACATAAGCTTCTGACTCTTACCTCCTTCTCTCACTCTTCTCCTTTCATCTGCAGCTGTTGAAAGTGGTGCAGGTACCGGATGAACCGTTTACCCACCTCTTTCAAGAAATTTAGCTCATATAGGACCCTCTGTTGATCTAGCAATTTTCTCACTTCACTTAGCAGGTATTTCATCAATTCTTGGAGCCATCAATTTTATTACAACTATTATTAATATACGATGAGAAGGTATATTAATAGAACGACTTCCACTATTTGTGTGATCTGTTTTTATTACCGCAATTTTACTATTACTATCATTACCAGTACTCGCTGGAGCAATTACTATACTTTTAACTGACCGAAATTTTAA

>Barcelona_Spain_(KC851968)

GTAATTGTTACAGCTCACGCATTTGTTATAATTTTTTTTCTTGTTATACCAGTTATAATTGGAGGATTTGGAAACTGATTAGTTCCTTTAATACTAGGAACACCAGATATAGCATTCCCACGAATAAATAACATAAGCTTCTGACTCTTACCTCCTTCTCTCACTCTTCTCCTTTCATCTGCAGCTGTTGAAAGTGGTGCAGGTACCGGATGAACCGTTTACCCACCTCTTTCAAGAAATTTAGCTCATATAGGACCCTCTGTTGATCTAGCAATTTTCTCACTTCACTTAGCAGGTATTTCATCAATTCTTGGAGCCATCAATTTTATTACAACTATTATTAATATACGATGAGAAGGTATATTAATAGAACGACTTCCACTATTTGTGTGATCTGTTTTTATTACCGCAATTTTACTATTACTATCATTACCAGTACTCGCTGGAGCAATTACTATACTTTTAACTGACCGAAATTTTAA

>Barcelona_Spain_(KC851969)

GTAATTGTTACAGCTCACGCATTTGTTATAATTTTTTTTCTTGTTATACCAGTTATAATTGGAGGATTTGGAAACTGATTAGTTCCTTTAATACTAGGAGCACCAGATATAGCATTCCCACGAATAAATAACATAAGCTTCTGACTCTTACCTCCTTCTCTCACTCTTCTCCTTTCATCTGCAGCTGTTGAAAGAGGTGCAGGTACCGGATGAACCGTTTACCCACCTCTTTCAAGAAATTTAGCTCATATAGGACCCTCTGTTGATCTAGCAATTTTCTCACTTCACTTAGCAGGTATTTCATCAATTCTTGGAGCCATCAACTTTATTACAACTATTATTAATATACGATGAGAAGGTATATTAATAGAACGACTTCCACTATTTGTGTGATCTGTTTTTATTACCGCAATTTTACTATTATTATCATTACCAGTACTCGCTGGAGCAATTACGATACTTTTAACTGACCGAAATTTTAA

>Barcelona_Spain_(KC851970)

GTAATTGTTACAGCTCACGCATTTGTTATAATTTTTTTTCTTGTTATACCAGTTATAATTGGAGGATTTGGAAACTGATTAGTTCCTTTAATACTAGGAGCACCAGATATAGCATTCCCACGAATAAATAACATAAGCTTCTGACTCTTACCTCCTTCTCTCACTCTTCTCCTTTCATCTGCAGCTGTTGAAAGAGGTGCAGGTACCGGATGAACCGTTTACCCACCTCTTTCAAGAAATTTAGCTCATATAGGACCCTCTGTTGATCTAGCAATTTTCTCACTTCACTTAGCAGGTATTTCATCAATTCTTGGAGCCATCAACTTTATTACAACTATTATTAATATACGATGAGAAGGTATATTAATAGAACGACTTCCACTATTTGTGTGATCTGTTTTTATTACCGCAATTTTACTATTATTATCATTACCAGTACTCGCTGGAGCAATTACTATACTTTTAACTGACCGAAATTTTAA

>Livorno_Italy_(KC851973)

GTAATTGTTACAGCTCACGCATTTGTTATAATTTTTTTTCTTGTTATACCAGTTATAATTGGAGGATTTGGAAACTGATTAGTTCCTTTAATACTAGGAGCACCAGATATAGCATTCCCACAAATAAATAACATAAGCTTCTGACTCTTACCTCCTTCTCTCACTCTTCTCCTTTCATCTGCAGCTGTTGAAAGAGGTGCAGGTACCGGATGAACCGTTTACCCACCTCTTTCAAGAAATTTAGCTCATATAGGACCCTCTGTTGATCTAGCAATTTTCTCACTTCACTTAGCAGGTATTTCATCAATTCTTGGAGCCATCAACTTTATTACAACTATTATTAATATACGATGAGAAGGTATATTAATAGAACGACTTCCACTATTTGTGTGATCTGTTTTTATTACCGCAATTTTACTATTATTATCATTACCAGTACTCGCTGGAGCAATTACTATACTTTTAACTGACCGAAATTTTAA

>Livorno_Italy_(KC851974)

GTAATTGTTACAGCTCACGCATTTGTTATAATTTTTTTTCTTGTTATACCAGTTATAATTGGAGGATTTGGAAACTGATTAGTTCCTTTAATACTAGGAGCACCAGATATAGCATTCCCACAAATAAATAACATAAGCTTCTGACTCTTACCTCCTTCTCTCACTCTTCTCCTTTCATCTGCAGCTGTTGAAAGAGGTGCAGGTACCGGATGAACCGTTTACCCACCTCTTTCAAGAAATTTAGCTCATATAGGACCCTCTGTTGATCTAGCAATTTTCTCACTTCACTTAGCAGGTATTTCATCAATTCTTGGAGCCATCAATTTTATTACAACTATTATTAATATACGATGAGAAGGTATATTAATAGAACGACTTCCACTATTTGTGTGATCTGTTTTTATTACCGCAATTTTACTATTACTATCATTACCAGTACTCGCTGGAGCAATTAGTATACTTTTAACTGACCGAAATTTTAA

>Livorno_Italy_(KC851975)

GTAATTGTTACAGCTCACGCATTTGTTATAATTTTTTTTCTTGTTATACCAGTTATAATTGGAGGATTTGGAAACTGATTAGTTCCTTTAATACTAGGAGCACCAGATATAGCATTCCCACGAATAAATAACATAAGCTTCTGACTCTTACCTCCTTCTCTCACTCTTCTCCTTTCATCTGCAGCTGTTGAAAGAGGTGCAGGTACCGGATGAACCGTTTACCCACCTCTTTCAAGAAATTTAGCTCATATAGGACCCTCTGTTGATCTAGCAATTTTCTCACTTCACTTAGCAGGTATTTCATCAATTCTTGGAGCCATCAACTTTATTACAACTATTATTAATATACGATGAGAAGGTATATTAATAGAACGACTTCCACTATTTGTGTGATCTGTTTTTATTACCGCAATTTTACTATTATTATCATTACCAGTACTCGCTGGAGCAATTAGTATACTTTTAACTGACCGAAATTTTAA

>Livorno_Italy_(KC851976)

GTAATTGTTACAGCTCACGCATTTGTTATAATTTTTTTTCTTGTTATACCAGTTATAATTGGAGGATTTGGAAACTGATTAGTTCCTTTAATACTAGGAGCACCAGATATAGCATTCCCACGAATAAATAACATAAGCTTCTGACTCTTACCTCCTTCTCTCACTCTTCTCCTTTCATCTGCAGCTGTTGAAAGTGGTGCAGGAACCGGATGAACCGTTTACCCACCTCTTTCAAGAAATTTAGCTCATATAGGACCCTCTGTTGATCTAGCAATTTTCTCACTTCACTTAGCAGGTATTTCATCAATTCTTGGAGCCATCAATTTTATTACAACTATTATTAATATACGATGAGAAGGTATATTAATAGAACGACTTCCACTATTTGTGTGATCTGTTTTTATTACCGCAATTTTACTATTACTATCATTACCAGTACTCGCTGGAGCAATTACTATACTTTTAACTGACCGAAATTTTAA

>Livorno_Italy_(KC851977)

GTAATTGTTACAGCTCACGCATTTGTTATAATTTTTTTTCTTGTTATACCAGTTATAATTGGAGGATTTGGAAACTGATTAGTTCCTTTAATACTAGGAGCACCAGATATAGCATTCCCACGAATAAATAACATAAGCTTCTGACTCTTACCTCCTTCTCTCACTCTTCTCCTTTCATCTGCAGCTGTTGAAAGAGGTGCAGGTACCGGATGAACCGTTTACCCACCTCTTTCAAGAAATTTAGCTCATATAGGACCCTCTGTTGATCTAGCAATTTTCTCACTTCACTTAGCAGGTATTTCATCAATTCTTGGAGCCATCAACTTTATTACAACTATTATTAATATACGATGAGAAGGTATATTAATAGAACGACTTCCACTATTTGTGTGATCTGTTTTTATTACCGCAATTTTACTATTATTATCATTACCAGTACTCGCTGGAGCAATTAGTATACTTTTAACTGACCGAAATTTTAA

>Livorno_Italy_(KC851978)

GTAATTGTTACAGCTCACGCATTTGTTATAATTTTTTTTCTTGTTATACCAGTTATAATTGGAGGATTTGGAAACTGATTAGTTCCTTTAATACTAGGAGCACCAGATATAGCATTCCCACAAATAAATAACATAAGCTTCTGACTCTTACCTCCTTCTCTCACTCTTCTCCTTTCATCTGCAGCTGTTGAAAGTGGTGCAGGTACCGGATGAACCGTTTACCCACCTCTTTCAAGAAATTTAGCTCATATAGGACCCTCTGTTGATCTAGCAATTTTCTCACTTCACTTAGCAGGTATTTCATCAATTCTTGGAGCCATCAATTTTATTACAACTATTATTAATATACGATGAGAAGGTATATTAATAGAACGACTTCCACTATTTGTGTGATCTGTTTTTATTACCGCAATTTTACTATTACTATCATTACCAGTACTCGCTGGAGCAATTAGTATACTTTTAACTGACCGAAATTTTAA

>Livorno_Italy_(KC851979)

GTAATTGTTACAGCTCACGCATTTGTTATAATTTTTTTTCTTGTTATACCAGTTATAATTGGAGGATTTGGAAACTGATTAGTTCCTTTAATACTAGGAGCACCAGATATAGCATTCCCACAAATAAATAACATAAGCTTCTGACTCTTACCTCCTTCTCTCACTCTTCTCCTTTCATCTGCAGCTGTTGAAAGAGGTGCAGGTACCGGATGAACCGTTTACCCACCTCTTTCAAGAAATTTAGCTCATATAGGACCCTCTGTTGATCTAGCAATTTTCTCACTTCACTTAGCAGGTATTTCATCAATTCTTGGAGCCATCAACTTTATTACAACTATTATTAATATACGATGAGAAGGTATATTAATAGAACGACTTCCACTATTTGTGTGATCTGTTTTTATTACCGCAATTTTACTATTATTATCATTACCAGTACTCGCTGGAGCAATTACTATACTTTTAACTGACCGAAATTTTAA

>Porto_Santo_Stefano_Italy_(KC851980)

GTAATTGTTACAGCTCACGCATTTGTTATAATTTTTTTTCTTGTTATACCAGTTATAATTGGAGGATTTGGAAACTGATTAGTTCCTTTAATACTAGGAGCACCAGATATAGCATTCCCACGAATAAATAACATAAGCTTCTGACTCTTACCTCCTTCTCTCACTCTTCTCCTTTCATCTGCAGCTGTTGAAAGTGGTGCAGGTACCGGATGAACCGTTTACCCACCTCTTTCAAGAAATTTAGCTCATATAGGACCCTCTGTTGATCTAGCAATTTTCTCACTTCACTTAGCAGGTATTTCATCAATTCTTGGAGCCATCAATTTTATTACAACTATTATTAATATACGATGAGAAGGTATATTAATAGAACGACTTCCACTATTTGTGTGATCTGTTTTTATTACCGCAATTTTACTATTACTATCATTACCAGTACTCGCTGGAGCAATTAGTATACTTTTAACTGACCGAAATTTTAA

>Porto_Santo_Stefano_Italy_(KC851981)

GTAATTGTTACAGCTCACGCATTTGTTATAATTTTTTTTCTTGTTATACCAGTTATAATTGGAGGATTTGGAAACTGATTAGTTCCTTTAATACTAGGAGCACCAGATATAGCATTCCCACAAATAAATAACATAAGCTTCTGACTCTTACCTCCTTCTCTCACTCTTCTCCTTTCATCTGCAGCTGTTGAAAGAGGTGCAGGTACCGGATGAACCGTTTACCCACCTCTTTCAAGAAATTTAGCTCATATAGGACCCTCTGTTGATCTAGCAATTTTCTCACTTCACTTAGCAGGTATTTCATCAATTCTTGGAGCCATCAACTTTATTACAACTATTATTAATATACGATGAGAAGGTATATTAATAGAACGACTTCCACTATTTGTGTGATCTGTTTTTATTACCGCAATTTTACTATTATTATCATTACCAGTACTCGCTGGAGCAATTAGTATACTTTTAACTGACCGAAATTTTAA

>Porto_Santo_Stefano_Italy_(KC851982)

GTAATTGTTACAGCTCACGCATTTGTTATAATTTTTTTTCTTGTTATACCAGTTATAATTGGAGGATTTGGAAACTGATTAGTTCCTTTAATACTAGGAGCACCAGATATAGCATTCCCACAAATAAATAACATAAGCTTCTGACTCTTACCTCCTTCTCTCACTCTTCTCCTTTCATCTGCAGCTGTTGAAAGAGGTGGAGGTACCGGATGAACCGTTTACCCACCTCTTTCAAGAAATTTAGCTCATATAGGACCCTCTGTTGATCTAGCAATTTTCTCACTTCACTTAGCAGGTATTTCATCAATTCTTGGAGCCATCAATTTTATTACAACTATTATTAATATACGATGAGAAGGTATATTAATAGAACGACTTCCACTATTTGTGTGATCTGTTTTTATTACCGCAATTTTACTATTACTATCATTACCAGTACTCGCTGGAGCAATTACTATACTTTTAACTGACCGAAATTTTAA

>Porto_Santo_Stefano_Italy_(KC851983)

GTAATTGTTACAGCTCACGCATTTGTTATAATTTTTTTTCTTGTTATACCAGTTATAATTGGAGGATTTGGAAACTGATTAGTTCCTTTAATACTAGGAGCACCAGATATAGCATTCCCACGAATAAATAACATAAGCTTCTGACTCTTACCTCCTTCTCTCACTCTTCTCCTTTCATCTGCAGCTGTTGAAAGAGGTGCAGGTACCGGATGAACCGTTTACCCACCTCTTTCAAGAAATTTAGCTCATATAGGACCCTCTGTTGATCTAGCAATTTTCTCACTTCACTTAGCAGGTATTTCATCAATTCTTGGAGCCATCAACTTTATTACAACTATTATAAATATACGATGAGAAGGTATATTAATAGAGCGACTTCCACAATTTGTGTGATCTGTTTTTATTACCGCAATTTTACTATTATTATCATTACCAGTACTCGCTGGAGCAATTAGTATACTTTTAACTGACCGAAATTTTAA

>Porto_Santo_Stefano_Italy_(KC851984)

GTAATTGTTACAGCTCACGCATTTGTTATAATTTTTTTTCTTGTTATACCAGTTATAATTGGAGGATTTGGAAACTGATTAGTTCCTTTAATACTAGGAGCACCAGATATAGCATTCCCACGAATAAATAACATAAGCTTCTGACTCTTACCTCCTTCTCTCACTCTTCTCCTTTCATCTGCAGCTGTTGAAAGTGGTGCAGGTACCGGATGAACCGTTTACCCACCTCTTTCAAGAAATTTAGCTCATATAGGACCCTCTGTTGATCTAGCAATTTTCTCACTTCACTTAGCAGGTATTTCATCAATTCTTGGAGCCATCAATTTTATTACAACTATTATTAATATACGATGAGAAGGTATATTAATAGAACGACTTCCACTATTTGTGTGATCTGTTTTTATTACCGCAATTTTACTATTACTATCATTACCAGTACTCGCTGGAGCAATTAGTATACTTTTAACTGACCGAAATTTTAA

>Olbia_Italy_(KC851985)

GTAATTGTTACAGCTCACGCATTTGTTATAATTTTTTTTCTTGTTATACCAGTTATAATTGGAGGATTTGGAAACTGATTAGTTCCTTTAATACTAGGAGCACCAGATATAGCATTCCCACGAATAAATAACATAAGCTTCTGACTCTTACCTCCTTCTCTCACTCTTCTCCTTTCATCTGCAGCTGTTGAAAGAGGTGCAGGTACCGGATGAACCGTTTACCCACCTCTTTCAAGAAATTTAGCTCATATAGGACCCTCTGTTGATCTAGCAATTTTCTCACTTCACTTAGCAGGTATTTCATCAATTCTTGGAGCCATCAACTTTATTACAACTATTATTAATATACGATGAGAAGGTATATTAATAGAACGACTTCCACTATTTGTGTGATCTGTTTTTATTACCGCAATTTTACTATTATTATCATTACCAGTACTCGCTGGAGCAATTACTATACTTTTAACTGACCGAAATTTTAA

>Porto_Torres_Italy_(KC851986)

GTAATTGTTACAGCTCACGCATTTGTTATAATTTTTTTTCTTGTTATACCAGTTATAATTGGAGGATTTGGGAACTGATTAGTTCCTTTAATACTAGGAGCACCAGATATAGCATTCCCACGAATAAATAACATAAGCTTCTGACTCTTACCTCCTTCTCTCACTCTTCTCCTTTCATCTGCAGCTGTTGAAAGAGGTGCAGGTACCGGATGAACCGTTTACCCACCTCTTTCAAGAAATTTAGCTCATATAGGACCCTCTGTTGATCTAGCAATTTTCTCACTTCACTTAGCAGGTATTTCATCAATTCTTGGAGCCATCAACTTTATTACAACTATTATTAATATACGATGAGAAGGTATATTAATAGAACGACTTCCACTATTTGTGTGATCTGTTTTTATTACCGCAATTTTACTATTATTATCATTACCAGTACTCGCTGGAGCAATTACTATACTTTTAACTGACCGAAATTTTAA

>Alghero_Italy_(KC851987)

GTAATTGTTACAGCTCACGCATTTGTTATAATTTTTTTTCTTGTTATACCAGTTATAATTGGAGGATTTGGAAACTGATTAGTTCCTTTAATACTAGGAGCACCAGATATAGCATTCCCACGAATAAATAACATAAGCTTCTGACTCTTACCTCCTTCTCTCACTCTTCTCCTTTCATCTGCAGCTGTTGAAAGTGGTGCAGGTACCGGATGAACCGTTTACCCACCTCTTTCAAGAAATTTAGCTCATATAGGACCCTCTGTTGATCTAGCAATTTTCTCACTTCACTTAGCAGGTATTTCATCAATTCTTGGAGCCATCAATTTTATTACAACTATTATTAATATACGATGAGAAGGTATATTAATAGAACGACTTCCACTATTTGTGTGATCTGTTTTTATTACCGCAATTTTACTATTACTATCATTACCAGTACTCGCTGGAGCAATTACTATACTTTTAACTGACCGAAATTTTAA

>Alghero_Italy_(KC851988)

GTAATTGTTACAGCTCACGCATTTGTTATAATTTTTTTTCTTGTTATACCAGTTATAATTGGAGGATTTGGAAACTGATTAGTTCCTTTAATACTAGGAGCACCAGATATAGCATTCCCACGAATAAATAACATAAGCTTCTGACTCTTACCTCCTTCTCTCACTCTTCTCCTTTCATCTGCAGCTGTTGAAAGAGGTGCAGGTACCGGATGAACCGTTTACCCACCTCTTTCAAGAAATTTAGCTCATATAGGACCCTCTGTTGATCTAGCAATTTTCTCACTTCACTTAGCAGGTATTTCATCAATTCTTGGAGCCATCAACTTTATTACAACTATTATTAATATACGATGAGAAGGTATATTAATAGAACGACTTCCACTATTTGTGTGATCTGTTTTTATTACCGCAATTTTACTATTATTATCATTACCAGTACTCGCTGGAGCAATTACTATACTTTTAACTGACCGAAATTTTAA

>Alghero_Italy_(KC851989)

GTAATTGTTACAGCTCACGCATTTGTTATAATTTTTTTTCTTGTTATACCAGTTATAATTGGAGGATTTGGAAACTGATTAGTTCCTTTAATACTAGGAGCACCAGATATAGCATTCCCACGAATAAATAACATAAGCTTCTGACTCTTACCTCCTTCTCTCACTCTTCTCCTTTCATCTGCAGCTGTTGAAAGAGGTGCAGGTACCGGATGAACCGTTTACCCACCTCTTTCAAGAAATTTAGCTCATATAGGACCCTCTGTTGATCTAGCAATTTTCTCACTTCACTTAGCAGGTATTTCATCAATTCTTGGAGCCATCAACTTTATTACAACTATTATTAATATACGATGAGAAGGTATATTAATAGAACGACTTCCACTATTTGTGTGATCTGTTTTTATTACCGCAATTTTACTATTATTATCATTACCAGTACTCGCTGGAGCAATCACTATACTTTTAACTGACCGAAATTTTAA

>Alghero_Italy_(KC851990)

GTAATTGTTACAGCTCACGCATTTGTTATAATTTTTTTTCTTGTTATACCAGTTATAATTGGAGGATCTGGAAACAGATTAGTTCCTTTAATACTAGGAGCACCAGATATAGCATTCCCACAAATAAATAACATAAGCTTCTGACTCTTACCTCCTTCTCTCACTCTTCTCCTTTCATCTGCAGCTGTTGAAAGAGGTGCAGGTACCGGATGAACCGTTTACCCACCTCTTTCAAGAAATTTAGCTCATATAGGACCCTCTGTTGATCTAGCAATATTCTCACTTCACTTATCAGGTATTTCATCAATTCTTGGAGCCATCAACTTTATTACAACTATTATTAATATACGATGAGAAGGTATATTAATAGAACGACTTCCACTATTTGTGTGATCTGTTTTTATTACCGCAATTTTACTATTACTATCATTACCAGTACTCGCTGGAGCAATTACTATACTTTTAACTGACCGAAATTTTAA

>Alghero_Italy_(KC851991)

GTAATTGTTACAGCTCACGCATTTGTTATAATTTTTTTTCTTGTTATACCAGTTATAATTGGAGGATCTGGAAACTGATTAGTTCCTTTAATACTAGGAGCACCAGATATAGCATTCCCACGAATAAATAACATAAGCTTCTGACTCTTACCTCCTTCTCTCACTCTTCTCCTTTCATCTGCAGCTGTTGAAAGAGGTGCAGGTACCGGATGAACCGTTTACCCACCTCTTTCAAGAAATTTAGCTCATATAGGACCCTCTGTTGATCTAGCAATTTTCTCACTTCACTTAGCAGGTATTTCATCAATTCTTGGAGCCATCAACTTTATTACAACTATTATTAATATACGATGAGAAGGTATATTAATAGAACGACTTCCACTATTTGTGTGATCTGTTTTTATTACCGCAATTTTACTATTATTATCATTACCAGTACTCGCTGGAGCAATTACTATACTTTTAACTGACCGAAATTTTAA

>Alghero_Italy_(KC851992)

GTAATTGTTACAGCTCACGCATTTGTTATAATTTTTTTTCTTGTTATACCAGTTATAATTGGAGGATCTGGAAACCGATTAGTTCCTTTAATACTAGGAGCACCAGATATAGCATTCCCACGAATAAATAACATAAGCTTCTGACTCTTACCTCCTTCTCTCACTCTTCTCCTTTCATCTGCAGCTGTTGAAAGAGGTGCAGGTACCGGATGAACCGTTTACCCACCTCTTTCAAGAAATTTAGCTCATATAGGACCCTCTGTTGATCTAGCAATTTTCTCACTTCACTTAGCAGGTATTTCATCAATTCTTGGAGCCATCAACTTTATTACAACTATTATTAATATACGATGAGAAGGTATATTAATAGAACGACTTCCACTATTTGTGTGATCTGTTTTTATTACCGCAATTTTACTATTATTATCATTACCAGTACTCGCTGGAGCAATTACTATACTTTTAACTGACCGAAATTTTAA

>Alghero_Italy_(KC851993)

GTAATTGTTACAGCTCACGCATTTGTTATAATTTTTTTTCTTGTTATACCAGTTATAATTGGAGGATCTGGAAACTGATTAGTTCCTTTAATACTAGGAGCACCAGATATAGCATTCCCACGAATAAATAACATAAGCTTCTGACTCTTACCTCCTTCTCTCACTCTTCTCCTTTCATCTGCAGCTGTTGAAAGAGGTGCAGGTACCGGATGAACCGTTTACCCACCTCTTTCAAGAAATTTAGCTCATATAGGACCCTCTGTTGATCTAGCAATTTTCTCACTTCACTTAGCAGGTATTTCATCAATTCTTGGAGCCATCAACTTTATTACAACTATTATTAATATACGATGAGAAGGTATATTAATAGAACGACTTCCACTATTTGTGTGATCTGTTTTTATTACCGCAATTTTACTATTATTATCATTACCAGTACTCGCTGGAGCAATTACTATACTTTTAACTGACCGAAATTTTAA

>Napoli_Italy_(KC851994)

GTAATTGTTACAGCTCACGCATTTGTTATAATTTTTTTTCTTGTTATACCAGTTATAATTGGAGGATTTGGAAACTGATTAGTTCCTTTAATACTAGGAGCACCAGATATAGCATTCCCACAAATAAATAACATAAGCTTCTGACTCTTACCTCCTTCTCTCACTCTTCTCCTTTCATCTGCAGCTGTTGAAAGAGGTGCAGGTACCGGATGAACCGTTTACCCACCTCTTTCAAGAAATTTAGCTCATATAGGACCCTCTGTTGATCTAGCAATTTTCTCACTTCACTTAGCAGGTATTTCATCAATTCTTGGAGCCATCAACTTTATTACAACTATTATTAATATACGATGAGAAGGTATATTAATAGAACGACTTCCACTATTTGTGTGATCTGTTTTTATTACCGCAATTTTACTATTATTATCATTACCAGTACTCGCTGGAGCAATTACTATACTTTTAACTGACCGAAATTTTAA

>Napoli_Italy_(KC851995)

GTAATTGTTACAGCTCACGCATTTGTTATAATTTTTTTTCTTGTTATACCAGTTATAATTGGAGGATTTGGAAACTGATTAGTTCCTTTAATACTAGGAGCACCAGATATAGCATTCCCACAAATAAATAACATAAGCTTCTGACTCTTACCTCCTTCTCTCACTCTTCTCCTTTCATCTGCAGCTGTTGAAAGTGGTGCAGGTACCGGATGAACCGTTTACCCACCTCTTTCAAGAAATTTAGCTCATATAGGACCCTCTGTTGATCTAGCAATTTTCTCACTTCACTTAGCAGGTATTTCATCAATTCTTGGAGCCATCAATTTTATTACAACTATTATTAATATACGATGAGAAGGTATATTAATAGAACGACTTCCACTATTTGTGTGATCTGTTTTTATTACCGCAATTTTACTATTACTATCATTACCAGTACTCGCTGGAGCAATTACTATACTTTTAACTGACCGAAATTTTAA

>Napoli_Italy_(KC851996)

GTAATTGTTACAGCTCACGCATTTGTTATAATTTTTTTTCTTGTTATACCAGTTATAATTGGAGGATTTGGAAACTGATTAGTTCCTTTAATACTAGGAGCACCAGATATAGCATTCCCACGAATAAATAACATAAGCTTCTGACTCTTACCTCCTTCTCTCACTCGTCTCCTTTCATCTGCAGCTGTTGAAAGTGGTGCAGGTACCGGATGAACCGTTTACCCACCTCTTTCAAGAAATTTAGCTCATATAGGACCCTCTGTTGATCTAGCAATTTTCTCACTTCACTTAGCAGGTATTTCATCAATTCTTGGAGCCATCAATTTTATTACAACTATTATTAATATACGATGAGAAGGTATATTAATAGAACGACTTCCACTATTTGTGTGATCTGTTTTTATTACCGCAATTTTACTATTACTATCATTACCAGTACTCGCTGGAGCAATTAGTATACTTTTAACTGACCGAAATTTTAA

>Napoli_Italy_(KC851997)

GTAATTGTTACAGCTCACGCATTTGTTATAATTTTTTTTCTTGTTATACCAGTTATAATTGGAGGATTTGGAAACTGATTAGTTCCTTTAATACTAGGAGCACCAGATATAGCATTCCCACAAATAAATAACATAAGCTTCTGACTCTTACCTCCTTCTCTCACTCTTCTCCTTTCATCTGCAGCTGTTGAAAGAGGTGCAGGTACCGGATGAACCGTTTACCCACCTCTTTCAAGAAATTTAGCTCATATAGGACCCTCTGTTGATCTAGCAATTTTCTCACTTCACTTAGCAGGTATTTCATCAATTCTTGGAGCCATCAACTTTATTACAACTATTATTAATATACGATGAGAAGGTATATTAATAGAACGACTTCCACTATTTGTGTGATCTGTTTTTATTACCGCAATTTTACTATTATTATCATTACCAGTACTCGCTGGAGCAATTAGTATACTTTTAACTGACCGAAATTTTAA

>Crete_Greece_(KC852003)

GTAATTGTTACAGCTCACGCATTTGTTATAATTTTTTTTCTTGTTATACCAGTTATAATTGGAGGATTTGGAAACTGATTAGTTCCTTTAATACTAGGAGCACCAGATATAGCATTCCCACGAATAAATAACATAAGCTTCTGACTCTTACCTCCTTCTCTCACTCTTCTCCTTTCATCTGCAGCTGTTGAAAGAGGTGCAGGTACCGGATGAACCGTTTACCCACCTCTTTCAAGAAATTTAGCTCATATAGGACCCTCTGTTGATCTAGCAATTTTCTCACTTCACTTAGCAGGTATTTCATCAATTCTTGGAGCCATCAACTTTATTACAACTATTATTAATATACGATGAGAAGGTATATTAATAGAACGGCTTCCACTATTTGTGTGATCTGTTTTTATTACCGCAATTTTACTATTATTATCATTACCAGTACTCGCTGGAGCAATTACTATACTTTTAACTGACCGAAATTTTAA

>Crete_Greece_(KC852004)

GTAATTGTTACAGCTCACGCATTTGTTATAATTTTTTTTCTTGTTATACCAGTTATAATTGGAGGATTTGGAAACTGATTAGTTCCTTTAATACTAGGAGCACCAGATATAGCATTCCCACGAATAAATAACATAAGCTTCTGACTCTTACCTCCTTCTCTCACTCTTCTCCTTTCATCTGCAGCTGTTGAAAGAGGTGCAGGTACCGGATGAACCGTTTACCCACCTCTTTCAAGAAATTTAGCTCATATAGGACCCTCTGTTGATCTAGCAATTTTCTCACTTCACTTAGCAGGTATTTCATCAATTCTTGGAGCCATCAACTTTATTACAACTATTATTAATATACGATGAGAAGGTATATTAATAGAACGACTTCCACTATTTGTGTGATCTGTTTTTATTACCGCAATTTTACTATTATTATCGTTACCAGTACTCGCTGGAGCAATTACTATACTTTTAACTGACCGAAATTTTAA

>Crete_Greece_(KC852005)

GTAATTGTTACAGCTCACGCATTTGTTATAATTTTTTTTCTTGTTATACCAGTTATAATTGGAGGATTTGGAAACTGATTAGTTCCTTTAATACTAGGAGCACCAGATATAGCATTCCCACGAATAAATAACATAAGCTTCTGACTCTTACCTCCTTCTCTCACTCTTCTCCTTTCATCTGCAGCTGTTGAAAGAGGTGCAGGTACCGGATGAACCGTTTACCCACCTCTTTCAAGAAATTTAGCTCATATAGGACCCTCTGTTGATCTAGCAATTTTTTCACTTCACTTAGCAGGTATTTCATCAATTCTTGGAGCCATCAACTTTATTACAACTATTATTAATATACGATGAGAAGGTATATTAATAGAACGACTTCCACTATTTGTGTGATCTGTTTTTATTACCGCAATTTTACTATTATTATCATTACCAGTACTCGCTGGAGCAATTACTATACTTTTAACTGACCGAAATTTTAA

>Ancona_Italy_(KC852006)

GTAATTGTTACAGCTCACGCATTTGTTATAATTTTTTTTCTTGTTATACCAGTTATAATTGGAGGATTTGGAAACTGATTAGTTCCTTTAATACTAGGAGCACCAGATATAGCATTCCCACGAATAAATAACATAAGCTTCTGACTCTTACCTCCTTCTCTCACTCTTCTCCTTTCATCTGCAGCTGTTGAAAGAGGTGCAGGTACCGGATGAACCGTTTACCCACCTCTTTCAAGAAATTTAGCTCATATAGGACCCTCTGTTGATCTAGCAATTTTCTCACTTCACTTAGCAGGTATTTCATCAATTCTTGGAGCCATCAACTTTATTACAACTATTATTAATATACGATGAGAAGGTATATTAATAGAACGACTTCCACTATTTGTGTGATCTGTTTTTATTACCGCAATTTTACTATTATTATCATTACCAGTACTCGCTGGAGCAATTACTATACTTTTAACTGACCGAAATTTTAA

>Ancona_Italy_(KC852007)

GTAATTGTTACAGCTCACGCATTTGTTATAATTTTTTTTCTTGTTATACCAGTTATAATTGGAGGATTTGGAAACTGATTAGTTCCTTTAATACTAGGAGCACCAGATATAGCATTCCCACGAATAAATAACATAAGCTTCTGACTCTTACCTCCTTCTCTCACTCTTCTCCTTTCATCTGCAGCTGTTGAAAGAGGTGCAGGTACCGGATGAACCGTTTACCCACCTCTTTCAAGAAATTTAGCTCATATAGGACCCTCTGTTGATCTAGCAATTTTCTCACTTCACTTAGCAGGTATTTCATCAATTCTTGGAGCCATCAACTTTATTACAACTATTATTAATATACGATGAGAAGGTATATTAATAGAACGACTTCCACTATTTGTATGATCTGTTTTTATTACCGCAATTTTACTATTATTATCATTACCAGTACTCGCTGGAGCAATTACTATACTTTTAACTGACCGAAATTTTAA

>Ancona_Italy_(KC852008)

GTAATTGTTACAGCTCACGCATTTGTTATAATTTTTTTTCTTGTTATACCAGTTATAATTGGAGGATTTGGAAACTGATTAGTTCCTTTAATACTAGGAGCACCAGATATAGCATTCCCACGAATAAATAACATAAGCTTCTGACTCTTACCTCCTTCTCTCACTCTTCTCCTTTCATCTGCAGCTGTTGAAAGAGGTGTAGGTACCGGATGAACCGTTTACCCACCTCTTTCAAGAAATTTAGCTCATATAGGACCCTCTGTTGATCTAGCAATTTTCTCACTTCACTTAGCAGGTATTTCATCAATTCTTGGAGCCATCAACTTTATTACAACTATTATTAATATACGATGAGAAGGTATATTAATAGAACGACTTCCACTATTTGTGTGATCTGTTTTTATTACCGCAATTTTACTATTATTATCATTACCAGTACTCGCTGGAGCAATTACTATACTTTTAACTGACCGAAATTTTAA

>Cabo_Norte_Brasil_(KF844026)

GTAATTGTTACAGCTCACGCATTTGTTATAATTTTTTTTCTTGTTATACCAGTTATAATTGGAGGATTCGGAAACTGATTAGTTCCTTTAATACTAGGAGCACCAGATATAGCATTCCCACGAATAAATAACATAAGCTTTTGACTCTTACCCCCTTCTCTCACTCTTCTCCTTTCATCTGCAGCAGTTGAAAGAGGTGCAGGTACCGGGTGAACCGTTTACCCTCCTCTTTCAAGAAATTTAGCTCATATAGGACCTTCTGTTGATCTAGCCATTTTCTCACTTCACTTAGCAGGTATTTCATCAATTCTTGGAGCCATCAACTTTATTACAACTATTATTAATATACGATGAGAAGGTATATTAATAGAACGACTTCCACTATTTGTATGATCTGTCTTTATTACCGCAATTTTACTATTACTATCATTACCAGTACTTGCTGGAGCAATTACTATACTTTTAACTGATCGAAATTTTAA

>Bragana_Brasil_(KF844027)

GTAATTGTTACAGCTCACGCATTTGTTATAATTTTTTTTCTTGTTATACCAGTTATAATTGGAGGATTTGGAAACTGATTAGTTCCTTTAATACTAGGAGCACCAGATATAGCATTCCCACGAATAAATAACATAAGCTTCTGACTCTTACCTCCTTCTCTCACTCTTCTCCTTTCATCTGCAGCTGTTGAAAGAGGTGCAGGTACCGGATGAACCGTTTACCCACCTCTTTCAAGAAATTTAGCTCATATAGGACCCTCTGTTGATCTAGCAATTTTCTCACTTCACTTAGCAGGTATTTCATCAATTCTTGGAGCCATCAACTTTATTACAACTATTATTAATATACGATGAGAAGGTATATTAATAGAACGACTTCCACTATTTGTGTGATCTGTTTTTATTACCGCAATTTTACTATTACTATCATTACCAGTACTCGCTGGAGCAATTACTATACTTTTAACTGACCGAAATTTTAA

>Bragana_Brasil_(KF844028)

GTAATTGTTACAGCTCACGCATTTGTTATAATTTTTTTTCTTGTTATACCAGTTATAATTGGAGGATTCGGAAACTGATTAGTTCCTTTAATACTAGGAGCACCAGATATAGCATTCCCACGAATAAATAACATAAGCTTTTGACTCTTACCCCCTTCTCTCACTCTTCTCCTTTCATCTGCAGCAGTTGAAAGAGGTGCAGGTACCGGGTGAACCGTTTACCCTCCTCTTTCAAGAAATTTAGCTCATATAGGACCTTCTGTTGATCTAGCCATTTTCTCACTTCACTTAGCAGGTATTTCATCAATTCTTGGAGCCATCAACTTTATTACAACTATTATTAATATACGATGAGAAGGTATATTAATAGAACGACTTCCACTATTTGTATGATCTGTCTTTATTACCGCAATTTTACTATTACTATCATTACCAGTACTTGCTGGAGCAATTACTATACTTTTAACTGATCGAAATTTTAA

>Bragana_Brasil_(KF844029)

GTAATTGTTACAGCTCACGCATTTGTTATAATTTTTTTTCTTGTTATACCAGTTATAATTGGAGGATTCGGAAACTGATTAGTTCCTTTAATACTAGGAGCACCAGATATAGCATTCCCACGAATAAATAACATAAGCTTTTGACTCTTACCCCCTTCTCTCACTCTTCTCCTTTCATCTGCAGCAGTTGAAAGAGGTGCAGGTACCGGGTGAACCGTTTACCCTCCTCTTTCAAGAAATTTAGCTCATATAGGACCTTCTGTTGATCTAGCCATTTTCTCACTTCACTTAGCAGGTATTTCATCAATTCTTGGAGCCATCAACTTTATTACAACTATTATTAATATACGATGAGAAGGTATATTAATAGAACGACTTCCACTATTTGTATGATCTGTCTTTATTACCGCAATTTTACTATTACTATCATTACCAGTACTTGCTGGAGCAATTACTATACTTTTAACTGATCGAAATTTTAA

>Bragana_Brasil_(KF844030)

GTAATTGTTACAGCTCACGCATTTGTTATAATTTTTTTTCTTGTTATACCAGTTATAATTGGAGGATTCGGAAACTGATTAGTTCCTTTAATACTAGGAGCACCAGATATAGCATTCCCACGAATAAATAACATAAGCTTTTGACTCTTACCCCCTTCTCTCACTCTTCTCCTTTCATCTGCAGCAGTTGAAAGAGGTGCAGGTACCGGATGAACCGTTTACCCACCTCTTTCAAGAAATTTAGCTCATATAGGACCTTCTGTTGATCTAGCCATTTTCTCACTTCACTTAGCAGGTATTTCATCAATTCTTGGAGCCATCAACTTTATTACAACTATTATTAATATACGATGAGAAGGTATATTAATAGAACGACTTCCACTATTTGTGTGATCTGTCTTTATTACCGCAATTTTACTATTACTATCATTACCAGTACTTGCTGGAGCAATTACTATACTTTTAACTGATCGAAATTTTAA

>Salvador_Brasil_(KF844031)

GTAATTGTAACAGCTCACGCATTTGTTATAATTTTTTTTCTTGTTATACCAGTTATAATTGGAGGATTCGGAAACTGATTAGTTCCTTTAATACTAGGAGCACCAGATATAGCATTCCCACGAATAAATAACATAAGCTTTTGACTCTTACCCCCTTCTCTCACTCTTCTCCTTTCATCTGCAGCAGTTGAAAGAGGTGCAGGTACCGGGTGAACCGTTTACCCTCCTCTTTCAAGAAATTTAGCTCATATAGGACCTTCTGTTGATCTAGCCATTTTCTCACTTCACTTAGCAGGTATTTCATCAATTCTTGGAGCCATCAACTTTATTACAACTATTATTAATATACGATGAGAAGGTATATTAATAGAACGACTTCCACTATTTGTATGATCTGTCTTTATTACCGCAATTTTACTATTACTATCATTACCAGTACTTGCTGGAGCAATTACTATACTTTTAACTGATCGAAATTTTAA

>Rio_de_Janeiro_Brasil_(KF844032)

GTAATTGTTACAGCTCACGCATTTGTTATAATTTTTTTTCTTGTTATACCAGTTATAATTGGAGGATTCGGAAACTGATTAGTTCCTTTAATACTAGGAGCACCAGATATAGCATTCCCACGAATAAATAACATAAGCTTTTGACTCTTACCCCCTTCTCTCACTCTTCTCCTTTCATCTGCAGCAGTTGAAAGAGGTGCAGGTACCGGGTGAACCGTTTACCCTCCTCTTTCAAGAAATTTAGCTCATATAGGACCTTCTGTTGATCTAGCCATTTTCTCACTTCACTTAGCAGGTATTTCATCAATTCTTGGAGCCATCAACTTTATTACAACTATTATTAATATACGATGAGAAGGTATATTAATAGAACGACTTCCACTATTTGTATGATCTGTCTTTATTACCGCAATTTTACTATTACTATCATTACCAGTACTTGCTGGAGCAATTACTATACTTTTAACTGATCGAAATTTTAA

>Rio_de_Janeiro_Brasil_(KF844033)

GTAATTGTTACAGCTCACGCATTTGTTATAATTTTTTTTCTTGTTATACCAGTTATAATTGGAGGATTCGGAAACTGATTAGTTCCTTTAATACTAGGAGCACCAGATATAGCATTCCCACGAATAAATAACATAAGCTTTTGACTCTTACCCCCTTCTCTCACTCTTCTCCTTTCATCTGCAGCAGTTGAAAGAGGTGCAGGTACCGGGTGAACCGTTTACCCTCCTCTTTCAAGAAATTTAGCTCATATAGGACCTTCTGTTGATCTAGCCATTTTCTCACTTCACTTAGCAGGTATTTCATCAATTCTTGGAGCCATCAACTTTATTACAACTATTATTAATATACGATGAGAAGGTATATTAATAGAACGACTTCCACTATTTGTATGATCTGTCTTTATTACCGCAATTTTACTATTACTATCATTACCAGTACTTGCTGGAGCAATTACTATACTTTTAACTGATCGAAATTTTAA

>Rio_de_Janeiro_Brasil_(KF844034)

GTAATTGTTACAGCTCACGCATTTGTTATAATTTTTTTTCTTGTTATACCAGTTATAATTGGAGGATTCGGAAACTGATTAGTTCCTTTAATACTAGGAGCACCAGATATAGCATTCCCACGAATAAATAACATAAGCTTTTGACTCTTACCCCCTTCTCTCACTCTTCTCCTTTCATCTGCAGCAGTTGAAAGAGGTGCAGGTACCGGGTGAACCGTTTACCCTCCTCTTTCAAGAAATTTAGCTCATATAGGACCTTCTGTTGATCTAGCCATTTTCTCACTTCACTTAGCAGGTATTTCATCAATTCTTGGAGCCATCAACTTTATTACAACTATTATTAATATACGATGAGAAGGTATATTAATAGAACGACTTCCACTATTTGTATGATCTGTCTTTATTACCGCAATTTTACTATTACTATCATTACCAGTACTTGCTGGAGCAATTACTATACTTTTAACTGATCGAAATTTTAA

>Juria_Brasil_(KF844035)

GTAATTGTTACAGCTCACGCATTTGTTATAATTTTTTTTCTTGTTATACCAGTTATAATTGGAGGATTCGGAAACTGATTAGTTCCTTTAATACTAGGAGCACCAGATATAGCATTCCCACGAATAAATAACATAAGCTTTTGACTCTTACCCCCTTCTCTCACTCTTCTCCTTTCATCTGCAGCAGTTGAAAGAGGTGCAGGTACCGGGTGAACCGTTTACCCTCCTCTTTCAAGAAATTTAGCTCATATAGGACCTTCTGTTGATCTAGCCATTTTCTCACTTCACTTAGCAGGTATTTCATCAATTCTTGGAGCCATCAACTTTATTACAACTATTATTAATATACGATGAGAAGGTATATTAATAGAACGACTTCCACTATTTGTATGATCTGTCTTTATTACCGCAATTTTACTATTACTATCATTACCAGTACTTGCTGGAGCAATTACTATACTTTTAACTGATCGAAATTTTAA

>Juria_Brasil_(KF844036)

GTAATTGTTACAGCTCACGCATTTGTTATAATTTTTTTTCTTGTTATACCAGTTATAATTGGAGGATTCGGAAACTGATTAGTTCCTTTAATACTAGGAGCACCAGATATAGCATTCCCACGAATAAATAACATAAGCTTTTGACTCTTACCCCCTTCTCTCACTCTTCTCCTTTCATCTGCAGCAGTTGAAAGAGGTGCAGGTACCGGGTGAACCGTTTACCCTCCTCTTTCAAGAAATTTAGCTCATATAGGACCTTCTGTTGATCTAGCCATTTTCTCACTTCACTTAGCAGGTATTTCATCAATTCTTGGAGCCATCAACTTTATTACAACTATTATTAATATACGATGAGAAGGTATATTAATAGAACGACTTCCACTATTTGTATGATCTGTCTTTATTACCGCAATTTTACTATTACTATCATTACCAGTACTTGCTGGAGCAATTACTATACTTTTAACTGATCGAAATTTTAA

>Juria_Brasil_(KF844037)

GTAATTGTTACAGCTCACGCATTTGTTATAATTTTTTTTCTTGTTATACCAGTTATAATTGGAGGATTCGGAAACTGATTAGTTCCTTTAATACTAGGAGCACCAGATATAGCATTCCCACGAATAAATAACATAAGCTTTTGACTCTTACCCCCTTCTCTCACTCTTCTCCTTTCATCTGCAGCAGTTGAAAGAGGTGCAGGTACCGGGTGAACCGTTTACCCTCCTCTTTCAAGAAATTTAGCTCATATAGGACCTTCTGTTGATCTAGCCATTTTCTCACTTCACTTAGCAGGTATTTCATCAATTCTTGGAGCCATCAACTTTATTACAACTATTATTAATATACGATGAGAAGGTATATTAATAGAACGACTTCCACTATTTGTATGATCTGTCTTTATTACCGCAATTTTACTATTACTATCATTACCAGTACTTGCTGGAGCAATTACTATACTTTTAACTGATCGAAATTTTAA

>Guaratuba_Brasil_(KF844038)

GTAATTGTTACAGCTCACGCATTTGTTATAATTTTTTTTCTTGTTATACCAGTTATAATTGGAGGATTCGGAAACTGATTAGTTCCTTTAATACTAGGAGCACCAGATATAGCATTCCCACGAATAAATAACATAAGCTTTTGACTCTTACCCCCTTCTCTCACTCTTCTCCTTTCATCTGCAGCAGTTGAAAGAGGTGCAGGTACCGGGTGAACCGTTTACCCTCCTCTTTCAAGAAATTTAGCTCATATAGGACCTTCTGTTGATCTAGCCATTTTCTCACTTCACTTAGCAGGTATTTCATCAATTCTTGGAGCCATCAACTTTATTACAACTATTATTAATATACGATGAGAAGGTATATTAATAGAACGACTTCCACTATTTGTATGATCTGTCTTTATTACCGCAATTTTACTATTACTATCATTACCAGTACTTGCTGGAGCAATTACTATACTTTTAACTGATCGAAATTTTAA

>Guaratuba_Brasil_(KF844039)

GTAATTGTTACAGCTCACGCATTTGTTATAATTTTTTTTCTTGTTATACCAGTTATAATTGGAGGATTCGGAAACTGATTAGTTCCTTTAATACTAGGAGCACCAGATATAGCATTCCCACGAATAAATAACATAAGCTTTTGACTCTTACCCCCTTCTCTCACTCTTCTCCTTTCATCTGCAGCAGTTGAAAGAGGTGCAGGTACCGGGTGAACCGTTTACCCTCCTCTTTCAAGAAATTTAGCTCATATAGGACCTTCTGTTGATCTAGCCATTTTCTCACTTCACTTAGCAGGTATTTCATCAATTCTTGGAGCCATCAACTTTATTACAACTATTATTAATATACGATGAGAAGGTATATTAATAGAACGACTTCCACTATTTGTATGATCTGTCTTTATTACCGCAATTTTACTATTACTATCATTACCAGTACTTGCTGGAGCAATTACTATACTTTTAACTGATCGAAATTTTAA

>Cabo_de_Santa_Marta_Brasil_(KF844040)

GTAATTGTTACAGCTCACGCATTTGTTATAATTTTTTTTCTTGTTATACCAGTTATAATTGGAGGATTCGGAAACTGATTAGTTCCTTTAATACTAGGAGCACCAGATATAGCATTCCCACGAATAAATAACATAAGCTTTTGACTCTTACCCCCTTCTCTCACTCTTCTCCTTTCATCTGCAGCAGTTGAAAGAGGTGCAAGTACCGGGTGAACCGTTTACCCTCCTCTTTCAAGAAATTTAGCTCATATAGGACCTTCTGTTGATCTAGCCATTTTCTCACTTCACTTAGCAGGTATTTCATCAATTCTTGGAGCCATCAACTTTATTACAACTATTATTAATATACGATGAGAAGGTATATTAATAGAACGACTTCCACTATTTGTATGATCTGTCTTTATTACCGCAATTTTACTATTACTATCATTACCAGTACTTGCTGGAGCAATTACTATACTTTTAACTGATCGAAATTTTAA

>Cabo_de_Santa_Marta_Brasil_(KF844041)

GTAATTGTTACAGCTCACGCATTTGTTATAATTTTTTTTCTTGTTATACCAGTTATAATTGGAGGATTCGGAAACTGATTAGTTCCTTTAATACTAGGAGCACCAGATATAGCATTCCCACGAATAAATAACATAAGCTTTTGACTCTTACCCCCTTCTCTCACTCTTCTCCTTTCATCTGCAGCAGTTGAAAGAGGTGCAGGTACCGGGTGAACCGTTTACCCTCCTCTTTCAAGAAATTTAGCTCATATAGGACCTTCTGTTGATCTAGCCATTTTCTCACTTCACTTAGCAGGTATTTCATCAATTCTTGGAGCCATCAACTTTATTACAACTATTATTAATATACGATGAGAAGGTATATTAATAGAACGACTTCCACTATTTGTATGATCTGTCTTTATTACCGCAATTTTACTATTACTATCATTACCAGTACTTGCTGGAGCAATTACTATACTTTTAACTGATCGAAATTTTAA

>Portugal_(KF844042)

GTAATTGTTACAGCTCACGCATTTGTTATAATTTTTTTTCTTGTTATACCAGTTATAATTGGAGGATTTGGAAACTGATTAGTTCCTTTAATACTAGGAGCACCAGATATAGCATTCCCACGAATAAATAACATAAGCTTCTGACTCTTACCTCCTTCTCTCACTCTTCTCCTTTCATCTGCAGCTGTTGAAAGTGGTGCAGGTACCGGATGAACCGTTTACCCACCTCTTTCAAGAAATTTAGCTCATATAGGACCCTCTGTTGATCTAGCAATTTTCTCACTTCACTTAGCAGGTATTTCATCAATTCTTGGAGCCATCAATTTTATTACAACTATTATTAATATACGATGAGAAGGTATATTAATAGAACGACTTCCACTATTTGTGTGATCTGTTTTTATTACCGCAATTTTACTATTACTATCATTACCAGTACTCGCTGGAGCAATTACTATACTTTTAACTGACCGAAATTTTAA

>Portugal_(KF844043)

GTAATTGTTACAGCTCACGCATTTGTTATAATTTTTTTTCTTGTTATACCAGTTATAATTGGAGGATTTGGAAACTGATTAGTTCCTTTAATACTAGGAGCACCAGATATAGCATTCCCACGAATAAATAACATAAGCTTCTGACTCTTACCTCCTTCTCTCACTCTTCTCCTTTCATCTGCAGCTGTTGAAAGTGGTGCAGGTACCGGATGAACCGTTTACCCACCTCTTTCAAGAAATTTAGCTCATATAGGACCCTCTGTTGATCTAGCAATTTTCTCACTTCACTTAGCAGGTATTTCATCAATTCTTGGAGCCATCAATTTTATTACAACTATTATTAATATACGATGAGAAGGTATATTAATAGAACGACTTCCACTATTTGTGTGATCTGTTTTTATTACCGCAATTTTACTATTACTATCATTACCAGTACTCGCTGGAGCAATTACTATACTTTTAACTGACCGAAATTTTAA

>Osaka_Japan_(AB191269)

GTAATTGTTACAGCTCACGCATTTGTTATAATTTTTTTCCTTGTTATACCAGTTATAATCGGAGGATTTGGAAACTGATTAGTTCCTTTAATACTAGGAGCACCAGATATAGCATTCCCACGAATAAATAATATAAGCTTCTGACTCTTACCTCCTTCTCTTACTCTTCTCCTTTCATCTGCAGCAGTTGAAAGAGGTGCAGGTACCGGATGAACCGTTTACCCGCTTCTTTCAAGAAATTTAGCTCATATTGGACCTTCTGTTGATCTTGCCATTTTCTCACTTCACTTTGCAGGTATTTCATCAATCCTTGGAGCCATCAACTTTATTACAACTATTATTAATATACGATGAGAAGGTATATTTATAGAACGACTTCCACTATTTGTATGATCTGTATTTATTACCGCAATTTTACTATTACTATCATTACCAGTACTCGCTGGAGCAATTACTATACTTTTTACTGACCGAAATTTTAA

>Seto_Inland_Sea_Japan_(AB430546)

GTAATTGTTACAGCTCACGCATTTGTTATAATTTTTTTCCTTGTTATACCAGTTATAATCGGAGGATTTGGAAACTGATTAGTTCCTTTAATACTAGGAGCACCAGATATAGCATTCCCACGAATAAATAATATAAGCTTCTGACTCTTACCTCCTTCTCTTACTCTTCTCCTTTCATCTGCAGCAGTTGAAAGAGGCGCAGGTACCGGATGAACCGTTTACCCGCCTCTTTCAAGAAATTTAGCTCATATAGGACCTTCTGTTGATCTAGCCATTTTCTCACTTCACTTAGCAGGTATTTCATCAATCCTTGGAGCCATCAACTTTATTACAACTATTATTAATATACGATGAGAAGGTATATTAATAGAACGACTTCCACTATTTGTATGATCTGTATTTATTACCGCAATTTTACTATTACTATCATTACCAGTACTCGCTGGAGCAATTACTATACTTTTAACTGACCGAAATTTTAA

>Sagami_Bay_Japan_(AB430547)

GTAATTGTTACAGCTCACGCATTTGTTATAATTTTTTTCCTTGTTATACCAGTTATAATCGGAGGATTTGGAAACTGATTAGTTCCTTTAATACTAGGAGCACCAGATATAGCATTCCCACGAATAAATAATATAAGCTTCTGACTCTTACCTCCTTCTCTTACTCTTCTCCTTTCATCTGCAGCAGTTGAAAGAGGCGCAGGTACCGGATGAACCGTTTACCCGCCTCTTTCAAGAAATTTAGCTCATATAGGACCTTCTGTTGATCTAGCCATTTTCTCACTTCACTTAGCAGGTATTTCATCAATCCTTGGAGCCATCAACTTTATTACAACTATTATTAATATACGATGAGAAGGTATATTAATAGAACGACTTCCACTATTTGTATGATCTGTATTTATTACCGCAATTTTACTATTACTATCATTACCAGTACTCGCTGGAGCAATTACTATACTTTTAACTGACCGAAATTTTAA

>Ria_Formosa_4_Portugal_(present_study)

GTAATTGTTACAGCTCACGCATTTGTTATAATTTTTTTTCTTGTTATACCAGTTATAATTGGAGGATTTGGAAACTGATTAGTTCCTTTAATACTAGGAGCACCAGATATAGCATTCCCACGAATAAATAACATAAGCTTCTGACTCTTACCTCCTTCTCTCACTCTTCTCCTTTCATCTGCAGCTGTTGAAAGTGGTGCAGGTACCGGATGAACCGTTTACCCACCTCTTTCAAGAAATTTAGCTCATATAGGACCCTCTGTTGATCTAGCAATTTTCTCACTTCACTTAGCAGGTATTTCATCAATTCTTGGAGCCATCAATTTTATTACAACTATTATTAATATACGATGAGAAGGTATATTAATAGAACGACTTCCACTATTTGTGTGATCTGTTTTTATTACCGCAATTTTACTATTACTATCATTACCAGTACTCGCTGGAGCAATTACTATACTTTTAACTGACCGAAATTTTAA

>Ria_Formosa_5_Portugal_(present_study)

GTAATTGTTACAGCTCACGCATTTGTTATAATTTTTTTTCTTGTTATACCAGTTATAATTGGAGGATTTGGAAACTGATTAGTTCCTTTAATACTAGGAGCACCAGATATAGCATTCCCACGAATAAATAACATAAGCTTCTGACTCTTACCTCCTTCTCTCACTCTTCTCCTTTCATCTGCAGCTGTTGAAAGTGGTGCAGGTACCGGATGAACCGTTTACCCACCTCTTTCAAGAAATTTAGCTCATATAGGACCCTCTGTTGATCTAGCAATTTTCTCACTTCACTTAGCAGGTATTTCATCAATTCTTGGAGCCATCAATTTTATTACAACTATTATTAATATACGATGAGAAGGTATATTAATAGAACGACTTCCACTATTTGTGTGATCTGTTTTTATTACCGCAATTTTACTATTACTATCATTACCAGTACTCGCTGGAGCAATTACTATACTTTTAACTGACCGAAATTTTAA

>Octopus_insularis_(KF844009)

GTAATTGTAACAGCACATGCATTTGTAATAATTTTTTTTCTAGTTATACCTGTTATAATTGGAGGATTTGGTAATTGATTAGTTCCTTTAATACTAGGAGCCCCAGATATAGCATTCCCACGAATAAATAACATAAGTTTTTGACTTTTACCACCTTCTCTTACCCTATTATTATCTTCAGCCGCAGTAGAAAGAGGTGCAGGTACCGGATGAACTGTATACCCTCCTCTTTCAAGAAATTTAGCCCATATAGGTCCTTCCGTTGATCTAGCTATTTTCTCCCTTCATTTAGCTGGTATTTCATCCATTCTTGGAGCCATTAATTTTATTACAACTATTATCAATATACGATGAGAAGGAATATTAATAGAACGACTCCCATTATTTGTATGAGCAGTATTTATTACTGCAATTTTATTACTACTCTCCTTACCAGTCCTCGCTGGAGCAATTACTATACTTTTAACAGATCGAAATTTTAA
