# Supplementary figures and images for: Octopus vulgaris (Cuvier, 1797) in the Mediterranean Sea: Genetic Diversity and Population Structure
Source: PLoS One. 2016 Feb 16;11(2):e0149496. doi: 10.1371/journal.pone.0149496 (PMC4755602; doi:10.1371/journal.pone.0149496)

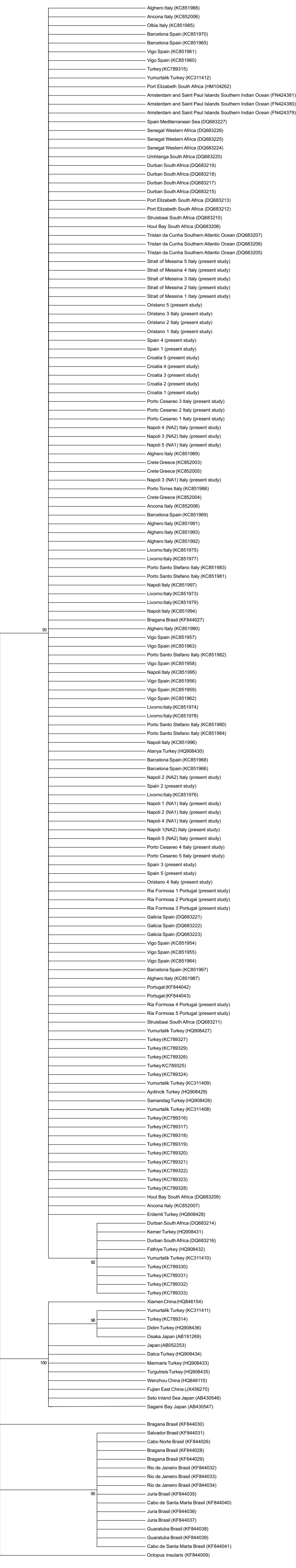

Supplement: S1 Fig — Values at the basis of each branch (node) indicate the percentage of occurrence of such specimens together with the other ones in the bootstrap test (1000 replicates). (PDF) [file pone.0149496.s003.pdf]

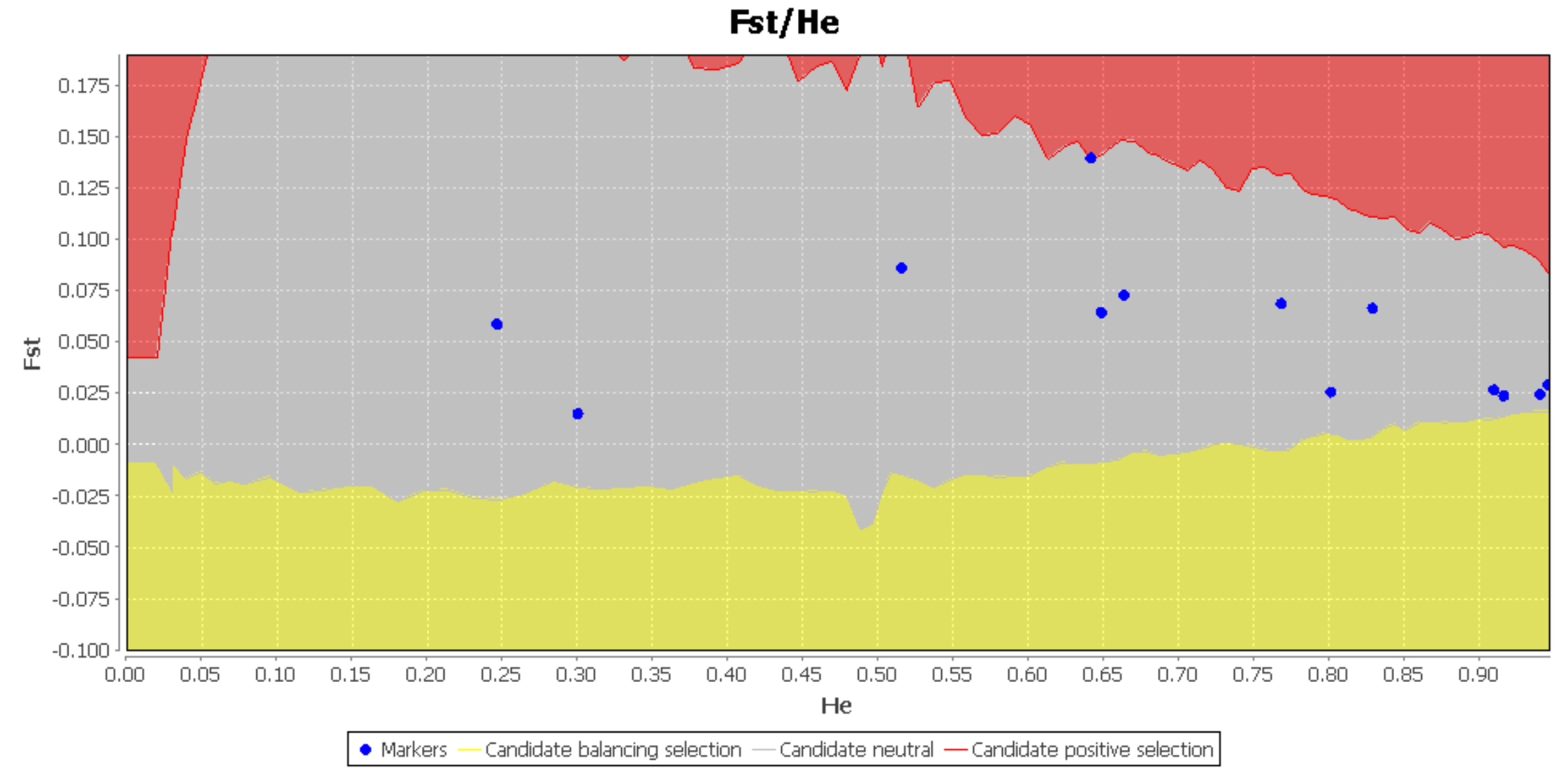

Supplement: S2 Fig — (TIF) [file pone.0149496.s004.tif]

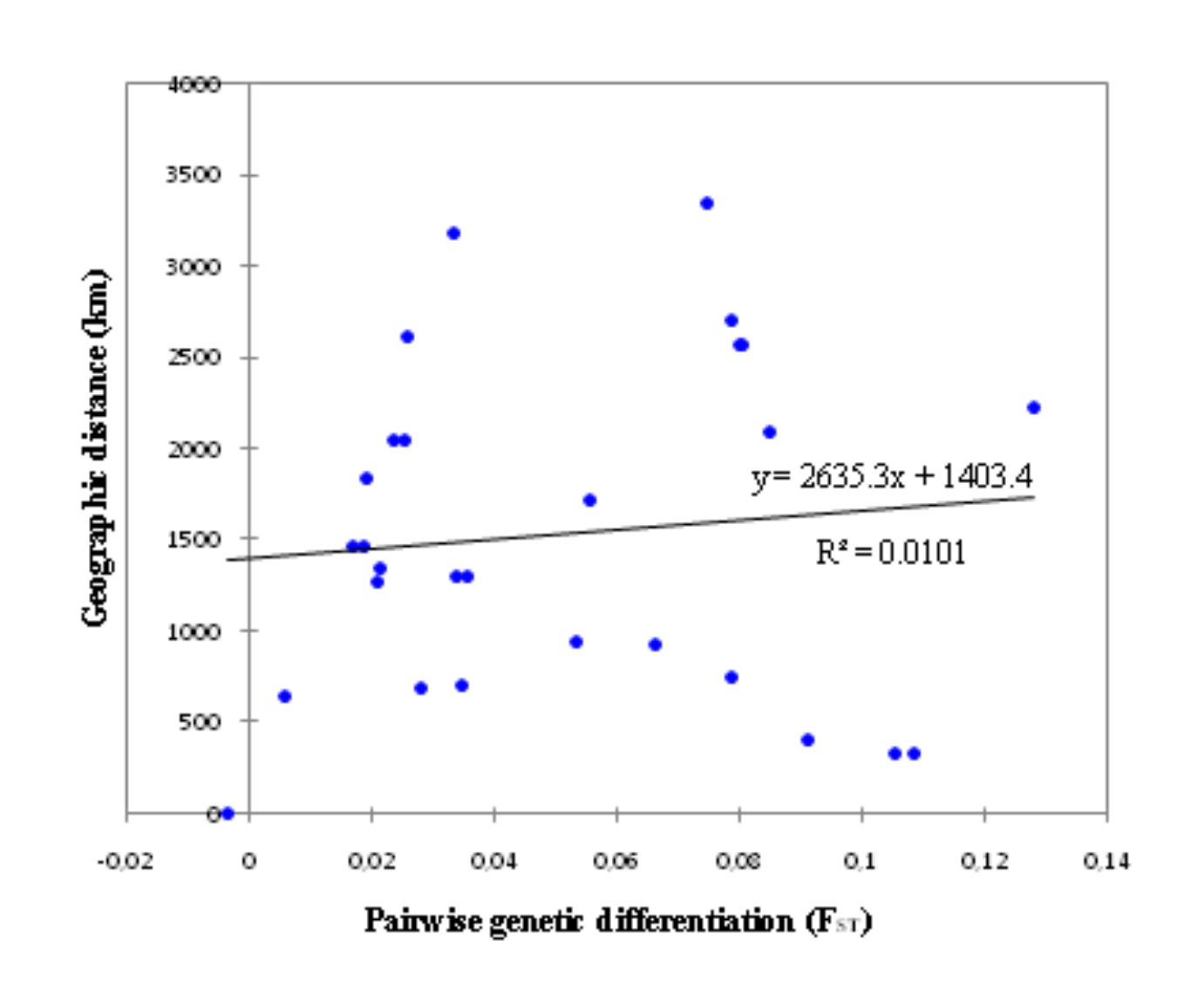

Supplement: S3 Fig — Correlation between geographic distance (km) and pairwise genetic differentiation (FST). (TIF) [file pone.0149496.s005.tif]

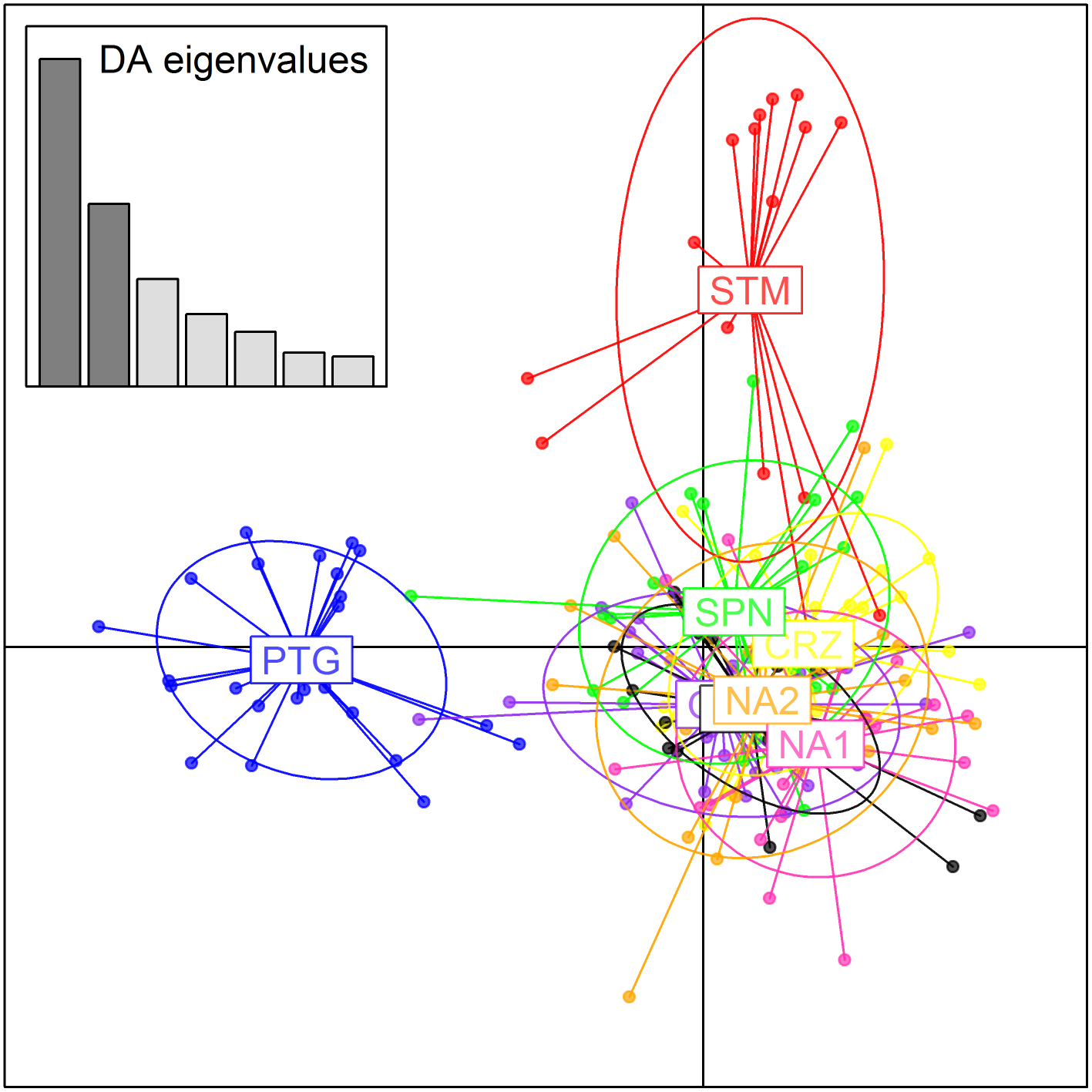

Supplement: S4 Fig — Geographic samples are encompassed by ellipses. See Table 1 for abbreviations. (TIF) [file pone.0149496.s006.tif]
