# Supplementary material for: Octopus vulgaris (Cuvier, 1797) in the Mediterranean Sea: Genetic Diversity and Population Structure
Source: PLoS One. 2016 Feb 16;11(2):e0149496. doi: 10.1371/journal.pone.0149496 (PMC4755602; doi:10.1371/journal.pone.0149496)
Supplement: S1 Table — (DOCX) [file pone.0149496.s007.docx]

**S1 Table. Null alleles frequency per locus.**

| **Locus** | **Null alleles frequency** |
| --- | --- |
| Vulg06 | 0.000000 |
| Vulg15 | 0.000000 |
| Vulg12 | 0.047841 |
| Vulg13 | 0.093591 |
| Vulg04 | 0.002715 |
| Vulg07 | 0.000000 |
| Vulg10 | 0.090386 |
| Vulg11 | 0.000000 |
| Vulg14 | 0.000001 |
| oct03 | 0.148701 |
| oct08 | 0.088428 |
| Ov10 | 0.000000 |
| Ov12 | 0.025400 |
