# Supplementary material for: Octopus vulgaris (Cuvier, 1797) in the Mediterranean Sea: Genetic Diversity and Population Structure
Source: PLoS One. 2016 Feb 16;11(2):e0149496. doi: 10.1371/journal.pone.0149496 (PMC4755602; doi:10.1371/journal.pone.0149496)
Supplement: S2 Table — Significance determined after Bonferroni adjustment at p < 0.000641. (DOCX) [file pone.0149496.s008.docx]

**S2 Table. Significant pair-wise linkage disequilibrium p-values.** Significance determined after Bonferroni adjustment at p < 0.000641.

| **Samples** | **Locus 1** | **Locus 2** | **P -value** |
| --- | --- | --- | --- |
| CRZ | Vulg12 | Oct08 | 0.000296 |
| CRZ | Vulg11 | Vulg12 | 0.000000 |
| CRZ | oct08 | Ov12 | 0.000000 |
| STM | Vulg06 | Vulg15 | 0.000000 |
| STM | Vulg06 | Vulg12 | 0.000000 |
| STM | Vulg15 | Vulg12 | 0.000000 |
| STM | Vulg06 | Vulg07 | 0.000000 |
| STM | Vulg15 | Vulg07 | 0.000000 |
| STM | Vulg12 | Vulg07 | 0.000000 |
| STM | Vulg13 | Vulg07 | 0.000580 |
| STM | Vulg06 | Vulg10 | 0.000000 |
| STM | Vulg15 | Vulg10 | 0.000143 |
| STM | Vulg07 | Vulg10 | 0.000271 |
| STM | Vulg06 | Vulg11 | 0.000000 |
| STM | Vulg15 | Vulg11 | 0.000000 |
| STM | Vulg12 | Vulg11 | 0.000000 |
| STM | Vulg07 | Vulg11 | 0.000000 |
| STM | Vulg06 | Ov10 | 0.000000 |
| STM | Vulg15 | Ov10 | 0.000000 |
| STM | Vulg12 | Ov10 | 0.000000 |
| STM | Vulg13 | Ov10 | 0.000000 |
| STM | Vulg07 | Ov10 | 0.000000 |
| STM | Vulg11 | Ov10 | 0.000000 |
| STM | Vulg06 | Ov12 | 0.000000 |
| STM | Vulg15 | Ov12 | 0.000000 |
| STM | Vulg12 | Ov12 | 0.000000 |
| STM | Vulg13 | Ov12 | 0.000310 |
| STM | Vulg07 | Ov12 | 0.000000 |
| STM | Vulg10 | Ov12 | 0.000462 |
| STM | Vulg11 | Ov12 | 0.000000 |
| STM | Ov10 | Ov12 | 0.000000 |
