# Supplementary material for: Octopus vulgaris (Cuvier, 1797) in the Mediterranean Sea: Genetic Diversity and Population Structure
Source: PLoS One. 2016 Feb 16;11(2):e0149496. doi: 10.1371/journal.pone.0149496 (PMC4755602; doi:10.1371/journal.pone.0149496)
Supplement: S3 Table — (DOCX) [file pone.0149496.s009.docx]

**S3 Table. Private alleles and their frequency in each geographic sample.**

| **Geographic**  **Sample** | **Locus** | **Allele** | **Frequency** |
| --- | --- | --- | --- |
| PTG | Vulg15 | 205 | 0.020 |
| PTG | Vulg15 | 227 | 0.020 |
| PTG | Vulg12 | 242 | 0.020 |
| PTG | Vulg12 | 262 | 0.040 |
| PTG | Vulg13 | 313 | 0.020 |
| PTG | Vulg13 | 323 | 0.020 |
| PTG | Vulg7 | 174 | 0.040 |
| PTG | Vulg7 | 232 | 0.020 |
| PTG | Vulg11 | 261 | 0.020 |
| PTG | Vulg11 | 271 | 0.020 |
| PTG | oct03 | 117 | 0.080 |
| PTG | oct08 | 126 | 0.040 |
| PTG | oct08 | 176 | 0.020 |
| PTG | Ov10 | 174 | 0.040 |
| PTG | Ov10 | 200 | 0.080 |
| PTG | Ov10 | 202 | 0.040 |
| PTG | Ov12 | 284 | 0.020 |
| PTG | Ov12 | 304 | 0.020 |
| PTG | Ov12 | 308 | 0.040 |
| PTG | Ov12 | 312 | 0.060 |
| PTG | Ov12 | 324 | 0.020 |
| PTG | Ov12 | 340 | 0.020 |
| PTG | Ov12 | 380 | 0.020 |
| CRZ | Vulg7 | 208 | 0.020 |
| CRZ | Vulg11 | 257 | 0.020 |
| CRZ | oct03 | 129 | 0.040 |
| STM | oct03 | 131 | 0.176 |
| STM | Ov12 | 336 | 0.029 |
| ORI | Vulg7 | 230 | 0.020 |
| ORI | Vulg11 | 225 | 0.040 |
| ORI | Vulg14 | 409 | 0.020 |
| ORI | oct03 | 193 | 0.022 |
| ORI | oct08 | 168 | 0.020 |
| PCS | oct08 | 124 | 0.020 |
| PCS | oct08 | 170 | 0.020 |
| PCS | oct08 | 174 | 0.040 |
| PCS | Ov12 | 232 | 0.040 |
| PCS | Ov12 | 320 | 0.020 |
| SPN | Vulg7 | 150 | 0.038 |
| SPN | Vulg7 | 218 | 0.038 |
| SPN | Vulg14 | 355 | 0.019 |
| SPN | oct08 | 114 | 0.020 |
| NA1 | Vulg4 | 151 | 0.020 |
| NA1 | Vulg11 | 229 | 0.020 |
| NA1 | Vulg11 | 237 | 0.020 |
| NA1 | oct03 | 125 | 0.040 |
| NA1 | oct08 | 112 | 0.040 |
| NA1 | Ov12 | 236 | 0.020 |
| NA2 | Vulg10 | 207 | 0.020 |
| NA2 | Vulg10 | 219 | 0.020 |
| NA2 | Vulg11 | 259 | 0.020 |
| NA2 | Vulg11 | 267 | 0.020 |
| NA2 | oct03 | 115 | 0.040 |
| NA2 | oct08 | 116 | 0.100 |
| NA2 | Ov10 | 170 | 0.020 |
