# Supplementary material for: Octopus vulgaris (Cuvier, 1797) in the Mediterranean Sea: Genetic Diversity and Population Structure
Source: PLoS One. 2016 Feb 16;11(2):e0149496. doi: 10.1371/journal.pone.0149496 (PMC4755602; doi:10.1371/journal.pone.0149496)
Supplement: S4 Table — Abbreviations are as follows: U = unrelated, HS = half- sibs, FS = full-sibs, PO = parent/offspring. In italics are values corrected for null alleles. (DOCX) [file pone.0149496.s010.docx]

**S4 Table. Relationships among individuals.** Abbreviations are as follows: U = unrelated, HS = half- sibs, FS = full-sibs, PO = parent/offspring. In italics are values corrected for null alleles.

| **Geographic sample** | **N individuals** | **HS** | **FS** | **PO** |
| --- | --- | --- | --- | --- |
| **PTG** | 25 | 0.24333  *0.04667* | 0.01000  *0.00333* | 0  *0* |
| **SPN** | 26 | 0.06769  *0.05846* | 0.00308  *0.00923* | 0  *0* |
| **ORI** | 25 | 0.04333  *0.06333* | 0  *0* | 0  *0* |
| **NA1** | 25 | 0.03000  *0.03667* | 0  *0* | 0  *0* |
| **NA2** | 25 | 0.01667  *0.03667* | 0.00333  *0* | 0  *0* |
| **STM** | 17 | 0.00735  *0.00735* | 0.22059  *0.22059* | 0.00735  *0.00735* |
| **PCS** | 25 | 0.04667  *0.05000* | 0.00333  *0.00333* | 0  *0* |
| **CRZ** | 25 | 0.03667  *0.04667* | 0.01667  *0.01667* | 0  *0* |
