# Supplementary material for: Octopus vulgaris (Cuvier, 1797) in the Mediterranean Sea: Genetic Diversity and Population Structure
Source: PLoS One. 2016 Feb 16;11(2):e0149496. doi: 10.1371/journal.pone.0149496 (PMC4755602; doi:10.1371/journal.pone.0149496)
Supplement: S5 Table — Asterisks indicate the p value of each pair-wise comparison: * p < 0.05; ** p < 0.01; *** p < 0.001; ns = not significant. In bold values significant after Bonferroni correction at p < 0.0017672. See Table 1 for abbreviations. (DOCX) [file pone.0149496.s011.docx]

**S5 Table**. **Matrix of pair-wise *F*_ST_ (below the diagonal) and Jost’s D (above the diagonal).** Asterisks indicate the p value of each pair-wise comparison: * p < 0.05; ** p < 0.01; *** p < 0.001; ns = not significant. In bold values significant after Bonferroni correction at p < 0.0017672. See Table 1 for abbreviations.

|  | **PTG** | **SPN** | **ORI** | **NA1** | **NA2** | **STM** | **PCS** | **CRZ** |
| --- | --- | --- | --- | --- | --- | --- | --- | --- |
| **PTG** | - | **0.13073***** | **0.13117***** | **0.17371***** | **0.16039***** | **0.37042***** | **0.14822***** | **0.18022***** |
| **SPN** | **0.03209***** | - | 0.00579^ns^ | 0.02297^ns^ | 0.01683^ns^ | **0.19724***** | 0.02499^ns^ | 0.05751** |
| **ORI** | **0.03421***** | 0.01105* | - | 0.01700^ns^ | 0.01826^ns^ | **0.21865***** | 0.02523^ns^ | 0.04613** |
| **NA1** | **0.05545***** | **0.01607***** | **0.01815***** | - | -0.01076^ns^ | **0.27075***** | 0.01469^ns^ | 0.06458** |
| **NA2** | **0.04967***** | 0.01208** | **0.01947***** | -0.00546^ns^ | - | **0.26157***** | 0.01274^ns^ | 0.06722** |
| **STM** | **0.10478***** | **0.07011***** | **0.06583***** | **0.09058***** | **0.08663***** | - | **0.21164***** | **0.16337***** |
| **PCS** | **0.05109***** | 0.01401** | 0.00874^ns^ | 0.00954* | 0.01049* | **0.06915***** | - | 0.00748^ns^ |
| **CRZ** | **0.05377***** | **0.02451***** | 0.01084* | **0.02433***** | **0.02562***** | **0.05307***** | -0.00020^ns^ | - |

Bonferroni value 0.0017672 in red
