# Supplementary material for: Octopus vulgaris (Cuvier, 1797) in the Mediterranean Sea: Genetic Diversity and Population Structure
Source: PLoS One. 2016 Feb 16;11(2):e0149496. doi: 10.1371/journal.pone.0149496 (PMC4755602; doi:10.1371/journal.pone.0149496)
Supplement: S6 Table — Values in italics refer to the dataset corrected for null alleles. (DOCX) [file pone.0149496.s012.docx]

**S6 Table. Contemporary effective population size (Ne) based on linkage disequilibrium and molecular co-ancestry models, with relative confidence intervals (CIs).** Values in italics refer to the dataset corrected for null alleles.

| **Group** | **Geographic**  **sample** | **Sample**  **size** | **Estimated Ne**  **LD method** | **95% CI**  **for Ne** | **Estimated Ne**  **Molecular Coancestry**  **method** | **95%CI**  **for Ne** |
| --- | --- | --- | --- | --- | --- | --- |
| 1 | PTG | 25 | 86  *170* | 55 - 174  *86 - 1881* | 8  *8* | 2  *3* |
| 2 | CRZ, PCS | 50 | 316  *233* | 169 - 1690  *141 - 597* | infinite  *273* | infinite  *0* |
| 3 | STM | 17 | 2  *3* | 2 - 3  *2 - 3* | 8  *6* | 2  *3* |
| 4 | SPN, ORI,  NA1, NA2 | 101 | 1504  *1099* | 511 - infinite  *439 - infinite* | 22  *28* | 8  *5* |
